# Readiness to Consider and Adopt Genetic and Genomic Tests in Canada—An Update to the State of Readiness Report

**DOI:** 10.3390/curroncol33060334

**Published:** 2026-06-04

**Authors:** Don Husereau, Filomena Servidio-Italiano, Monika Slovinec D’Angelo, Vivek Muthu, Michael Mengel, Craig Ivany, Lotte Steuten, Daryl S. Spinner, Brandon Sheffield, Stephen Yip, Philip Jacobs, Larry Arshoff

**Affiliations:** 1 School of Epidemiology and Public Health, University of Ottawa, Ottawa, ON K1G 5Z3, Canada; 2 Colorectal Cancer Resource & Action Network (CCRAN), Toronto, ON M4W 3E2, Canada; filomena.s@ccran.org; 3 School of Psychology, University of Ottawa, Ottawa, ON K1N 6N5, Canada; monslovinec@gmail.com; 4 Marivek Healthcare Consulting, Epsom KT18 7PF, UK; vivmuthu@gmail.com; 5 Department of Laboratory Medicine & Pathology, University of Alberta, Edmonton, AB T6G 2S2, Canada; mmengel@ualberta.ca; 6 Department of Pathology & Laboratory Medicine, Faculty of Medicine, University of British Columbia, Vancouver, BC V6T 1Z7, Canada; craig.ivany@ubc.ca (C.I.); stephen.yip@vch.ca (S.Y.); 7 Office of Health Economics, London SE1 2HB, UK; lsteuten@ohe.org; 8 City Health Economics Centre, St. George’s University of London, London EC1V 0HB, UK; 9 Predicta Biosciences, 750 Main St, Cambridge, MA 02139, USA; daryl.spinner@predictabiosciences.com; 10 William Osler Health System, Brampton, ON L6R 3J7, Canada; brandon.s.sheffield@gmail.com; 11 Faculty of Medicine and Dentistry, University of Alberta, Edmonton, AB T6G 2R3, Canada; philjacobs@shaw.ca; 12 Diagnosis, Solutions & Results Inc., Thornhill, ON L4J 7N5, Canada; larry.arshoff@dsandr.com

**Keywords:** diagnostic molecular pathology, genetic testing, diagnostic services, technology assessment, biomedical, genetic services, financial support, clinical governance, health technology, healthcare innovation

## Abstract

(1) Background: Genomic medicine—i.e., the use of laboratory-based biomarkers that measure the expression, function and regulation of genes and gene products to aid healthcare decision making is a rapidly emerging technology. Readiness to consider and adopt new testing programs effectively and avoid critical challenges requires health systems to harbor a number of key conditions that address infrastructural, as well operational and other needs. This assessment re-examines Canada’s state of readiness since a previously published 2023 assessment. (2) Methods: A mixed-methods approach of a review of the literature and key informant interviews with a purposive sample of experts was used. Health system readiness was assessed using a previously published set of conditions. (3) Results: This updated analysis of Canada’s state of readiness for genetic and genomic testing reveals Canada is only partially ready for a future of genomic medicine, although some progress has been made since 2023. The most established conditions were the use of appropriate service models and the integration of innovation and healthcare delivery functions. They suggest that Canada’s major healthcare regions are moving closer to a state of readiness for the consideration and adoption of new testing required for genomic medicine, although using different approaches and at different rates. These findings should be seen as generalizable to other regions internationally—health systems need to have functions that promote responsiveness and resilience, i.e., are able to recognize valuable innovation and quickly shift priorities and create conditions necessary to enable it.

## Simple Summary

Health systems worldwide need to be prepared for integrating genomic and genetic information into healthcare. This requires necessary conditions for its consideration and adoption. Our scan of Canadian health systems show they are partly prepared and have made progress since 2023. Areas of strength included developing good service models and connecting innovations with healthcare delivery. Individual regions in Canada are getting closer to considering and adopting genetic and genomic testing well, but they are moving at different speeds and using different approaches. Health systems that are the most flexible and responsive are able to adjust quickly when useful new tests become available.

## 1. Introduction

The diffusion (consideration, adoption, and widespread implementation) of health technology relies on a number of factors, including health system characteristics (e.g., size, maturity and change leadership functions) and other enabling conditions for managing implementation (e.g., evaluative functions, flexible finance approaches and appropriate infrastructure) [1,2,3]. These factors are particularly important for technology that relies on the timing, expertise, and behavior of different health system actors to be delivered effectively and efficiently [4].

Genomic medicine is one such rapidly emerging technology. It involves identifying and measuring important aspects of genes and gene products to aid healthcare decision making [5]. In order for patients to benefit, health systems must decide on various aspects of technology implementation including what technical platforms use (e.g., single-gene, multi-gene, whole-exome, and whole-genome sequencing and expression analysis); how samples are collected (e.g., urine, tissue, saliva, blood, cerebrospinal fluid); where to analyze samples (e.g., in a laboratory or at point-of-care); how tests are developed and delivered (e.g., using commercial products and services versus laboratory-developed tests); and how tests are incorporated into care pathways.

Beyond better health outcomes, readiness to consider and adopt genomic medicine has the real potential to enhance patient experiences of care, improve the working lives of healthcare clinicians and staff, and reduce the per capita cost of healthcare—healthcare’s quadruple aim [6,7]. It also provides opportunities for scientific discovery and economic growth given its central role in medical research including qualifying individuals for clinical investigation.

Readiness to consider and adopt new testing programs effectively and avoid critical challenges also requires health systems to harbor a number of key conditions that address infrastructural, as well operational and other needs (Table 1) [8]. In Canada, individual provinces and territories are responsible for delivering diagnostic tests and, therefore, health system readiness in Canada requires assessment of 10 individual provincial and 3 territorial systems responsible for healthcare delivery [9].

Recognizing the need for health systems to recognize and foster these conditions, a “State of Readiness” progress report was developed in 2023 [10]. Five provincial health systems representing 90% of Canadians were assessed. A number of important gaps were identified including the need for better systems of information management, technology assessment processes and navigational tools for care providers. There were also deficiencies in funding for more rapid onboarding including support for test development and proficiency testing. Across health systems there was little engagement with innovation stakeholders beyond care providers.

This assessment revealed that some Canadian provincial health systems, were at a nascent stage, but were working toward readiness [10]. To see if further progress has been made, an update to the report, including an expansion of the assessment to all provincial and territorial healthcare systems, has been conducted. As with the previous report, the purpose of this research was to facilitate better understanding of Canada’s readiness to consider and adopt genomic and genetic testing, and provide insights into the possible barriers and facilitators of readiness for other healthcare systems.

## 2. Materials and Methods

While the intent was to assess all Canadian provincial and territorial healthcare jurisdictions, four jurisdictions (Prince Edward Island, Northwest Territories, Nunavut, and Yukon) provide testing services through out-of-province/territory referrals to other provinces (see Box 1). As such, they are excluded from this analysis. The state of readiness for the remaining provinces was assessed using a mixed-methods approach involving a narrative review of the literature and semi-structured interviews with organizational representatives. The review included both commercially published and gray literature including health ministry and healthcare system websites. Genomic medicine, advanced testing, genetic testing, and genome-based testing are used interchangeably throughout this report.

Box 1 **Provinces and territories referring to other centers.**

Prince Edward Island

Prince Edward Island (PEI) is the smallest Canada’s 13 provinces by size and population (approx. 0.18 million). Genetic testing is performed on a referral basis to centers in Nova Scotia.

Northwest Territories

Northwest Territories (NWT) is the second largest territory in size and second largest in population (approx. 45,000). Genetic testing is performed on a referral basis to centers in Alberta.

Nunavut

Nunavut (NU) is the largest territory in size and third largest in population (approx. 41,000). Genetic testing is performed on a referral basis to centers in Ontario, Manitoba, and Alberta.

Yukon

Yukon is the smallest territory in size and largest in population (approx. 47,000). Genetic testing is performed on a referral basis to centers in British Columbia and Alberta.

Semi-structured interviews with provincial representatives (*n* = 36; 30–60 min) were conducted using a qualitative descriptive approach, defined as a naturalistic inquiry wherein the interviews and resultant data furnish a rich, straightforward depiction of an experience or event. Interview transcripts were coded and categorized to facilitate conventional content analysis. All interviews were performed by D.H., with a purposive sample of experts representing the commercial life sciences sector (*n* = 14), academia/research personnel (*n* = 2), healthcare providers (*n* = 14), healthcare leadership/administrators (*n* = 5), and patients (*n* = 1). Participants were selected based on heterogeneous expertise and geographic locations, with fifteen (*n* = 15) having previously collaborated with the author.

Interviews were conducted via recorded video conference calls equipped with audio recording features and automated transcription capabilities. An interview guide (see Appendix A) was developed and pilot-tested; all but of the two invitees who were approached—both healthcare providers—agreed to participate. For member checking, participants received summary notes derived from transcripts for verification.

All data, encompassing audio recordings, transcripts, and digital field notes, were securely stored on a password-protected drive. Automated transcriptions underwent verification against original audio files and were subsequently corrected. Participants were instructed to furnish organizational perspectives, with no foreseeable risk of personal harm; thus, formal ethics approval was not sought.

As in the previous report, information from the literature review and interviews was synthesized and evaluated against previously published conditions for health system readiness. These conditions embody three domains (infrastructure, operations, and healthcare environment) and are further described in the Section 3. Data privacy is governed by federal legislation, which makes it the same across jurisdictions so was excluded from the analysis. Necessary components of each condition within each jurisdiction were mapped out (See Appendix A), and their status was classified as ‘needs improvement’ (i.e., non-existent or nascent functional components), ‘partially established’ (i.e., some functional components required to create the condition present), or ‘established’ (most or all components needed for the condition present). Components encompassing good practice were given equal weight. Full descriptions of this mapping exercise including the status of each condition were shared back with provincial representatives for verification.

## 3. Results

### 3.1. Canada

This updated analysis of Canada’s state of readiness for genetic and genomic testing reveals Canada is only partially ready for a future of genomic medicine, although some progress has been made since 2023. The most established conditions were the use of appropriate service models and the integration of innovation and healthcare delivery functions. Many important gaps still remain: these include evaluative processes that adhere to principles of timeliness transparency and engagement recognized as good practice in health technology assessment (HTA); the need for good practices in information management including data integration and linked information systems; navigational tools for care providers; the need for better financing approaches including funding to develop testing or a payment model for the reimbursement of test services that incents high quality care as well as funding for proficiency testing; and better engagement with stakeholders including patients, administrators, information and communication technology professionals, implementation and genome scientists and public and private sector innovators (Table 2).

Canadian provinces better prepared to consider and adopt genomic medicine are Alberta and Newfoundland. Both provinces exhibit a higher level of transparency in approach as well as having dedicated infrastructure for coordination and planning. Ontario was observed to make the most progress since 2023, due to the establishment of a provincial genetics program (PGP) within their single healthcare system (Ontario Health). The PGP facilitates resource planning and coordination of care for Ontario including the publication of a biomarker test menu. The PGP has also created some province-wide standards for education and training.

Nova Scotia made the second largest degree of progress due to improvements in information linkage and evaluative process for new proposals as well as some improvements in education and regulation (quality) of service delivery. While there have also been incremental improvements in Alberta, British Columbia, and Quebec, these changes were considered minor.

**Figure 1:**
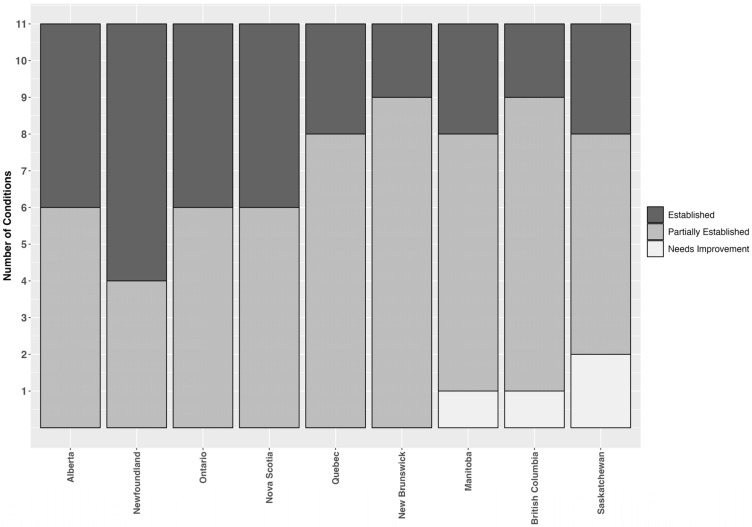
Number of conditions for readiness for genome-based medicine by province. The lightest shade indicates the condition does not exist or needs significant improvement (‘needs improvement’). The darkest shade indicates most or all aspects of the necessary condition were established (‘established’). Intermediate shade indicates some aspects of the condition were established (‘partially established’).

A visual representation of the number of established conditions in each province is in Figure 1. Section 3.2 provides more detail, and a complete assessment of each province can be found in a larger report [13] in Supplementary File S1.

### 3.2. Strengths and Weaknesses Across Provinces

#### 3.2.1. Infrastructure and Planning

Conditions for infrastructure and planning include formal communities of practice that engage all stakeholders; a comprehensive and systematic resource planning and oversight function, (involving budgeting based on horizon scanning for anticipated resource flows) as well as planning to ensure those resources can be acquired; and information management that includes linked laboratory information systems readily accessible by specialists [8]. Most provinces have created single service organizations that support these conditions, including an oversight function for genomic and genetic testing through resource planning, and creating communities of practice. These organizations often exist as entities within healthcare systems and can be more nimble than Ministry-led programs due to more immediate access to operational resources. They are also more effective in provinces with health systems that are integrated (i.e., single health regions), rather than regionalized.

In Alberta, for example, Alberta Precision Laboratories (APL) is operated by Alberta’s single health authority, Alberta Health Services (AHS). Highly specialized genomic/genetic testing is delivered as a provincial program within APL directly reporting to executive leadership. Similar organizations exist in British Columbia (the Provincial Laboratory Medicine Service), Manitoba (Diagnostic Services), Newfoundland and Labrador (The Laboratory Medicine Program), Nova Scotia (Pathology and Laboratory Medicine Program), Ontario (Pathology & Laboratory Medicine Program and Provincial Genetics Program), Quebec (Direction de Laboratoires et Imagerie Médicale, DLIM), and Saskatchewan (Laboratory Medicine Program). Only New Brunswick still relies on its provincial Department of Health to provide an oversight function. Until recently, Ministry-led functions were also in place in Quebec, and Ontario.

Laboratory service organizations were also observed to facilitate intra-provincial coordination and the development of communities of practice. In Quebec, for example, the OPTILAB initiative created a network of laboratory clusters for service delivery that, in turn, led to the formation of more specialized networks such as the Réseau Québécois de Diagnostic Moléculaire (RQDM) [14]. While formal and informal networks exist within each province and, facilitated by service organizations, broader formal engagement with industry, patients and other stakeholder groups by these organizations are almost non-existent. This is likely reflective of the lack of engagement with industry and health systems in general and the lack of engagement with patients at a service-delivery level. Patient engagement in healthcare in Canada is typically seen at very high levels through regional or supra-regional advisory councils and locally at hospital levels. Individual laboratory service providers informally engage with specific companies, but formal engagement with industry representatives and laboratory service organizations is lacking.

Efforts to improve digital infrastructure and necessary conditions for information management were observed to go beyond the remit of these laboratory service organizations, with efforts led at a Ministry or health system level. As a result, the need for improvements in information management was still required in almost all provinces (BC, MB, NB, NS, ON, QC, SK) despite consolidated laboratory service organizations. Many efforts to improve information linkage were observed to be ongoing, and are part of larger health-system-level initiatives that will take many years. For example, British Columbia’s plan to create a connected health system began in 2019 with its Ministry-led Digital Health Strategy [15]. The strategy was finalized in 2024 with broad implementation expected in the next year to two years. This has led to some laboratory information systems being linked while (as of 2025), integration of genetic laboratory information systems is planned but still underway.

#### 3.2.2. Operations

Operational readiness was defined by four separate conditions, including: having a single-entry point for new proposals for innovation open to all stakeholders; having a robust evaluation function that aligns with good principles in health technology assessment; a standardized service model; and navigation supported by a comprehensive test list with some information describing how tests can be accessed and what expected turnaround times can be expected [8]. These conditions are similarly supported by laboratory service organizations, but good practices were lacking in most provinces.

The only province observed to have a single-entry point open to all stakeholders was Newfoundland and Labrador. This entry point was also accompanied by a transparent description of the process for considering and adding new tests, including clear criteria and a timeline. Other provinces with clear application procedures include BC, AB, and QC but require applicants to be laboratory leaders or clinicians. Other provinces have much less transparent application processes despite key informants indicating that these processes do exist.

While some provinces harbored clear application procedures, none had a subsequent evaluation process for new tests that completely aligns with good practices in health technology assessment. This includes exhibiting principles of transparency, timeliness, and impartiality coupled with a clear deliberative process that engages key stakeholders [16,17]. The leading examples of provinces with formal evaluation procedures include Alberta, Ontario and Quebec but even these processes have significant shortcomings. For example, the Institut National d’Excellence en Santé et en Services Sociaux (INESSS) in the province of Québec has a process that is transparent, impartial, and engages patients and clinicians but does not engage industry or adhere to strict timelines. The Alberta Precision Laboratories and Ontario Provincial Genetics Program processes are far less transparent—there are no published processes or terms of reference including committee membership and timelines. Ontario also hosts separate HTA processes (the Ontario Health Technology Advisory Committee and Ontario Genetics Advisory Committee). While transparent and engaging, these processes are far from timely. A 2024 assessment of “Plasma-Based Comprehensive Genomic Profiling DNA Assays for Non-Small Cell Lung Cancer” took two years to complete, much longer than the average length of survival in the patients that might benefit from the test [18].

Most provinces had a formal or informal model of service standardization and coordination, although many provinces (AB, BC, MB, NB, NS, QC, SK) did not provide a comprehensive list of tests or information regarding test ordering and turnaround for service providers. This means laboratories may have a clear understanding of who can conduct a test as well as accompanying service parameters (e.g., what information is provided to care providers and how it is interpreted) but service providers may not have the same level of understanding. It also creates a deficit in understanding for those wanting to propose the adoption of new tests, creating a situation where a clinician or innovator feels there could be an unmet need, when in fact the test has already been considered. Ontario and Newfoundland were the only provinces with publicly accessible, comprehensive and up-to-date test menus with additional descriptive information for those ordering tests.

#### 3.2.3. Healthcare Environment

Healthcare environment conditions were those that related to readiness for innovation through translational research programs; a nimble and transparent finance approach including funding for test development and discretionary spending for new tests; systematic training or a training strategy with province-wide educational standards or guidance; and regulatory standards for analytic validity and proficiency across labs. All provinces have adopted ISO 15189 province-wide standards to ensure laboratory competence in producing accurate, reliable test results [8]. All provinces have also established or partially established conditions related to readiness for innovation. There are differences across provinces regarding the reporting of tests that have less well-defined clinical utility (i.e., “investigational” testing).

Of all provinces only Quebec had established an appropriate condition for financing new tests, with a clear funding formula, additional funding for test development and human resource costs. In some provinces (NB, NL) test development was covered based on business cases for new testing provided by laboratories. This situation can lead to slower processes of adoption, depending on how far ahead laboratory leaders anticipate the need for new tests coupled with the timing of government approval processes. In other provinces, funding for test development was required to come from other budgets or through partnerships with the private sector. Relying on the private sector creates the risk of a “private finance initiative” problem where health system priorities for testing are dictated by who is paying, rather than unmet need, equity, or efficiency [19].

Similarly, only one province (Ontario) had a more established approach to the education of care providers and patients. This is because developing a strategy for education and creating standards for education was a priority of Ontario’s Provincial Genetics Program since its inception. Education resources for patients, genetics professionals, and healthcare professionals are now accessible on the Ontario Health website. The province of Quebec is developing similar standards. Most provinces rely on lab-motivated training reliant on private or grant funding, reintroducing the problem of educational priorities being dictated by who is paying rather than reflecting health system priorities.

## 4. Discussion

Overall, these findings suggest that Canada has continued to establish necessary conditions for the readiness to consider and adopt new genetic and genomic tests but there is more to be done. Our findings suggest some key areas requiring improvement. Those that are most feasible to improve include: how new testing proposals are considered and evaluated (i.e., having an open application process coupled with a robust HTA approach); having navigational aids including a comprehensive test list and instructions for ordering tests, and providing resources to support the use of tests, informed by overarching education strategies. Additional areas for improvement that may be more difficult to achieve due to a reliance on broader health system conditions include the need for more robust financing approaches including funding for test development, and broader stakeholder engagement including engagement with industry in planning service delivery.

While many provinces appear more ready than ever to consider and adopt new testing, some still appear to be less ready to execute (i.e., deliver and monitor) testing in a consistent, high-quality manner due to a wide variety of factors. These factors range from a lack of clarity regarding care pathways and optimized workflows, to poor communication across providers (e.g., from a lack of integrated information systems), to inconsistent knowledge across providers (in part due to a lack of education, awareness and navigational resources), to a lack of specific human resources (e.g., such as laboratory technologists). The effectiveness and efficiency of service delivery is outside of the scope of this report and remains an area for future research. This avenue of research would help to provide a full picture of the state of readiness for genomic medicine in Canada.

A strength of these findings is that it captures information about Canada as a whole. While the previous report surveyed five provinces which represent ~90% of the population, it still only represented the minority (5/13) of provinces and territories. One province and three territories have no in-house capacity to provide testing. Health services are provided to their residents through cross-billing arrangements with other provinces (see Box 1). This updated assessment still highlights the challenges of federated (pluralistic) health systems and the advantages of choices in governance. Provinces with single healthcare systems and single service laboratory providers were better positioned to meet conditions including: (1) creating communities of practice and regional networks; (2) systematic oversight and resource planning; (3) single points of entry for new testing proposals; (4) an evaluation function; (5) coordination of service; (6) tools for awareness and care navigation; (7) standards for education and training; and (8) regulatory standards related to analysis.

As an example, the province of Ontario has made considerable progress since 2023 due to establishment of provincial genetics program (PGP) which acts as an established link to the Ontario Pathology and Laboratory Medicine program [20]. This program facilitates resource planning and coordination of care for Ontario including the publication of test menus [21]. The PGP has also created province-wide standards for education and training. Examining Ontario’s progress was still a challenge, however, due to the continued non-transparency of its underlying approaches. Key informants indicated that test menus were developed by explicit prioritization and deliberative processes based on pre-established criteria, but details were not available. In contrast, the province of Newfoundland and Labrador features a website that describes in detail how new tests are considered and what tests are valued by the healthcare system [22].

A potential limitation of the findings is that it relied on interviews with organization representatives. While we did seek representatives working at the highest levels of each organization (e.g., directors and heads of laboratories), it is possible that some information was not accurately conveyed or understood by the single researcher conducting the interviews.

Transparency is an often overlooked contributor to the health and wealth of societies as it clearly signals to healthcare providers, private sector innovators, and the public (that finances healthcare) what is valued and what is needed [23,24,25,26]. While it has been identified as a key principle in HTA [27], most processes for evaluation in Canada were observed to be non-transparent. Recent work by Canada’s Drug Agency (CDA-AMC) highlights the need for transparency and accountability as well as collaboration, cooperation and engagement across all Canadian health systems as a means to create high quality and consistent HTA processes for biomarkers in Canada [28]. The CDA-AMC framework also outlines an approach to coordination and prioritization across provinces, as well as advocating for consistent criteria for evaluation, based on the ACCE framework [29]. British Columbia has recently adopted this framework through the initiation of a new Oncology Testing Committee (OTC) which evaluates submissions for requests for new molecular tests in the BC Cancer system.

Our findings reveal financing approaches vary widely, and can be a barrier to rapid adoption in many healthcare systems. A potential challenge in this area is that tests are often not entirely funded through a single budget. Funding for any given genetic or genomic test may rely on a number of financing entities including hospital operations, private and public research grants, funding from charities, funding from private service delivery, and funding from special programs, such as cancer care. Only the four largest provinces (AB, BC, ON, and QC), as well as Saskatchewan and Nova Scotia reported having discretionary funds for the delivery of new testing. Funding for test development is still largely reliant on research grants, often from private life science companies who require testing to identify patients who will go on to use a new targeted therapy. While this can be an expedient approach it can also pose challenges insofar as priority setting for new tests will rely on who is paying, rather than systemic need.

As with the 2023 report, these findings are a snapshot at a point of time. Findings were validated with key informants in June 2025 and may not capture further significant developments across healthcare systems. Despite this, the pace of development and government priorities are usually relatively slow-moving and based on year-to-year plans and should be considered generally accurate. As with the previous report we believe the conditions used to gauge health system readiness could be used in other jurisdictions as well as be applicable to future advanced testing including transcriptomics, proteomics, metabolomics, and epigenetics.

## 5. Conclusions

These findings suggest that Canada’s major healthcare regions are moving closer toward a state of readiness for the consideration and adoption of new testing required for genomic medicine, although using different approaches and at different rates. This report highlights the many challenges that health systems face when they are required to quickly respond to disruptive technology. Even more so, this report highlights the differences in access to care that Canadians may face when they are served by individual health regions with different priorities and healthcare structures. The challenges outlined in this analysis are not unique to Canada; they are shared globally, even by countries viewed as among the most advanced in terms of access to genetic and genomic testing (e.g., the United Kingdom, the United States). Patients will benefit most from healthcare systems that are best able to anticipate and adapt to changes from disruptive and complex healthcare technology. The report suggests that healthcare systems need to have functions that promote responsiveness and resilience, i.e., are able to quickly shift priorities and able to recognize and enable high-value innovation.

## Figures and Tables

**Table 1:** Necessary conditions for healthcare system preparedness for genome-based testing from reference [8,10].

Health System Challenge	Condition(s) Required	Good Practice Description
Care interruptions, wait times, and unsustainable care	(1) Resource planning	Frequent and systematic resource planning
	(2) Finance model	Nimble and transparent funding formula (i.e., payment that accounts for development and human resource costs and benefits)
Inequitable care delivery	(3) Creating communities of practice and healthcare system networks	Engagement with all stakeholders, including administrators, information and communications technology professionals, implementation and genome scientists, and public and private sector innovators
Uncoordinated or duplicative care	(4) Information management *	Integrated laboratory information systems and electronic health records
	(5) Tools for awareness and care navigation	Available, up-to-date information and navigation support
	(6) Tools for education and training	Educational standards that address continuing professional development, knowledge updates, and transfer and quality improvement
Technology creep or failure to keep up with pace of innovation	(7) Single entry/exit point for innovation proposals	Application process open to all stakeholders with explicit timelines
	(8) Integration of innovation and healthcare delivery	Integration of future testing; private/public sector partnerships
Inequitable or inefficient care	(9) Evaluative function	Consistent and adherent to key health technology assessment (HTA) principles such as timeliness and transparency
	(10) Service model	System-wide care coordination and service delivery planning
Legal liability, low care quality	(11) Regulation	System-wide analytic standards and regulation that addresses human resource qualifications and training, documentation of records, quality control processes, and proficiency testing
	(12) Data privacy and security	System-wide privacy standards

* labeled ‘informatics’ in previous report.

**Table 2:** State of readiness for genome-based testing in Canada. Strengths and weaknesses of individual provinces.

Need for Improvement	Provinces Needing Improvement	Examples	Potential Actions
Broader stakeholder engagement in planning	All provinces	The UK NHS Accelerated Access Collaborative [11]	Collaborate with provincial life science organizations and patient organizations to create ongoing advice and a forum for commercial partnership
Resource planning	MB, NB	US Government and Accountability Office workforce analysis [12]	Conduct and publish periodic (1–3 yr) assessments of overall or specific resource implications of growth in testing
Information management	BC, MB, NB, NS, ON, QC, SK	Alberta Precision Laboratories integration through Connect Care	Standardize laboratory equipment or find HER solutions to integrate laboratory information systems
Entry/exit point for innovation	AB, BC, MB, NB, NS, ON, QC, SK	Newfoundland and Labrador open application process	Publish a transparent process for adding new tests
Evaluation function	All provinces	Similar to NL open application process but involving stakeholders like ON OGAC process	Publish clear criteria, timelines and process for evaluation as in NL. Establish separate innovator advisory function or appoint industry representatives to evaluative committees as in Ontario
Awareness and care navigation	AB, BC, MB, NB, NS, QC, SK	As in ON, NL	Publish comprehensive test formularies with instructions for obtaining tests
Financing approach	AB, BC, MB, NB, NS, NL, ON, SK	As in QC through DLIM	Anticipate, plan and allocate future budgets to laboratory functions. Provide additional funding for test development and provide a clear funding formula that covers all resources
Education and training	AB, BC, MB, NB, NS, NL, QC, SK	As in ON through Provincial Genetics Program	Create an overarching provincial strategy for education of care providers and patients. Create resources and programs to support education

## Data Availability

The original contributions presented in this study are included in the article/Supplementary Materials. Further inquiries can be directed to the corresponding author.

## Supplementary Materials

The following supporting information can be downloaded at: https://www.mdpi.com/article/10.3390/curroncol33060334/s1, File S1, Full Report.

## Appendix A. Interview Guide

### Appendix A.1. Background

I have been asked by a consortium of companies (Alexion Pharma Canada Corp. (Mississauga, ON, Canada), Amgen Canada Inc. (Mississauga, ON, Canada), AstraZeneca (Mississauga, ON, Canada), Boehringer Ingelheim (Canada) Ltd./Ltée. (Burlington, ON, Canada), GlaxoSmithKline Inc. (GSK Canada) (Mississauga, ON, Canada), J&J Innovative Medicine Canada (Toronto, ON, Canada), Roche Canada (Mississauga, ON, Canada), and Thermo Fisher Scientific Inc. (Waltham, MA USA)) to investigate what the current and future state of readiness for advanced (genome-based) diagnostic testing in Canada is and might become.

By advanced diagnostic testing we mean molecular testing (DNA testing such as sequencing, PCR and DNA microarray), cytogenetics (chromosome testing such as karyotyping and FISH), and testing for metabolic products (protein testing through immunoassay or immunohistochemistry).

This work is to help all involved in advanced diagnostic testing in Canada to identify what can be done to make sure that Canadian health systems are prepared for the future of testing.

You have been identified as someone who could provide significant value to understanding the present and future of advanced diagnostic systems either nationally and internationally *on behalf of your organization*. As such, we would like to discuss the subject with you by phone for 45 to 60 min in a semi-structured interview.

Your contribution to this report will be acknowledged as a key informant, but there will be no comments specifically attributed to your organization. Notes from the interview will be shared with you after the call to ensure accuracy and to identify any areas of clarification required

### Appendix A.2. Semi-Structured Interview Guide

The interview begins with the interviewer stating the purpose of the interview, the topics that he wants to explore and the depth of response expected

### Appendix A.3. Purpose

Interviewer: The purpose of today’s interview is two-fold:

1. It will help identify current challenges with the uptake and routine delivery of advanced diagnostic testing
2. To explore what conditions are necessary and desirable for creating robust systems of advanced diagnostic testing (either in your region or generally)

Interviewer: I would like to cover a few topics today that will help answer the question concerning how advanced testing is being conducted today and what changes may be necessary to ensure its continued and effective delivery.

In particular I would like to explore your organizational views on what approaches to the introduction and evaluation of tests, their development and validation, and financing of tests are needed as well as human resource and infrastructure required.

In each case, I will try to describe how much feedback is needed. However, I want to encourage you to speak freely in response to each question, even if you feel it doesn’t directly address the question. We will have [time] for discussion.

### Appendix A.4. Questions

1. Do you feel you have established, partially established or not established each of the necessary conditions for readiness?
2. What are the current challenges with the uptake and routine delivery of advanced diagnostic testing?
3. Permission to Use Name, Interviewee demographics
