# Supplementary material for: Readiness to Consider and Adopt Genetic and Genomic Tests in Canada—An Update to the State of Readiness Report
Source: Curr Oncol. 2026 Jun 4;33(6):334. doi: 10.3390/curroncol33060334 (PMC13298034; doi:10.3390/curroncol33060334)
Supplement: Supplementary file 1 [file curroncol-33-00334-s001.zip › curroncol-4260021-File S1.pdf]

Towards the routine use of genome-based testing in Canada's largest regions:

An update to the State of Readiness Progress Report

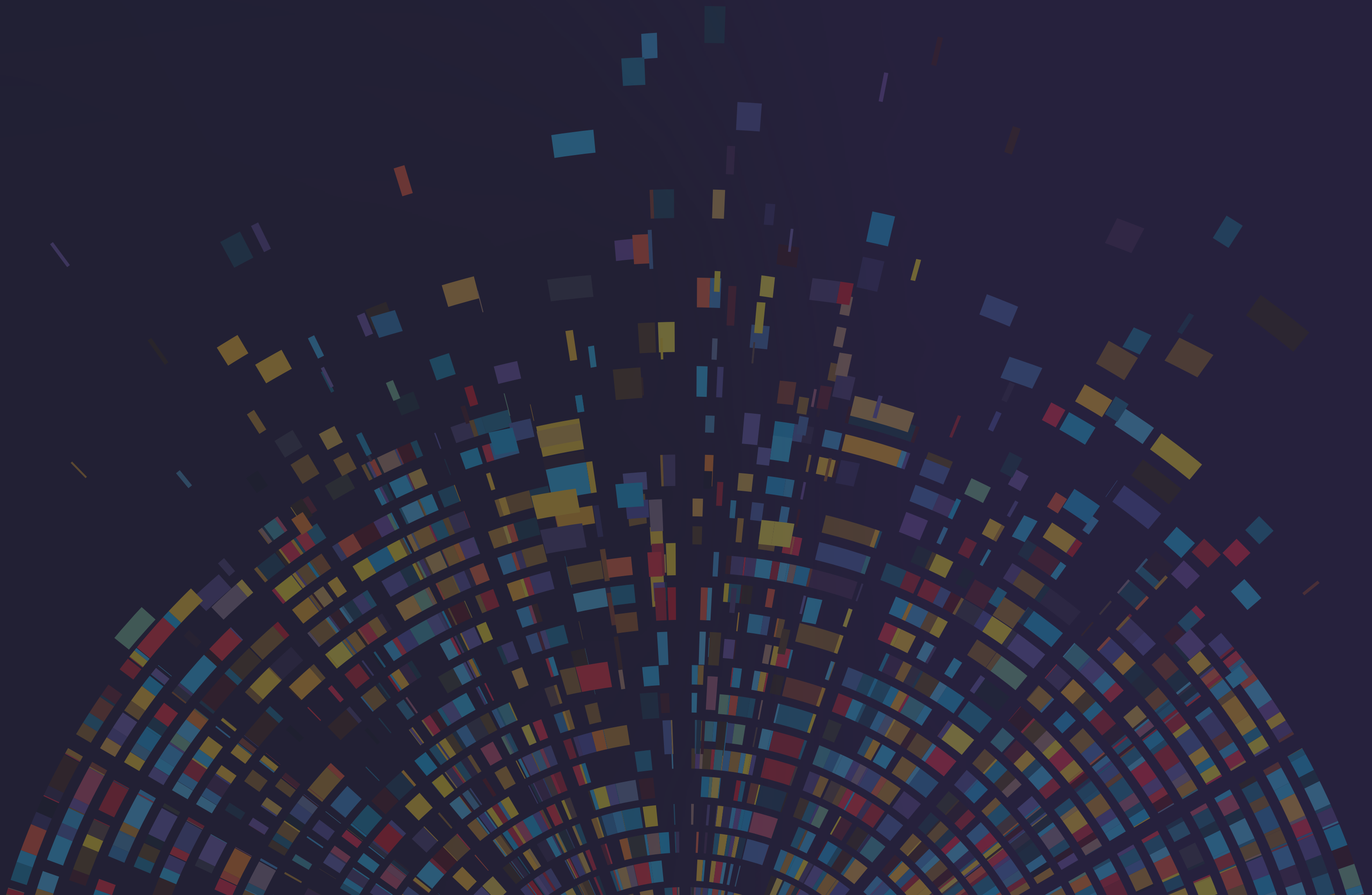

Acknowledgements

Once again, this report would not be possible without the support of a number of people. These include my medical librarian specialist Dagmara Chojecki, as well as Craig Ivany, Michael Mengel, Harriet Feilotter, and Daryl Spinner who provided guidance on how to approach this update. I am indebted to my co-author, Filomena Servidio-Italiano, who also advocates tirelessly through her leadership of CCRAN for patient access to biomarker testing. I am thankful to the number of dedicated laboratory leaders and policy makers who took time to discuss the current state of readiness including Craig Ivany, John Decoteau, Mary Kinloch, Paul Park, Beth Spriggs, Raymond Kim, Kathleen Bell, Aaron Pollett, Harriet Feilotter, Michael Mengel, François Sanschagrin, Tony Reimann, Rodney Ouelette, Pouya Sadeghi Aval, Michael Carter, Lee Anne Boutilier, Darren O'Rielly, and Angela Hyde. While all were helpful in providing organizational perspectives, all of the views and interpretations in the report, including the readiness grades assigned, are my own and do not necessarily represent the official position of any of the individual participants and organizations who participated in this work.

I am also very thankful to Santis Health and Gabriel Oke-Famakinde who provided support for formatting, layout and graphics of the report. Finally, I am especially thankful to those individuals working in the life sciences sector who provided support and direction for this research and were able to convey the experiences of those innovating outside of health systems to improve the health and welfare of Canadians. This includes Aleina Spigelman, Claire Sandor, Kathleen Whelan, Roger Chai, Nisha Chandran, Fiona Choi, Emily Reier, Isabelle Michaud, Luigi Formica, Coral Fairhead, Nadia Yee, Gurvinder Kenth, Lisa Maslanka, and Jeffrey Smith. A very special thanks to Dana Corsen who was instrumental in leading efforts to produce this and the previous report and to Patrick Rooney and Laura Greer from Burson who have supported the engagement of patients and publication of this report.

Citation:

Husereau D, Servidio-Italiano F. Towards the routine use of genome-based testing in Canada's largest regions: An update to the State of Readiness Progress Report. 2026. 66 p. ISBN 978-1-7389568-1-4

Contributions:

D.H conceived and designed all Chapters of the report, conducted the literature review and semi-structured interviews, and analysed content. D.H is responsible for the funding acquisition and led the writing of the original draft. DH and FS-I conceived and designed sections related to patient impact and FS-I led the original draft writing. All authors approved the final version of their respective sections of the report. D.H. is the guarantor of this work. The conclusions of the authors were not contingent on the sponsor's approval or censorship of the manuscript.

Inquiry:

Please direct any inquiries about this report to Don Husereau, Adjunct Professor, School of Epidemiology and Public Health, University of Ottawa: dhuserea@uottawa.ca

Funding:

Research support was provided to Don Husereau's company, 9363980 Canada Inc. by the following companies: Alexion Pharma Canada Corp., Amgen Canada Inc., AstraZeneca Canada, Boehringer Ingelheim (Canada) Ltd./Ltée. Boehringer Ingelheim (Canada) Ltd./Ltée., GlaxoSmithKline Inc. (GSK Canada), J&J Innovative Medicine Canada, Roche Canada, and Thermo Fisher Scientific Inc.

Towards the routine use of genome-based testing in Canada's largest regions:

An update to the State of Readiness Progress Report

Why a progress report?

While it can be difficult to predict whether any particular innovation will be needed, we can be certain innovation in healthcare will be needed. The diffusion (consideration, adoption, and widespread implementation) of valuable innovation into healthcare systems relies on a number of factors. These factors include characteristics of the health system itself (e.g., size, maturity and change leadership functions) and other enabling conditions for managing implementation (e.g., evaluative functions, flexible finance approaches and appropriate infrastructure).

One emerging innovation is genomic medicine – i.e., the use of laboratory-based biomarkers that measure the expression, function and regulation of genes and gene products to aid healthcare decision making. Genomic medicine can improve population health as well as experiences of patients and care providers. It can also reduce health system costs as well as create further opportunities for scientific discovery and future healthcare innovation. Genomic medicine is currently transforming therapeutic areas such as rare diseases and oncology.

Genomic medicine is a complex innovation – it more heavily relies on the timing, expertise, and behaviour of multiple healthcare stakeholders for their efficient and effective delivery. As such, being ready for innovation relies more heavily on health system context and enabling conditions.

The 2026 State of Readiness for the Consideration and Adoption of Genomic Medicine Progress Report is an update to a previously released 2023 "State of Readiness" report—its intent is to examine whether enabling conditions are present across health systems. In doing so it provides an objective assessment of Canada's preparedness and a platform for patients, providers, policymakers and innovators to work toward addressing system gaps. Unlike the previous report, this report focuses on all of Canada. Like the previous report, its focus is on hereditary testing and testing in cancer—infectious disease surveillance is not part of the scope of the report.

What did we find?

Overall, Canada appears to be making progress and is partially ready for a future of genetic and genomic testing in medicine. The report also reveals some additional progress had been made since 2023. All provinces examined in 2023 (British Columbia, Alberta, Ontario, Quebec, Nova Scotia) made some improvement with the most notable improvements in Ontario and Nova Scotia. Prince Edward Island and Canada's Territories refer to other provinces and were not evaluated. Ontario's progress due to establishment of provincial genetics program (PGP) which acts as an established link to the Ontario Pathology and Laboratory program.

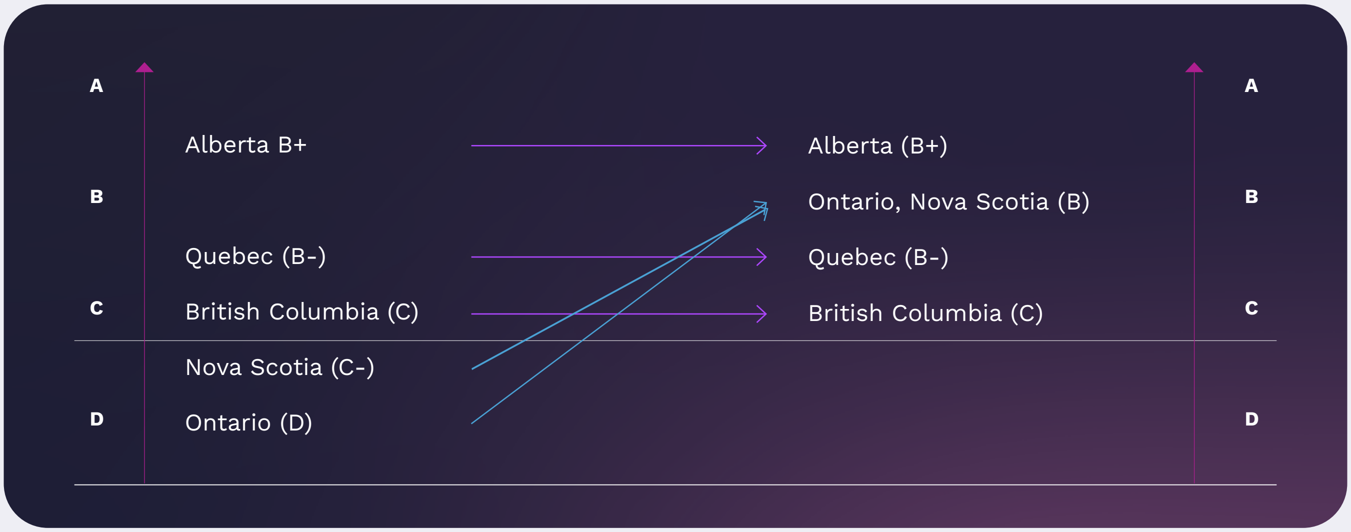

These programs facilitate resource planning and coordination of care for Ontario including the publication of a biomarker test menu. The PGP has also created province-wide standards for education and training. Nova Scotia's progress is largely due to improvements in information linkage and evaluative process for new proposals as well as some improvements in education and regulation (quality) of service delivery. While there have also been incremental improvements in Alberta, British Columbia, and Quebec, none of these changes were enough to warrant change of grade.

All provinces fell between a relatively narrow range (C to B+) of readiness for the consideration and adoption of new tests. There are still gaps in how new testing proposals are considered and evaluated (i.e., HTA approaches), the finance approaches used, linked information systems required, and the need for more systemic educational approaches and resources to support its use, informed by overarching education strategies.

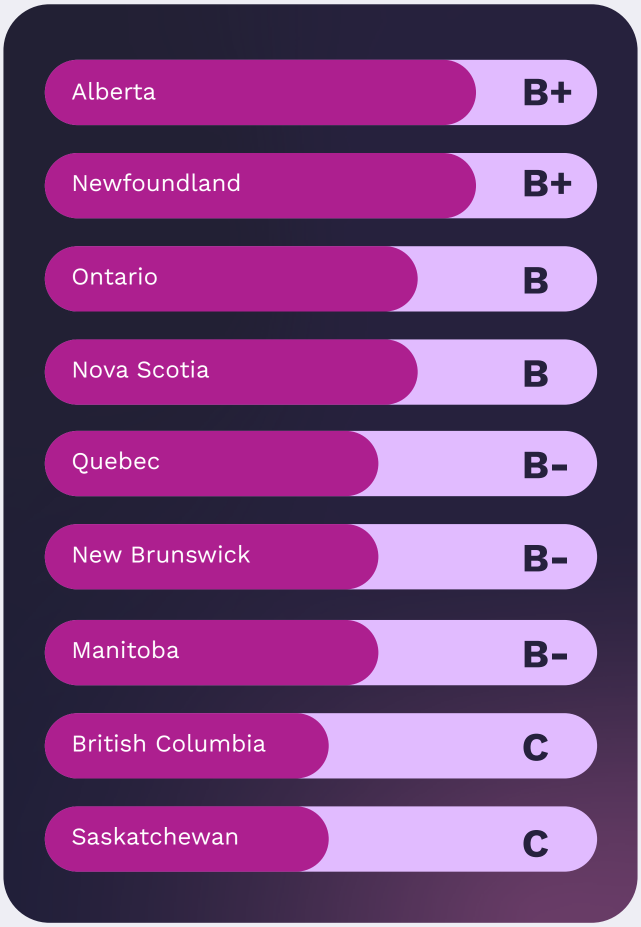

Some key takeaways for feasible changes in each province currently delivering testing are as follows:

Alberta – Formal processes for stakeholder engagement and formally funding test development would create more opportunities to benefit from valuable innovation.

British Columbia – Improving transparency regarding what tests are available and how new tests are introduced coupled with educational standards would improve care delivery.

Manitoba and New Brunswick – Anticipatory planning and providing discretionary funding for the development and delivery of new tests would avoid unnecessary delays for patients.

Newfoundland and Labrador – Repatriation of tests as well as anticipatory planning and discretionary funding for the development and delivery of new tests would avoid unnecessary delays for patients.

Nova Scotia and Ontario – Creating a more transparent system for consideration and evaluation of testing coupled with broader stakeholder engagement would help patients, providers and innovators better understand what is needed and what is valuable.

Quebec – There is a need for Quebec to improve service delivery component through providing better support for navigation and capacity to test patients.

Saskatchewan – There is a need for Saskatchewan to improve linkage of laboratory information systems and provide better support for care navigation and develop more formal processes for testing outside of cancer.

Conditions most established by provinces were service coordination and integration of innovation. Provinces wishing to implement future changes to improving their readiness to consider and adopt advanced testing required for genomic medicine, can consider a number of good practices from other jurisdictions.

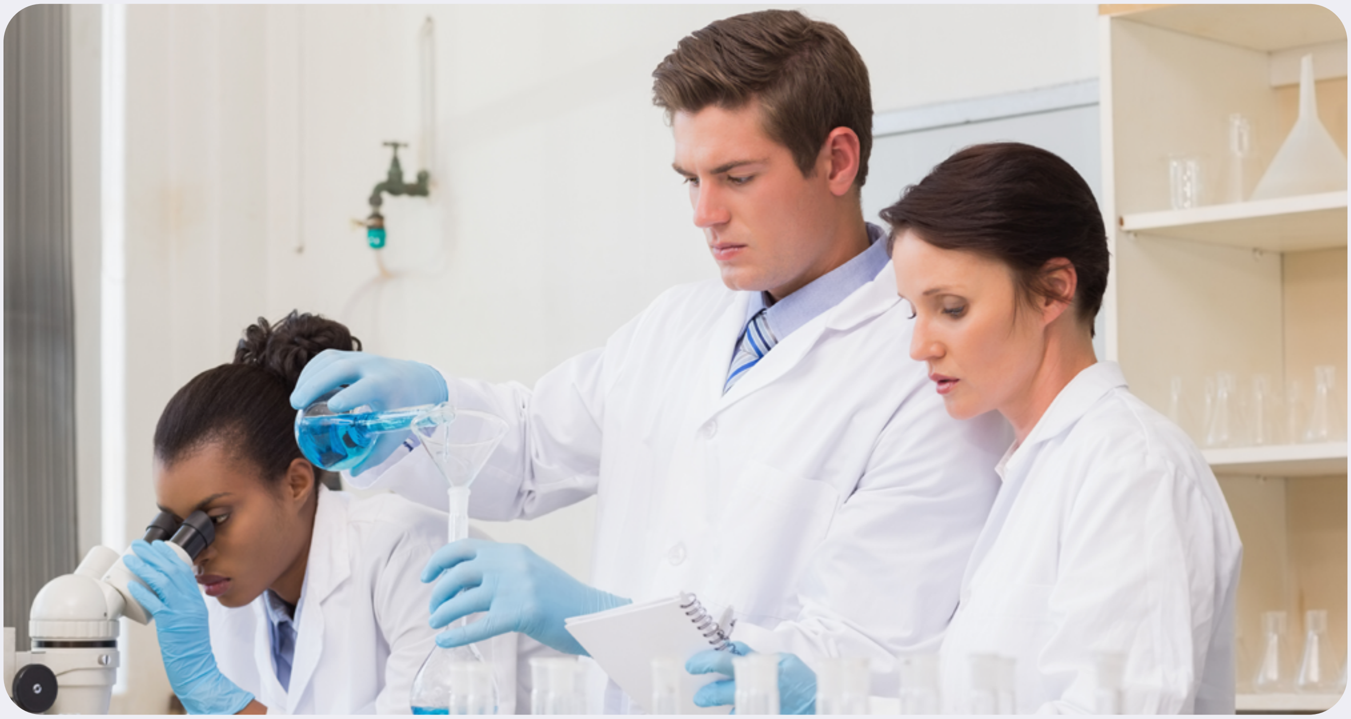

Need for improvement	Provinces needing improvement	Examples	Potential Actions
Broader stakeholder engagement in planning	All provinces	The UK NHS Accelerated Access Collaborative[1]	Collaborate with provincial life science organizations and patient organizations to create ongoing advice and a forum for commercial partnership.
Resource planning	MB, NB	US Government and Accountability Office workforce analysis[2]	Conduct and publish periodic (1-3 yr) assessments of overall or specific resource implications of growth in testing.
Information management	BC, MB, NB, NS, ON, QC, SK	Alberta Precision Laboratories integration through Connect Care	Standardize laboratory equipment or find Electronic Health Record (EHR) solutions to integrate laboratory information systems.
Entry/exit point for innovation	AB, BC, MB, NB, NS, ON, QC, SK	Newfoundland and Labrador open application process	Publish a transparent process for adding new tests.
Evaluation function	All provinces	Similar to NL open application process but involving stakeholders like ON OGAC process	Publish clear criteria, timelines and process for evaluation as in NL. Establish separate innovator advisory function or appoint industry representatives to evaluative committees as in Ontario.
Awareness and care navigation	AB, BC, MB, NB, NS, QC, SK	As in ON, NL	Publish comprehensive test formularies with instructions for obtaining tests.
Financing approach	AB, BC, MB, NB, NS, NL, ON, SK	As in QC through DLIM	Anticipate, plan and allocate future budgets to laboratory functions. Provide additional funding for test development and provide a clear funding formula that covers all resources.
Education and training	AB, BC, MB, NB, NS, NL, QC, SK	As in ON through Provincial Genetics Program	Create an overarching provincial strategy for education of care providers and patients. Create resources and programs to support education.

Looking to the future – implications for Canadian research

While many provinces appear more ready than ever to consider and adopt new testing, some still appear to be less ready to execute (i.e., deliver and monitor) testing in a consistent, high-quality manner due to a wide variety of factors. These factors range from a lack of clarity regarding care pathways and optimized workflows, to poor communication across providers (e.g., from a lack of integrated information systems), to inconsistent knowledge across providers (in part due to a lack of education, awareness and navigational resources), to a lack of specific human resources (e.g., such as laboratory technologists).

While the relative effectiveness and efficiency of delivering new tests is outside of the scope of this report, local health systems and researchers are encouraged to explore local and regional barriers to high quality implementation. In doing so, a full picture of the state of readiness for genomic medicine can be captured.

General remarks

This progress report suggests that Canada's major healthcare regions are moving closer toward a state of readiness for the consideration and adoption of new testing required for genomic medicine, although using different approaches and at different rates.

It highlights the many challenges that health systems face when they are required to quickly respond to a disruptive technology. Even more so, this report highlights the differences in access to care that Canadian may face when they are served by individual health regions with different priorities and health care structures. Simply knowing a technology will promote the health and welfare of Canadians is not enough; we need to have responsive and resilient healthcare systems that are able to quickly shift priorities and be able to recognize and enable value innovation.

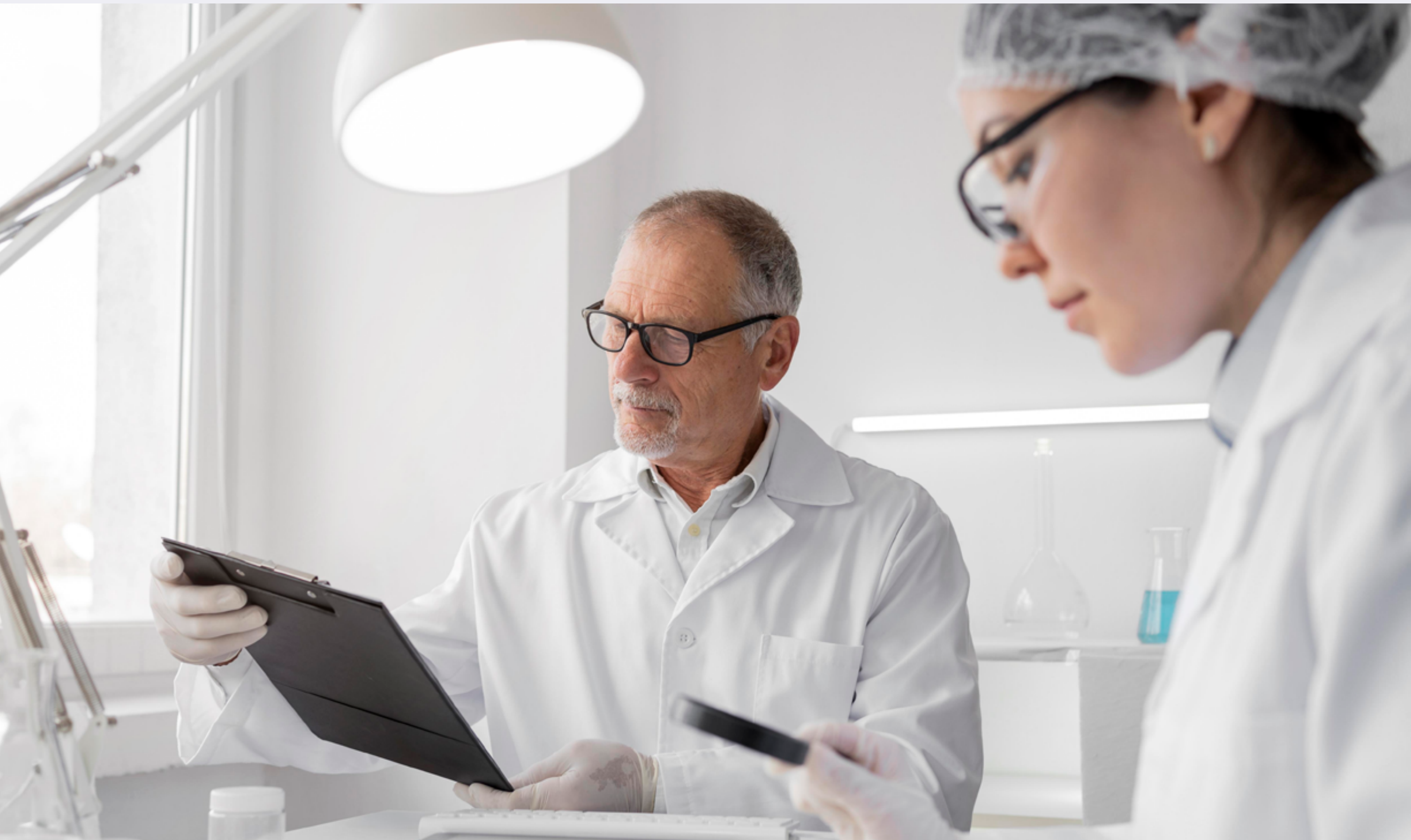

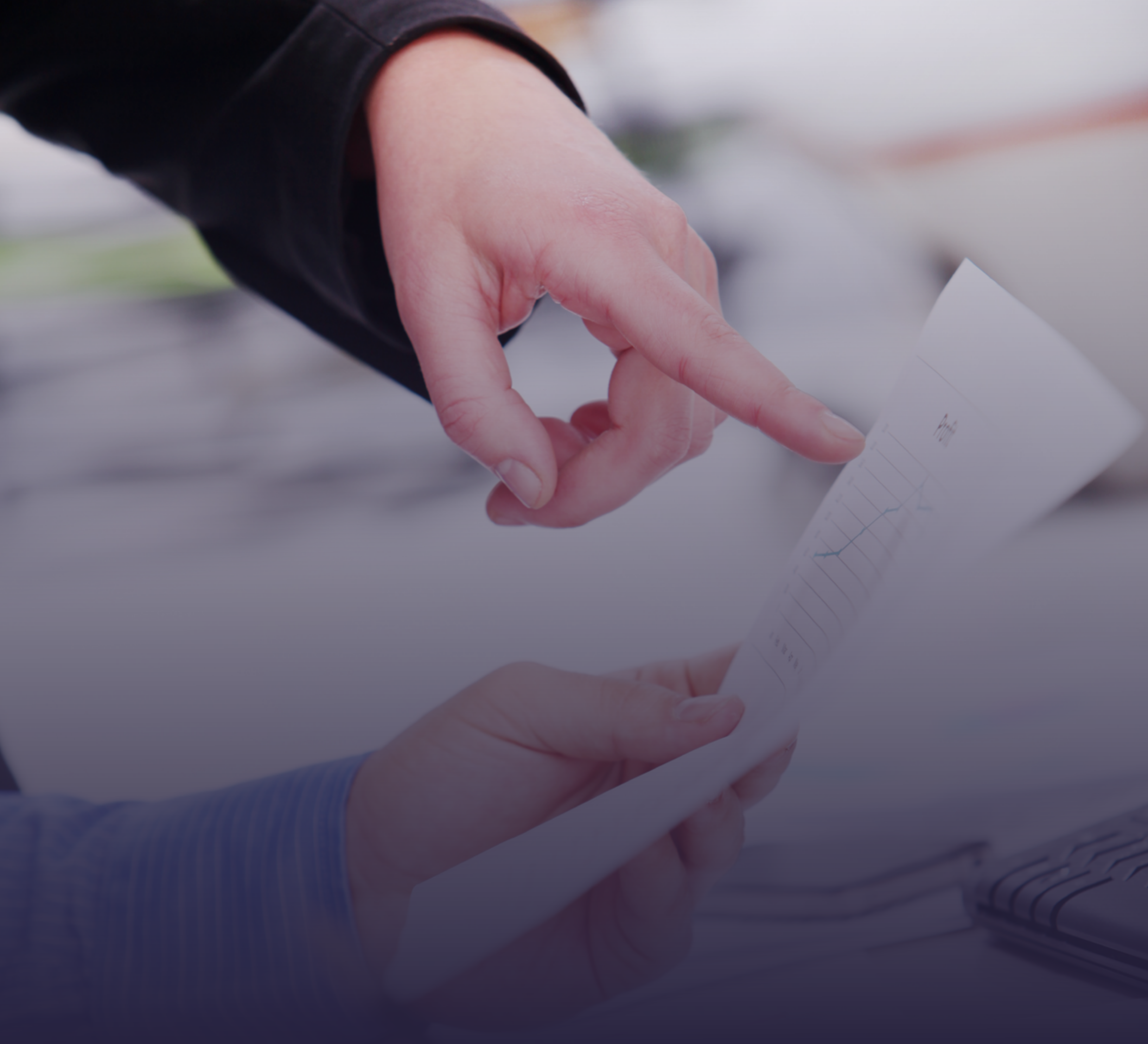

Chapter 1.

Background and purpose of this report

Why a progress report?

The diffusion (consideration, adoption, and widespread implementation) of valuable innovation into healthcare systems relies on a number of factors.[3] These factors include characteristics of the health system itself (e.g., size, maturity and change leadership functions) and other enabling conditions for managing implementation (e.g., evaluative functions, flexible finance approaches and appropriate infrastructure). Reliance on key factors for the diffusion of innovation is particularly true with complex innovations, that more heavily rely on the timing, expertise, and behaviour of multiple healthcare stakeholders for their efficient and effective delivery.[4]

While it can be difficult to predict whether any particular innovation will be needed, we can be certain innovation in healthcare will be needed. Health systems most prepared to benefit from innovation will be able to anticipate, plan, lead and manage the necessary changes required.

One emerging innovation is genomic medicine – i.e., the use of laboratory-based biomarkers that measure the expression, function and regulation of genes and gene products to aid healthcare decision making.[5]

Genomic medicine can improve population health as well as experiences of patients and care providers. It can also reduce health system costs as well as create further opportunities for scientific discovery and future healthcare innovation.[6]

Anticipated growth of genomic medicine has led some health care systems to create the necessary conditions for the consideration, adoption and implementation of new genomic and genetic tests. In the UK, for example, the Department of Health & Social Care committed "£4 billion over a five-year period (2016–21) in digital technology, systems and infrastructure, to provide the health and care system with the digital capability and capacity it needs".[7]

The US has similarly invested over USD $1.5 billion dollars in the National Institutes of Health All of Us research program intended to "accelerate health research and medical breakthroughs, enabling individualized prevention, treatment, and care".[8]

DEFINING TERMS:

Personalized/precision medicine – an approach to tailoring disease prevention and treatment that takes into account differences in people's genes, environments, and lifestyles".

Genomic medicine – the use of laboratory-based biomarkers that measure the expression, function and regulation of genes and gene products to aid healthcare decision making and scientific discovery. Genomic medicine, advanced testing, genetic testing, and genome-based testing may be used interchangeably throughout this report.

The 2023 State of Readiness Progress Report

Recognizing the need for change, a "State of Readiness" Progress Report was released in May 2023 as a way of "objectively communicating the state of readiness of Canadian jurisdictions to appropriately deliver advanced testing services."[9] Its intent was to reflect on Canada's readiness to accelerate the necessary changes required to benefit from genomic medicine.

The approach taken to develop the progress report was to work with international and national policy experts to identify necessary conditions for readiness to consider and adopt new testing. These conditions for readiness were then used to grade Canada's four largest provinces (Ontario, Quebec, British Columbia, Alberta) as well as the largest province in Canada's Atlantic region (Nova Scotia)—representing >85% of the Canadian population.

In 2023, a number of significant gaps in readiness were identified. Provinces were graded for readiness grades ranged from B+ (for Alberta) to D (for Ontario).

Gaps varied by province and included: a lack of informatics/linked data systems; lack of a timely, fair and equitable test review processes; no navigational and educational supports for care providers; timely and adequate financing models for laboratory services; and creating better opportunities for innovation through genomic medicine.

The report was discussed at numerous National meetings attended by health care administrators, life science organizations, and research funding bodies. It had a number of positive impacts and acted as a catalyst for change:

- In British Columbia and Alberta, ~$6M of funding for a new "Healthy Outcomes through Genomic Innovations program" was announced, citing the need to remove system barriers and improve digital infrastructure.
- Alberta released a transparent framework for evaluation of new tests, addressing a weakness in the test review process, making timelines and criteria for evaluation publicly available.
- Ontario created province-wide educational standards and improved its approach to coordinated care delivery.
- Quebec created province wide guidance for selection of gene tests as well as analytic validation standards.
- Nova Scotia improved its test evaluation process, moving to a single intake for new proposals and a more transparent evaluation process.
- The CDA-AMC, funded by Canada's federal, provincial, and territorial governments, drafted a harmonized assessment framework for biomarkers in cancer, citing the progress report and the "need and opportunity for health systems in Canada to better align, coordinate, and harmonize biomarker assessments".

Updating the 2023 Report

The 2026 State of Readiness Progress Report serves the same purpose as the 2023 report to provide an objective assessment of Canada's preparedness and a platform for patients, providers, policymakers and innovators to work toward addressing system gaps. However, there are some noteworthy changes to the 2026 report:

- First and foremost, the original 5 provinces graded in 2023 are re-assessed, allowing the progress toward a state of readiness to be evaluated.
- Secondly, this report addresses Canada's remaining provinces and territories, allowing for a more complete picture of Canada's State of Readiness.
- Lastly, and recognizing the need for change to address areas of growing importance such as rare diseases, the growing field of -omics (i.e., metabolomics, proteomics) that will follow a similar path, and the emergence of new initiatives, the report will highlight the approach to change undertaken by some provinces and needed by others.

How the 2026 State of Readiness Report should be used

While the 2023 State of Readiness Report highlighted the need for change, some patients and providers noted that even provinces "ready" to consider and adopt testing may still not deliver testing in an efficient or equitable way. In part, this was because the focus of 2023 report was high-level readiness conditions that could be addressed by health care system and Ministry leaders. It was never intended to address changes to care delivery that need to occur in regional or local care delivery.

In fact, the quality of, and readiness for effective delivery was not examined at all in 2023. This meant health systems may be ready to consider and make new tests available to patients who may still not benefit from genomic medicine. This could be due to a number of reasons including limited geographic access, human resource shortages, long turnaround times, and inequitable referral practices. Examining these underlying reasons certainly does require further investigation, although may prove to be challenging given Canada's federated health system and different structures of care.

Another aspect of testing out of scope for this report is the increasing use of genomic approaches in infectious disease surveillance and treatment. While an important application of genomic medicine, public health laboratories are typically funded and operationalized outside of regular health care system budgets and planning.

As such, we encourage users of the 2026 report—whether patients, providers, policymakers, or innovators—to use this report to address high-level system barriers to genomic medicine for hereditary conditions and therapeutically, outside of infectious diseases. Readers are, in turn, encouraged to explore local and regional barriers to effective implementation. They should be aware that readiness to consider and adopt innovations in genomic medicine is not the same as readiness to effectively deliver and monitor the impact of genomic medicine.

The need for readiness to consider and adopt testing

As described in the 2023 State of Readiness Report, the need for new testing and test approaches is only expected to grow in coming years. Genomic medicine has the real potential to improve healthcare's quadruple aim, namely patient health outcomes through predicting who may benefit (or not be harmed) from clinical decisions, as well as creating better patient and care provider experiences (e.g., through shortening the diagnostic journey) as well as reducing healthcare costs. These conditions can also lead to better access and more equitable care for patients.

Table 1: How genomic medicine can accomplish the quadruple aim of healthcare.

Aim	Type of test	Description	Outcome
Improving the health of populations	Predictive	Patients responsive to chemotherapy after new diagnosis of advanced ovarian cancer and a BRCA mutation are given olaparib, an oral capsule twice daily for up to 2 years.	At 5 years, 48.3% of patients taking a placebo versus 20.5% taking the drug had progressed disease.<br>An addition 1 in 6 patients taking the drug had continued survival.
	Diagnostic	Infant presenting with congenital disease that was suspected to be life limiting, called Leigh disease.	WES leads to identification of compound heterozygous mutations in SLC19A3, a vitamin B1 transporter protein. Vitamin B1 commenced and infant suffered no further deterioration.
	Research	A 41-year-old male abdominal pain consistent with gallstones has scan revealing cholangiocarcinoma, which spread to liver. Received two courses of therapy with no response.	An FGFR2 mutation was detected. Patient was enrolled in ongoing trial.
Enhancing the patient experience of care	Diagnostic	Infant presenting with unspecified congenital abnormalities.	WES leads to diagnosis of Cohen syndrome, a rare disease, typically diagnosed in late childhood or adolescence once the features manifest, greatly reducing time in the diagnostic odyssey.
Improving the work life of health care clinicians and staff	Diagnostic	Acutely ill, hospitalized, babies less than 1 year of age who received rapid whole-genome sequencing (rWGS).	A 7-day rWGS turnaround meant the baby potentially avoided 10 days in the ICU.
Reducing the per capita cost of health care	Diagnostic	Infant presenting with congenital disease suggestive of monogenic disorders.	An average $AUD 2,182.27 (95%CI: -5,855.02,129.92) avoided.

Beyond the quadruple aim, and unlike many innovations in healthcare, genomic medicine also harbors the real potential to aiding scientific discovery as genetic information can be used to better understand disease or qualify individuals for clinical trial enrollment. Being ready for genomic medicine will create commercial and investment opportunities as well as future-proofing Canada's healthcare workforce (Box 1).

Box 1: Examples of commercial spillovers from investments in genomic medicine.

AbCellera Biologics
A leading biotech startup originating from Genome Canada and Genome BC-funded projects. AbCellera focuses on discovering therapeutic antibodies using cutting-edge technology to scan and analyze immune responses. It has created 100+ high-tech jobs in Canada and attracted significant foreign investment. Notably, it contributed to the world's first COVID-19 clinical trial for a monoclonal antibody treatment.

Biotagenics
Based in Toronto, Biotagenics focuses on clinical microbiomics to improve management and treatment of inflammatory bowel disease. It is a spinout from large-scale Genome Canada-applied research projects, exemplifying commercial success in microbiome diagnostics.

MRM Proteomics
Spun out of the University of Victoria-Genome BC Proteomics Centre, MRM Proteomics develops proteomics technologies and commercial products. The company offers advanced proteomic services and tools for research and clinical applications.

Oxford Nanopore Technologies partnership
Genome Canada's collaboration with Oxford Nanopore supports the development of portable, real-time DNA sequencing platforms. This partnership is significant for advancing precise, accessible genomic research in Canada across health and agriculture sectors.

Platform Genetics
A spinout resulting from Vineland Research and Innovation Centre's work (funded in part by Ontario Genomics and Genome Canada). Platform Genetics harnesses reverse genetics for crop trait development and improvement, advancing Canadian agricultural genomics.

Purpose of this report

Patients, policymakers, care providers, and the public continue to benefit from investments in genomic medicine. Understanding barriers to its broader dissemination in Canada and potential solutions to remedy these is intended to help health systems plan for a state of readiness to consider and adopt new testing. The "State of Readiness" Progress Report has been developed as one way of furthering understanding and objectively communicating the state of readiness of Canadian jurisdictions to appropriately deliver advanced testing services.

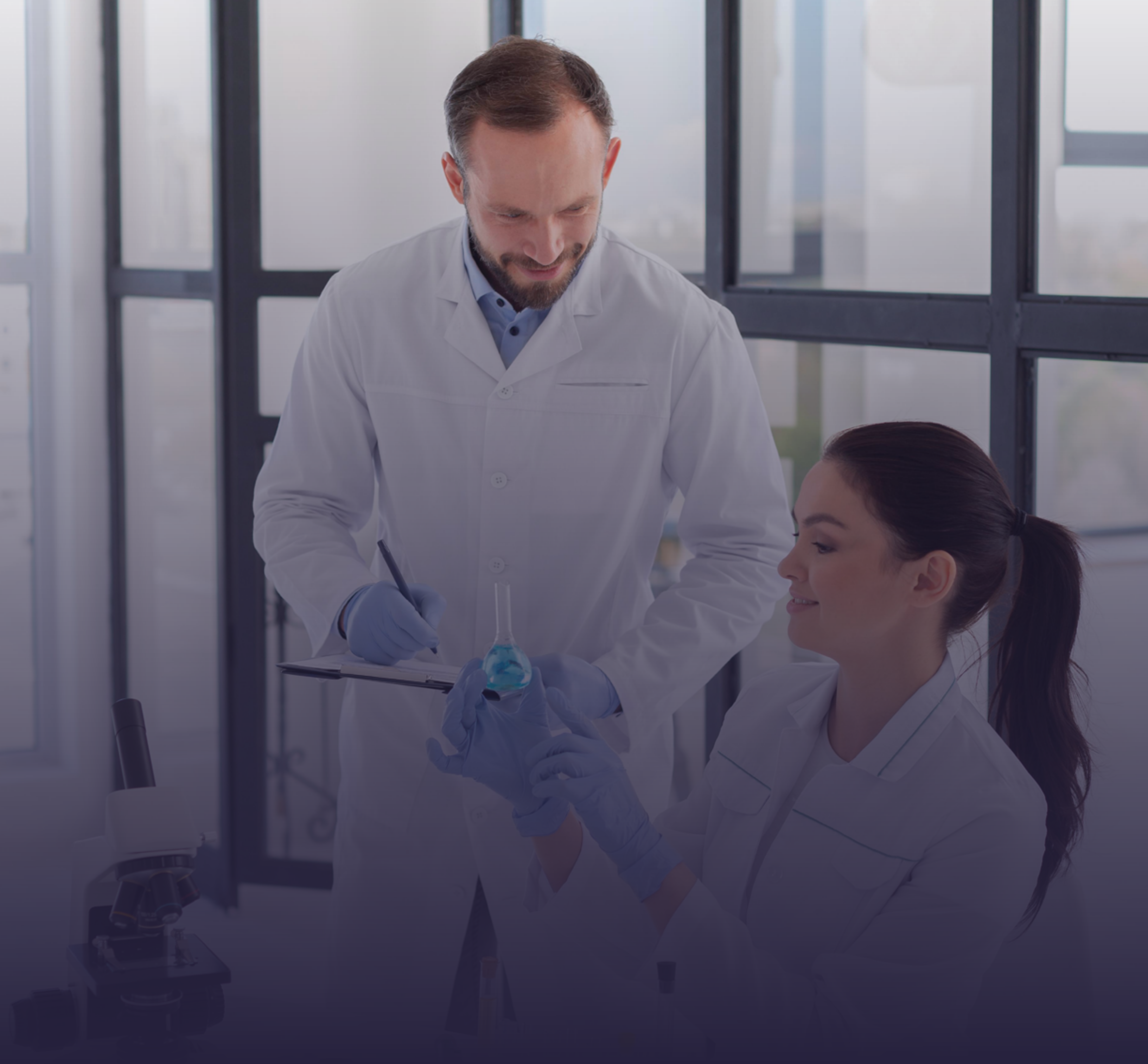

Chapter 2.

Approach taken to the 2026 State of Readiness Progress Report

Information gathering

The 2026 update to the 2023 report follows a similar approach. First a steering committee consisting of sponsor representatives was created to inform the scope and validate findings from the report. Then a mixed methods approach was used that included a narrative literature review and semi-structured interviews of laboratory leaders across all 10 Canadian provinces.

A semi-structured interview guide was developed to facilitate interviews with the stated purpose of the interviews being to: 1) Identify current challenges with the uptake and routine delivery of advanced diagnostic testing; and 2) explore what conditions are necessary and desirable for creating robust systems of advanced diagnostic testing (region-specific for Canadian informants, or generally, for international experts). Key informants, sponsors and sponsor delegates consulted are listed in the Acknowledgements section of the report.

Identifying conditions for readiness

The conditions of good practice developed for the last report were revisited with the principal investigators involved in their development. The authors discussed whether the list of conditions would continue to hold and whether the conditions identified could (or should) be applied to even Canada's smallest regions. The authors decided the same conditions should be used, regardless of the size of a province and for consistency with the 2023 report.

Developing the progress report

Necessary conditions were applied to each of the Canadian provinces to inform the Progress Report. While the territories were also in scope, it was assumed that these act as referral centres to other provinces (and that readiness relies on these other provinces); The grades developed for the progress report were then presented to the steering committee, regional informants and publicly to gather feedback on finalizing the report. None of the sponsors played a role in drafting, revising or approving the content of this research.

Organization of this report

The report is organized as follows:

- Chapter 1 is background information to help readers understand the purpose and scope of this report.
- Chapter 2 describes the approach taken to develop the State of Readiness Progress Report.
- Chapter 3 describes the current and future role of genomic medicine.
- Chapter 4 summarizes necessary conditions and best practices for health systems.
- Chapter 5 describes progress towards readiness for all of Canada's provinces and territories.
- Chapter 6 provides a summary of the potential impact of change and policy implications and concluding remarks.

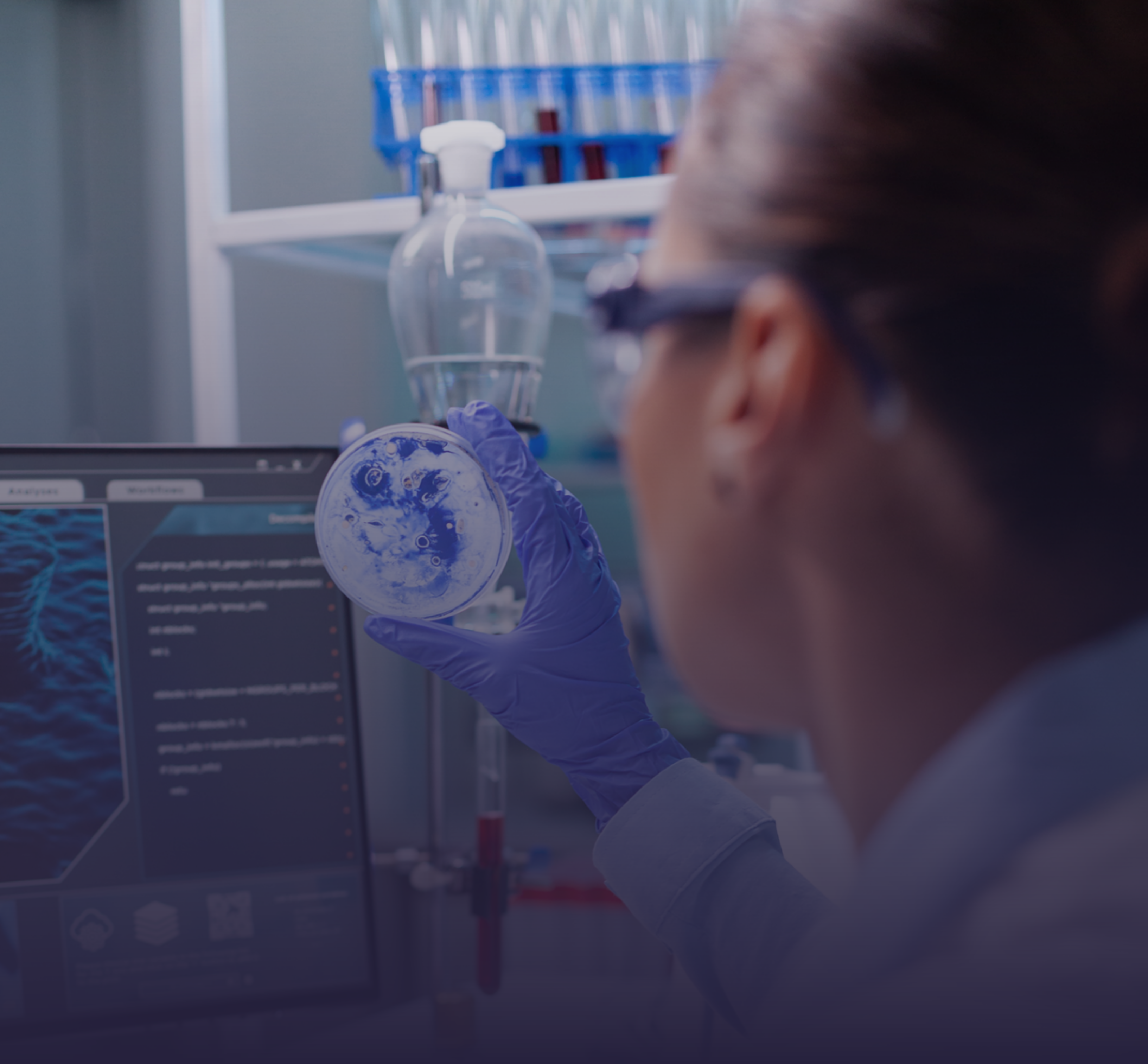

Chapter 3.

The current and future role of genomic medicine

Rare diseases

Rare diseases are those that occur with low frequency and can make diagnosis and treatment challenging. Patients with rare diseases often have a long time to diagnosis, with referrals to multiple specialists and numerous tests before receiving a confirmatory diagnosis – the diagnostic odyssey. The cost of paediatric and adult hospital admissions with rare diseases in the US has been estimated to be nearly half of the US hospitalization costs.[10] A European study found that the economic burden of 43 specific rare diseases far exceeded the cost of cardiovascular disease (€249.3 billion, versus €176.8 billion) in the same regions.[11]

In March of 2023, the Government of Canada announced the launch of the first phase of the National Strategy for Drugs for Rare Disease with funds to be available between April 1, 2024 and March 31, 2027. The agreements generally allocate about 50% of funds for drugs on a common national list, 40% for broader rare disease drug coverage not on the list, and 10% towards diagnostics and screening programs to enable earlier diagnosis of rare diseases.

As an example, in Canada's largest province of Ontario, a funding plan specifically supports enhanced early diagnosis and screening through a $178 million allocation. Key performance indicators set out in the funding agreement include improvements to newborn screening tests for rare diseases and improving access to whole exome and whole genome sequencing to Ontarians.

Who benefits?

Genomic testing for diagnosing rare diseases in children

While genome sequencing an entire population may not be the most efficient use of resources,[12] genome sequencing applied to specific populations may provide tremendous benefits. A particular type of genome sequencing, called exome sequencing (ES), which looks at protein-coding regions of the patient's genome, is now emerging as a more-effective and least costly alternative to traditional testing.

Canadian researchers conducted a simulation of the use of ES upfront versus traditional testing using data collected between 2019 and 2022 following children with suspected genetic disease in the provinces of Alberta and Ontario.[13]

They were able to show that using ES as a frontline test was less costly and more effective in achieving a diagnosis than using it as a second or third-line test (i.e., lines of testing that do not provide conclusive results). Not using ES actually increased costs by roughly $2000 per patient while resulting in 16% fewer (95%CI: 13% to 19%) patients being diagnosed.

Similar findings have been reported in other countries.[14] The use of a rapid exome sequencing test has also been shown to be valuable in other settings. Several studies have examined the impact of rapid ES used with infants hospitalized in critical care settings to aid diagnosis and treatment and have revealed faster times to diagnosis.[15-17]

Genomic testing to screen for rare diseases in newborns

Unlike the use of exome or genome sequencing to diagnose children already showing symptoms of disease, newborn screening programs are typically used to identify asymptomatic individuals at increased risk of or in the early stages of disease.[18] Current newborn screening programs use traditional (i.e., clinical biochemical) testing, rather than genetic testing and position statements from experts suggest that genomic sequencing is not yet justified but may become mainstream as technology develops and more evidence develops.[19-21]

Several studies have been conducted in the area of genetic newborn screening with promising results.[21-26] In one study,[25] in addition to identifying disorders not detectable by current assays (and in infants with no clinical signs of disease), researchers were also able to also detect diseases that may occur in adulthood, as well as carrier status for genetic diseases.

Figure 1: Costs and benefits of using genomic testing as a first line test for infants with suspected monogenic disorders.

					Total cost	Time to diagnosis (years)
ES first test	ES				$2,458	0
No ES	NO ES →	NO ES →	NO ES		$4,347	0.71
ES second test	NO ES →	ES			$3,851	1.25
ES third test	NO ES →	NO ES →	ES		$5,246	1.58
ES fourth test	NO ES →	NO ES →	NO ES →	ES	$6,422	1.81

Adapted from;[13] ES= Exome Sequencing; No ES=all other tests including chromosomal microarray analyses, gene panels, single gene tests, clinical biochemical tests, imaging and other cytogenetic testing.

Genomic testing to diagnose or screen rare diseases in adults

Genomic sequencing can also be used to diagnose disease in symptomatic adults or screen for disease in health adults. This includes screening for hereditary cancers and rare diseases. Studies have indicated that strategies involving genomic sequencing or genetic testing could be highly cost-effective if the right strategies are employed.[27,28]

For example, An Australian simulation study examining the impact of screening all adults in Australia aged 18-25 (and with a 71% uptake) for genes related to hereditary cancer, cystic fibrosis, spinal muscular atrophy, and fragile X syndrome (FXS), and using per-test costs ranging from AUD$200 to $1200 revealed a 31% reduction in cancer deaths and could reduce system costs if the tests were less than AUD$200. At AUD$400, an incremental cost-effectiveness ratio of $4,038 per disability-adjusted life-year was estimated, indicating a highly cost-effective use of resources.

The future role of genomic medicine and rare disease

Taken together, emerging evidence indicates that applications of genomic sequencing to diagnose and find rare diseases will continue to emerge. Strategies involving genomic sequencing may become more effective and cost-effective with lowering test costs or the use of additional low-cost strategies to enhance test effectiveness. This could include the use of AI or genetic information from first-degree relatives to identify patients most at risk for disease prior to the use of tests.

At the Children's Hospital of Eastern Ontario, for example, an AI algorithm called ThinkRare was used to scan 260,000 charts and identify those children most likely to have rare genetic diseases. It was able to identify 8 children most likely to have disease and confirm disease diagnosis in 4 of them.[29] Alberta Health Services has similarly entered a partnership with a European AI company to identify patients with rare diseases.[30]

Oncology

Plasma-based (liquid) biopsy

Liquid biopsy is a technique that analyzes circulating biomarkers shed by tumor cells into bodily fluids.[31] These biomarkers include cell-free nucleic acids such as circulating tumor DNA (ctDNA), circulating tumor cells (CTCs), proteins, and nucleosomes. This method provides detailed information on mutational signatures, tumor mutational burden, specific sequences, and epigenetic modifications.Unlike tissue biopsies, liquid biopsy reflects the heterogeneity of the tumor at a given moment because it captures a mixture of molecules released from diverse tumor subclones.[32]

It offers several advantages over tissue testing, including reduced risk for sampling tumors that are difficult to biopsy, such as pancreatic or prostate cancer, lower costs, and the ability to detect tumor heterogeneity across multiple anatomical sites.[32] Additionally, in colorectal cancer and potentially other cancers, liquid biopsy can be used in adjuvant settings to detect minimal residual disease, helping guide further treatment decisions.[32]

Comprehensive genomic profiling

Genomic testing in Canada uses a combination of different testing approaches including single-gene testing methods through polymerase chain reaction, Sanger sequencing, fluorescence in situ hybridization (FISH), and immunohistochemistry, and multi-gene testing, using massively parallel ("next generation) sequencing hotspot panels.

Comprehensive genomic profiling (CGP) refers to a method by which multiple classes of genomic alterations can be identified in a single assay. It can also be combined with plasma-based comprehensive genomic profiling – or liquid biopsy testing – detect the presence of circulating tumour DNA (ctDNA) in the blood. Liquid biopsy testing may offer some advantages over tissue testing including eliminating the need for a tissue sample, and reducing the time taken to return results to a patient.

Both published assessments[33,34] and those done by independent Canadian health technology assessment (HTA) bodies have concluded that this approach will improve the lives of Canadians at an additional cost and should be considered for patients with advanced-stage NSCLC. These ctDNA-based approaches to care are expected to play a more prominent role in coming years as evidence emerges in breast cancer[35] and colorectal cancer.[36]

Primary cancers of unknown origin

Some (approximately 2% of) patients present with metastases where the original tumour cannot be identified through regular testing. These patients are often provided standard chemotherapy regimens in the hopes of a response when their tumours will more often have a chance of harbouring a clinically actionable biomarker.[37] Emerging evidence demonstrates patients at high risk and with untreated (non-squamous) cancers are better off when offered CGP as part of a diagnostic workup, with 50% of patients delaying disease progression or death by up to 2 months.[38]

Efficient trial enrollment and tumour agnostic drugs

Being ready for the consideration and adoption of new tests will not only benefit patients in mainstream care, but will also have spillover benefits for scientific discovery and learning health systems. Clinical trial recruitment in an era of targeted therapy relies on the identification of patients through testing, but can be inefficient when genetic variants are only present in a small proportion of patients.

Specifically, if a genetic mutation exists only in 1 in 100 patients, then 100 tests must be conducted in order to identify a single patient. For a test that costs $500, this is equivalent to $50,000 in testing costs to find a single patient (or 10$M to find 200). The use of population level CGP , even at 5 times the cost per test, can reduce the costs associated with identifying these patients significantly. Even if tumours present in 1% of tumours, population-level CGP could reduce the costs associated with finding patients by 50% (See Figure 2).

Figure 2: An efficient approach to identifying patients for clinical trials or tumour agnostic genes.

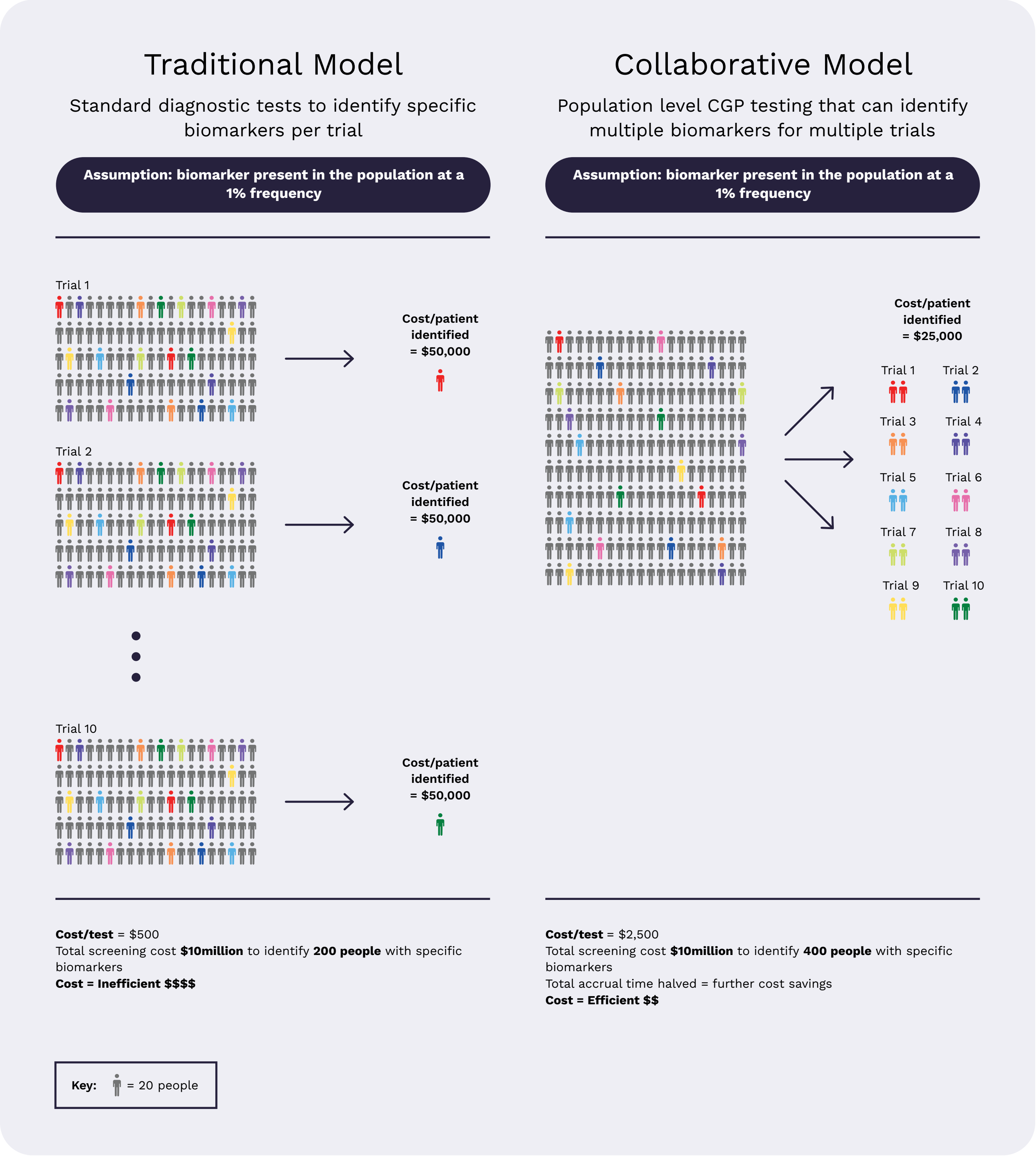

Beyond genomics

While genetic and genomic information will continue to be useful in the detection, diagnosis, prognosis and monitoring of disease, other aspects of the expression of genetic information will emerge to guide therapy.

This includes: transcriptomics, the measure of RNA (as opposed to DNA) transcripts;[39] proteomics, the measure of protein expression in cancer cells; functional impact, the measure of multicellular clusters (organoids), mutation patterns, and tissue function; epigenomics, measure of chemical modifications to DNA and supporting proteins outside of genetic information; metabolomics, the measure of metabolic products; immunomics, that measures immune system activity, and ; microbiomics, the measure of microorganism activity and in the body.

Given these emerging approaches to care, called "multi-omics", genomics has been referred to as only the tip of the iceberg,[40] coupled with machine learning approaches to integrate information and optimize future therapeutic strategies.

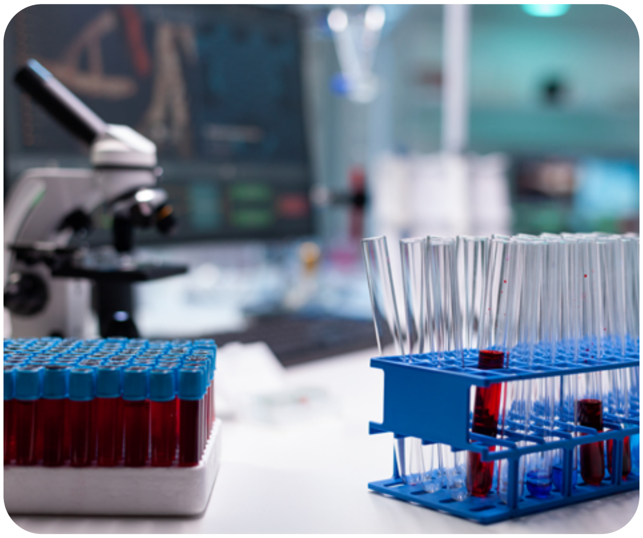

Figure 3: The new era of multi-omics from [40] New era of multiomics. Genomics is the tip of the iceberg. The transcriptome, proteomics, functional impact, epigenetic changes, metabolomics, immunomics, and the microbiome are all tools that will be integrated into future precision medicine therapeutic approaches. Artificial intelligence can help guide interpretation and integration of multi-omics data to optimize therapy.

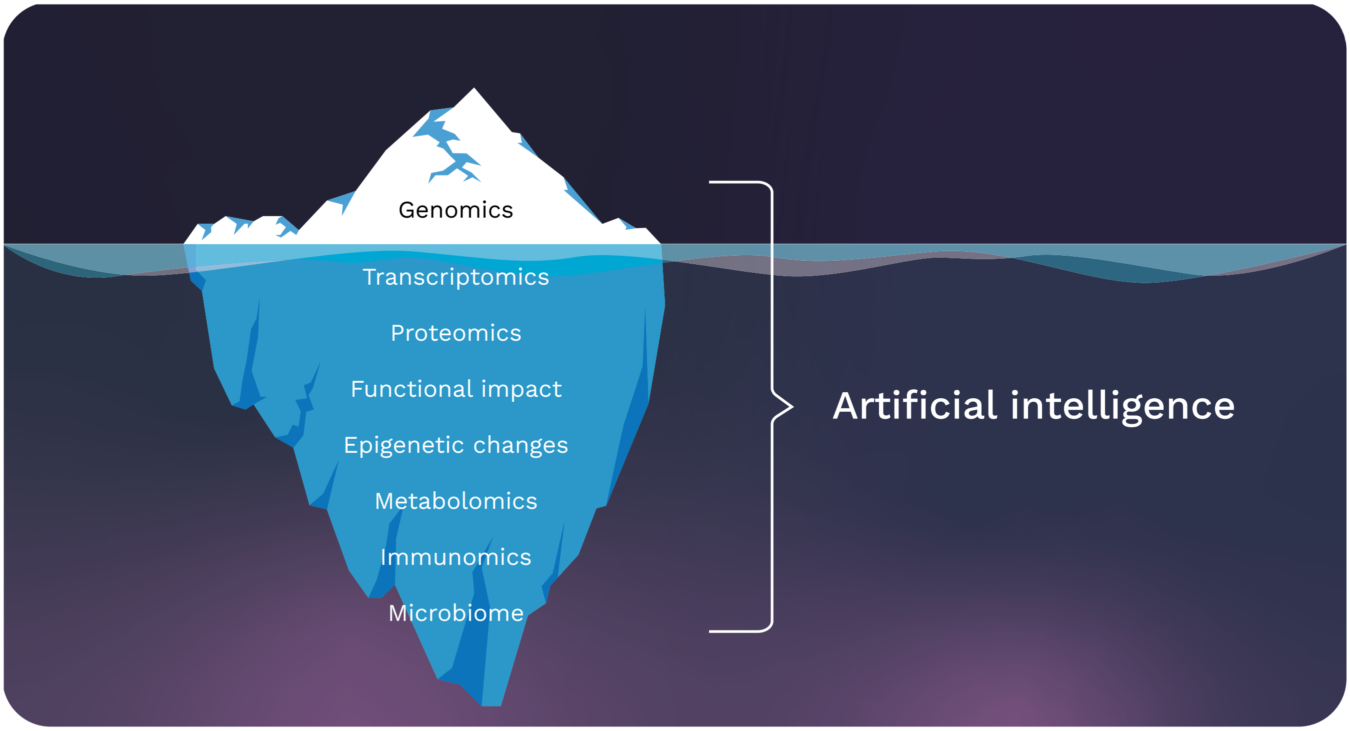

Beyond oncology

A number of therapeutic developments will require health care systems to be ready to consider and adopt testing beyond rare diseases and oncology. For some healthcare systems, this may require fundamental changes in governance and the organization of laboratory services.

Some emerging therapeutic areas that will benefit from genomic approaches include:

- **Rheumatology** – Genomics and multiomics approaches are fundamentally transforming rheumatology by improving diagnostic accuracy, enabling earlier disease detection, and laying the groundwork for individualized treatment and pharmacogenetics in autoimmune and inflammatory diseases.[41,42]
- **Organ transplantation and immunology** – Genomic approaches are used for advanced HLA typing, donor-recipient matching, and predicting rejection risk in solid organ and stem cell transplantation. Integration of genomic data streamlines compatibility scoring and enables early detection of immune-related complications.[43]
- **Neurology and psychiatry disorders** – Genomic medicine is advancing stratification, risk prediction, and diagnosis for diseases such as epilepsy, autism spectrum disorder, schizophrenia, and other neurodevelopmental conditions. Polygenic risk scores are becoming tools for earlier identification and personalized therapy.[44]
- **Chronic kidney disease** – Genomic data, combined with transcriptomics, proteomics, and metabolomics (“multi-omics”), is unraveling the mechanisms underlying chronic kidney disease.
- **Cardiology** – integrating information from genome-wide association studies may allow clinicians to use polygenic risk scores to personalize risk prediction beyond traditional clinical algorithms.[45]

Summing up: the need for consolidated service delivery

In 2026 and beyond, genomic medicine will play an increasingly important role in delivering health care. While it is difficult to predict which applications of genomic medicine will contribute to healthcare system’s quadruple aim, it is certain that the role of genomic medicine will be increasingly important, as the costs of testing lower and evidence of effectiveness emerges.

Health systems ready for the consideration and adoption of testing in rare diseases and oncology will undoubtedly be prepared for the near future. Testing approaches are expected to expand beyond traditional genomic (i.e., DNA- and DNA-product focused) approaches, and beyond rare disease and oncology; therefore, health systems that organize service the planning and execution of service delivery wholistically (i.e., irrespective of disease application) will most likely create the most opportunities for patients to benefit.

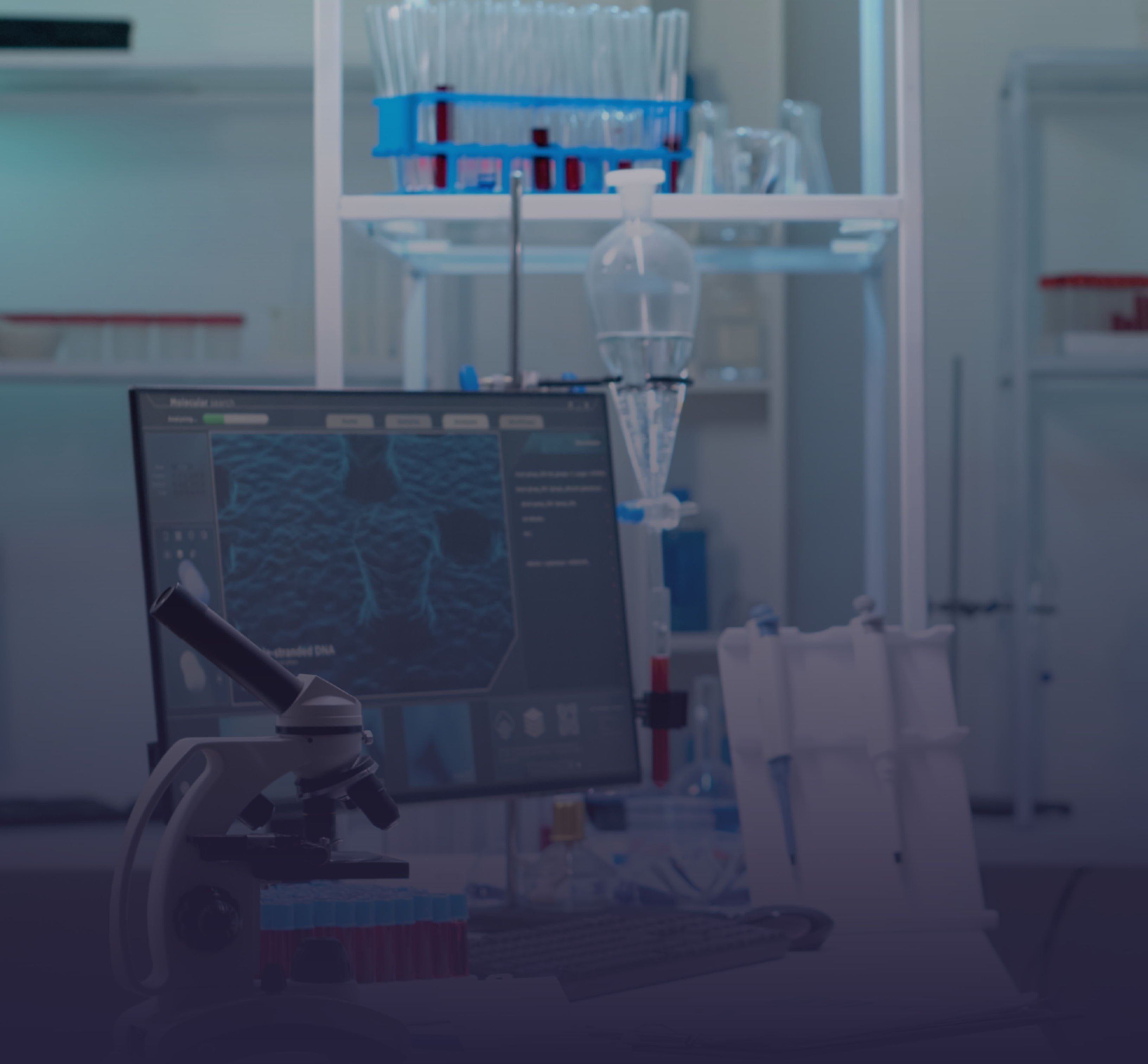

Chapter 4.

Best practices and necessary conditions for the consideration and adopting of new testing

Conditions necessary for the effective and efficient consideration and adoption of new testing are the same as seen in the 2023 report—the open access report is available at the publisher's website (https://www.mdpi.com/2227-9032/10/10/2086) or on PubMed Central's database (https://pmc.ncbi.nlm.nih.gov/articles/PMC9602865/).

Conditions were developed using a mixed-methods approach, combining: a narrative literature review of both commercially published and grey literature; conventional content analysis was used to synthesize findings from the literature; as well as semi-structured interviews.

Eighteen key informant interviews were conducted, each lasting 30–60 minutes, and included a purposive sample of experts from diverse sectors (public, private, health economics, laboratory leadership, administration, and patient representation) and geographies (primarily Canada, with some representation from the US and Europe).

An initial list of conditions was then drafted and circulated among all study authors for feedback and further refinement through a moderated group discussion. The finalized list of enabling conditions was grouped according to the "quadruple aim" of healthcare—improving provider work life, patient/caregiver experience, population health, and cost efficiency—and further elaborated with examples and best practices from various international health systems.

Conditions were then grouped into three domains: "Infrastructure", "Operations" and Healthcare Environment". They are shown in the Figure below.

These conditions are intended to avoid a number of problems associated with the introduction of any innovation, including: inconsistent or inequitable care delivery; unsustainable or low value care delivery (e.g., through duplication of testing); inconsistent testing quality; technology creep; uncoordinated or uninformed care; and delays. A brief rationale of each of the supporting conditions and what constitutes "readiness" is provided here to help with interpretation.

Figure 4: Necessary conditions for readiness to consider and adopt new testing.

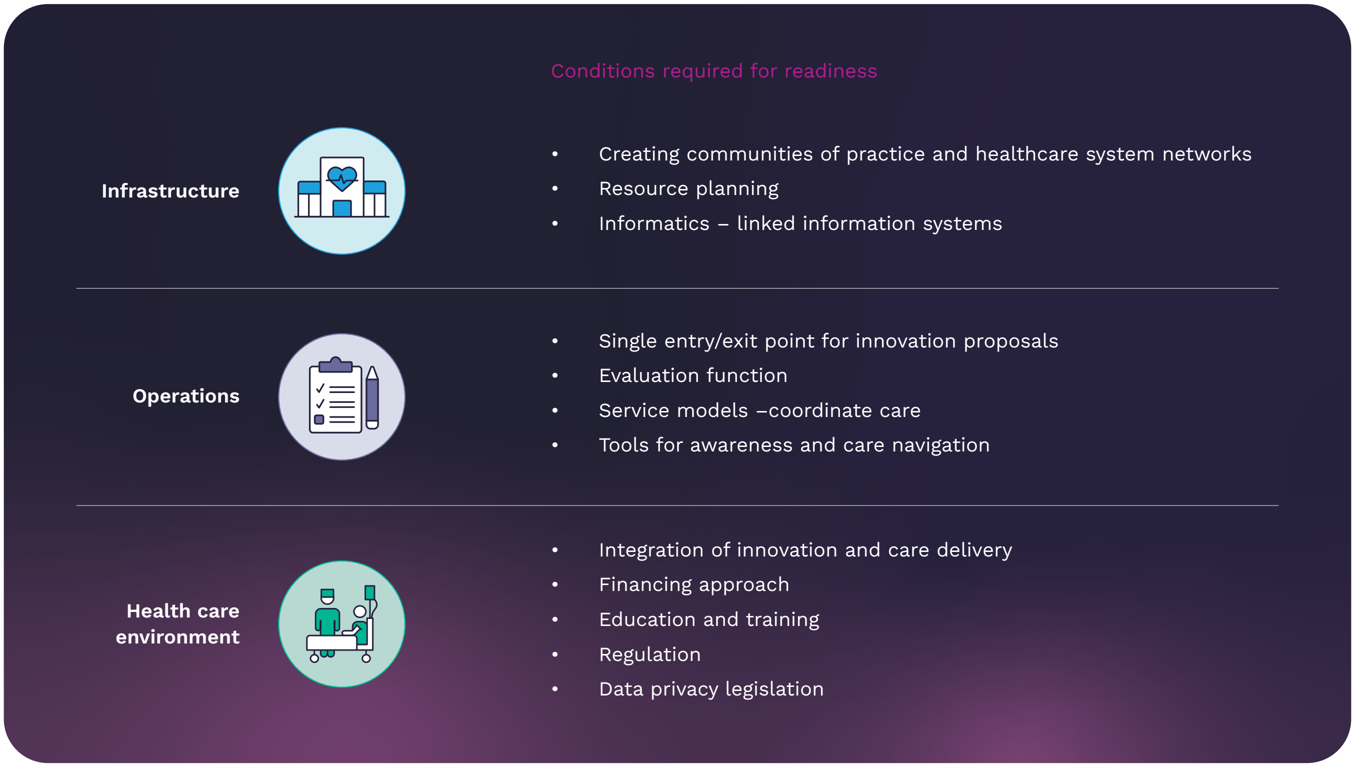

Infrastructure and planning

#1 Creating communities of practice and healthcare system networks

Rationale: Genomic medicine requires coordination and buy-in across a number of specialty services. A well-established network can help with planning and priority setting, as well as the standards and monitoring practices needed to effectively and efficiently deliver equitable care, and other necessary collective judgements that may vary geographically and over time.

What does "ready" look like: Health systems ready to consider and adopt testing will have established formal communities of practice or organizations that engage all stakeholders including patients, administrators, IT professionals, implementation and genome scientists, public and private sector innovators and others (scientists, legal and ethics experts, professional organizations, bioethicists, regulators) as needed.

#2 Resource planning

Rationale: The rapid pace of innovation in this area means delays in adoption will have real consequences for patients. Acquisition of necessary resources (people, facilities, equipment, supplies, and information technology) may take time and requires health systems to anticipate and plan for future changes systematically.

What does "ready" look like: A comprehensive and systematic resource planning and oversight function involves budgeting based on horizon scanning for anticipated resource flows as well as planning to ensure those resources can be acquired.

#3 Information management

Rationale: Beyond the traditional role of processing laboratory information as a tool for sample tracking and communicating results, laboratory information management is essential for test development, interpretation, and clinical decision support functions. It can also lead to timely care and avoid unnecessary duplication of testing.

What does "ready" look like: Linked laboratory information systems that are readily accessible by specialists is a starting point for readiness. Integration of laboratory information systems with electronic health record systems is one means of achieving this although not necessary in all contexts.

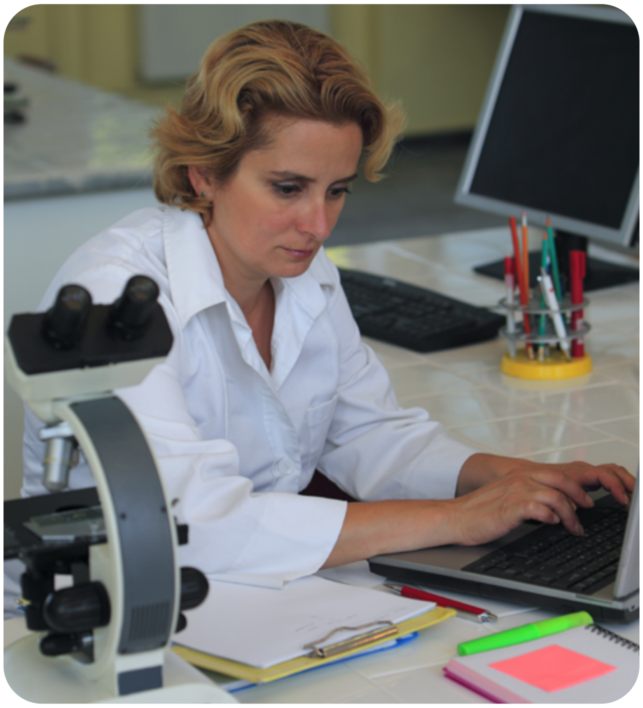

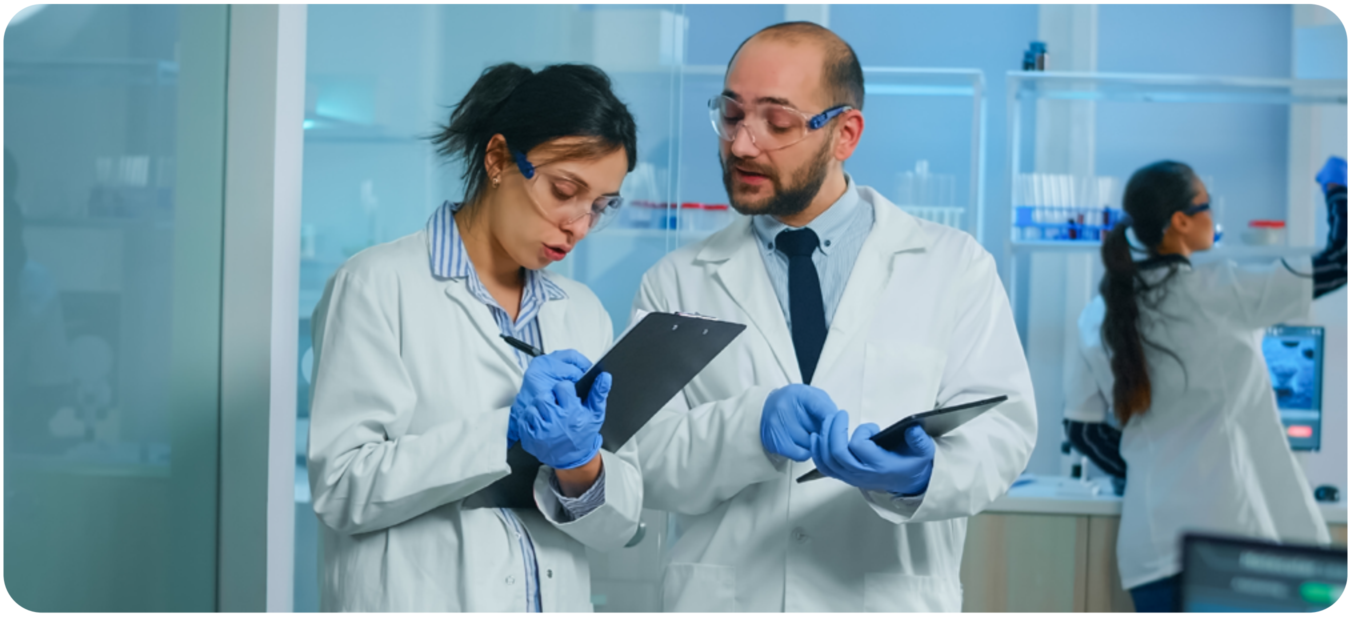

Operations

#4 Entry/exit point for innovation

Rationale: Avoiding technology creep requires managing the entry and exit points for innovation.

What does "ready" look like: A single-entry point for testing that is open to all stakeholders is a minimum requirement for readiness. Not all tests need to go through the same proposal process but the same types of tests (e.g., newborn screening tests vs. companion diagnostics for oncology) should be proposed in a similar manner, for consistency. Reassessment of testing also needs to be part of the process.

#5 Evaluative function

Rationale: Decisions regarding the adoption of new testing and test approaches are complex and require a consistent evaluative function to avoid inconsistent adoption. Timely evaluation is needed so that decisions that impact are not unnecessarily delayed and the process of evaluation should be transparent so that the patients, public and innovators have a good understanding about what is valued and valuable. Engagement with patient and innovator stakeholders may be necessary for improved decision-making.

What does "ready" look like: Good evaluative functions should align with good practices in health technology assessment including being timely and transparent, using best practices in deliberation that allow engagement with key stakeholders including patients and innovators.

#6 Service models

Rationale: The efficiency of testing as well as equitable access to care will largely depend on the model of service delivery employed.

What does "ready" look like: Service models that are highly standardized are required. Larger health systems may require more care coordination to accomplish this.

#7 Awareness and care navigation

Rationale: Effective and efficient use of tests requires patients and care providers to understand what tests are (and are not) available, how to access them.

What does "ready" look like: A comprehensive test list or formulary with information regarding how to access tests and other pertinent information (e.g., turnaround time) is necessary.

#8 Integration of innovation and healthcare delivery functions

Rationale: Readiness in an area of rapid change, coupled with spillover from the research benefits with testing, means healthcare systems will need to consider the managed entry of innovative testing. The time needed for the onboarding of some tests (6 months to a year) means tests need to be developed early so that patients not be harmed from unnecessary delays in adoption.

What does "ready" look like: Translational research programs that facilitate test development prior to adoption are one solution. Another is allowing for the development of both mainstream and future tests; this is also facilitated by massive parallel ("next-generation") sequencing where additional tests can be added to an assay at negligible cost. The reporting of results to specialists at the time of "adoption" can then be implemented rapidly.

#9 Financing approach

Rationale: Traditional financing approaches, such as annual funding based on test volumes, may not take into account the additional human resource costs related to test development and validation, proficiency testing and technologist time required for new tests. Discretionary spending will also reduce delays in providing access.

What does "ready" look like: A nimble and transparent financing approach that considers additional resource requirements for test development and deliver and the ability to commit funds rapidly are necessary conditions for readiness.

#10 Education and training

Rationale: Changes in workflow from new tests "necessitates training at the intersection of continuing professional development, knowledge transfer and quality improvement". Educational strategies should not only consider care providers but patients.

What does "ready" look like: Systematic training or an educational strategy with province-wide standards or guidance.

#11 Regulation

Rationale: Patients will consistently benefit from genomic medicine if there are explicit standards for ensuring quality of testing and analytic validity as well as proficiency testing.

What does "ready" look like: Accreditation standards for labs, standards or processes for creating analytic validity standards, and standards for proficiency testing.

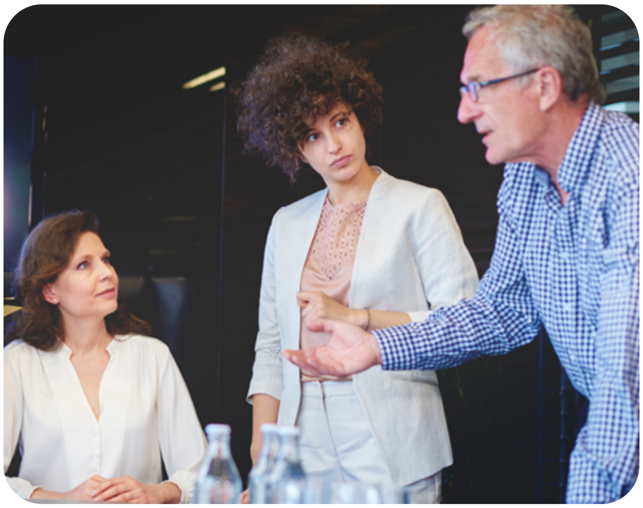

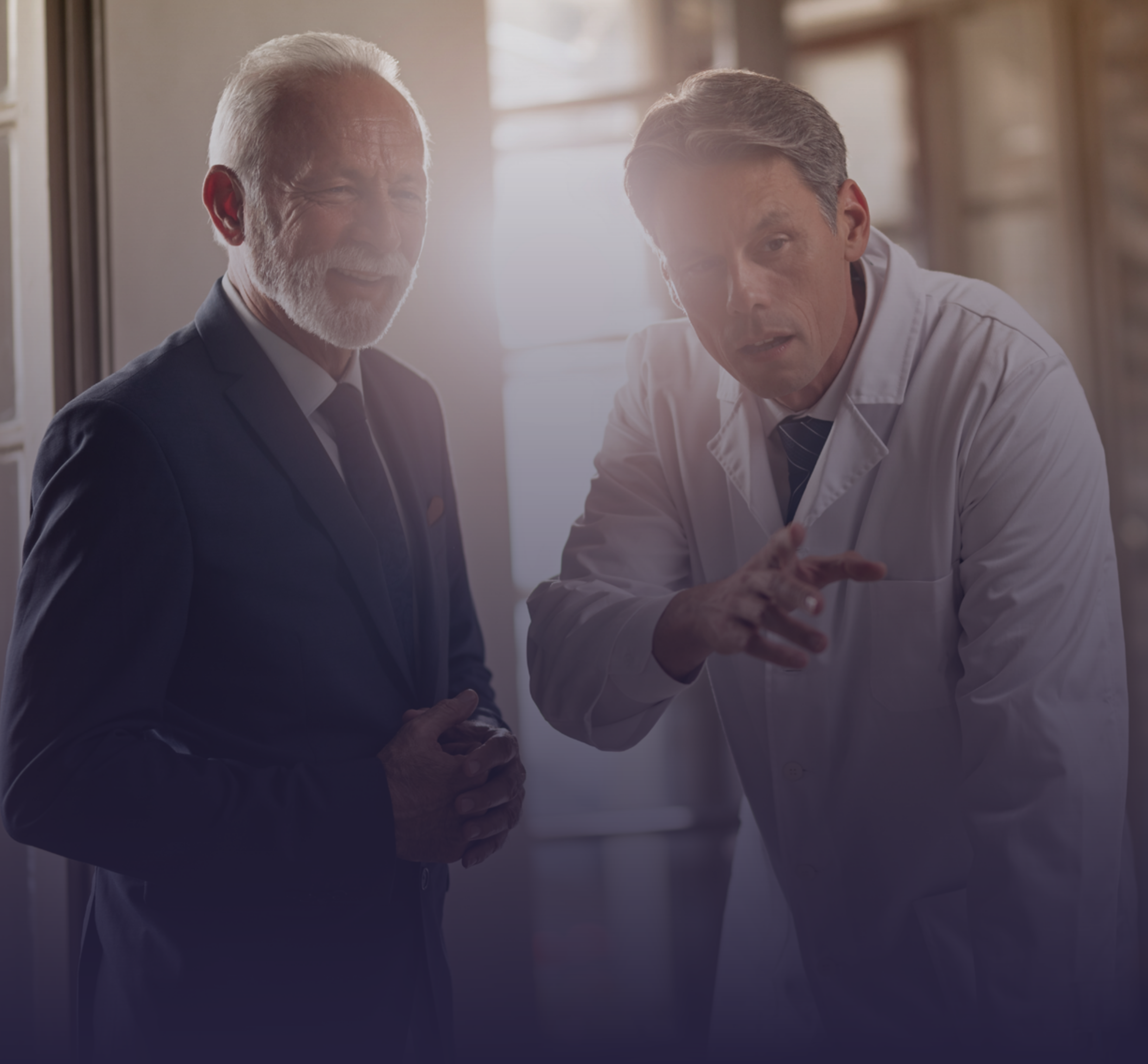

Chapter 5.

State of Readiness for genomic medicine in Canada

This new look at the State of Readiness for genomic medicine reveals some additional progress had been made since 2023. All provinces examined in 2023 (British Columbia, Alberta, Ontario, Quebec, Nova Scotia) made some improvement with the most notable improvements in Ontario and Nova Scotia (Figure 5).

Ontario's progress due to establishment of provincial genetics program (PGP) which acts as an established link to the Ontario Pathology and Laboratory Medicine program. These programs facilitate resource planning and coordination of care for Ontario including the publication of a biomarker test menu. The PGP has also created province-wide standards for education and training.

Nova Scotia's progress is largely due to improvements in information linkage and evaluative process for new proposals as well as some improvements in education and regulation (quality) of service delivery.

While there have also been incremental improvements in Alberta, British Columbia, and Quebec, none of these changes were enough to warrant change of grade.

Nonetheless there are some key gaps that remain. In most provinces, more work needs to be done in creating HTA processes that conform with good practices (i.e., timely, responsive, transparent). Finance approaches still largely ignore need for discretionary spending and test development, creating the potential for delayed introduction.

There are still challenges with information management although most provinces have large (but slow moving) plans to improve.
There is also generally a lack of educational standards and strategies in most provinces. A breakdown by province is provided in Figure 3.

Figure 5: Progress in provinces evaluated in the 2023 State of Readiness Report.

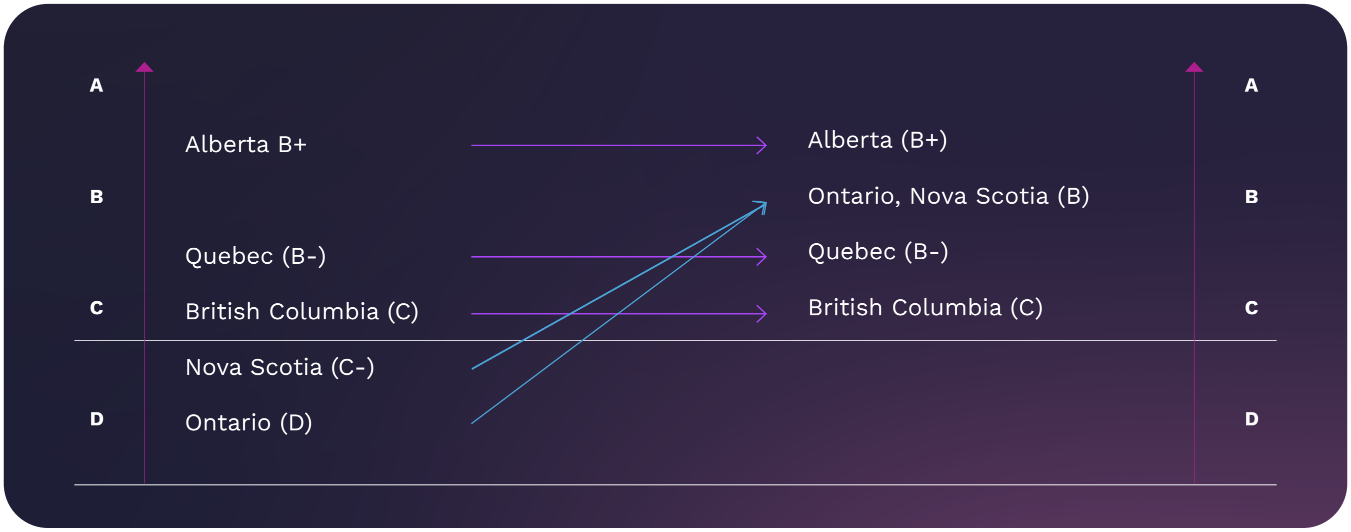

Alberta

Alberta is Canada's fourth largest province by size and by population (approx. 5.0 million). Responsibility for testing is provided by a single organization, Alberta Precision Laboratories (APL), which is operated by Alberta's single health authority, Alberta Health Services (AHS). Highly specialized genomic/genetic testing is delivered as a provincial program within APL directly reporting to executive medical and operational leadership. APL develops a provincial test menu and delegates testing to hospitals within Alberta's largest centres (Edmonton and Calgary) depending on program of care, including the University of Alberta, Alberta Children's Hospital, Stollery Children's Hospital, and University of Calgary, Foothills Medical Centre. Testing is also referred by APL to out-of-province providers for rarer conditions.

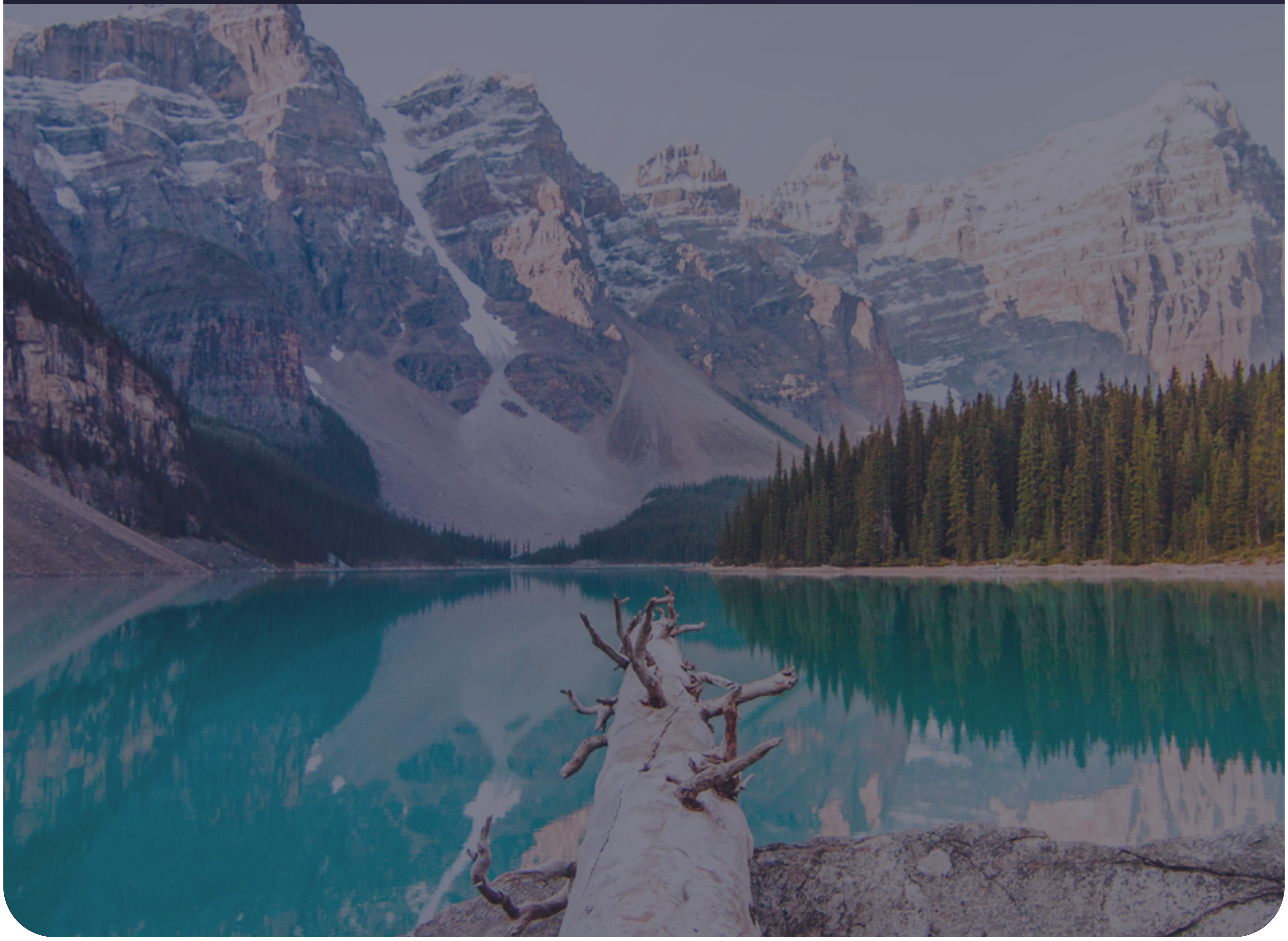

Alberta's strengths

- ***Infrastructure for coordination and planning*** – Alberta's single service organization, Alberta Precision Laboratories, provides oversight and resource planning, intraregional care coordination, and acts as a single point of entry for proposals for new innovation, including proposals from innovators outside the health system.
- ***Advanced informatics*** – Integration of laboratory information systems across the province with electronic health record data is fully established.
- ***Innovative testing*** – Alberta hosts dedicated translational research programs for public and private sector innovators and incorporates investigational testing into mainstream healthcare.

Alberta's weaknesses

- ***Creating opportunities for innovation*** – the timelines for managing and deciding test priorities are not publicly available. There are also no structured opportunities for exchange with innovators from outside of the healthcare system. This makes it difficult for innovators to plan their own development portfolios, or provide useful information that might benefit healthcare planning.
- ***Finance approach*** – Alberta's reliance on research funding to develop new tests means testing health system priorities are influenced by who is paying, rather than societal need, equity, or efficiency.
- ***Support for care providers*** – Alberta may benefit from a more systematic approach to training of care providers as well as provide a comprehensive list of tests available.

Its state of readiness to consider and adopt new tests has earned **Alberta a grade of B+**

Takeaway: Alberta has many of the necessary conditions and is currently leading Canada in its state of readiness for genomic medicine. More formal processes for stakeholder engagement and formally funding test development would create more opportunities to benefit from valuable innovation.

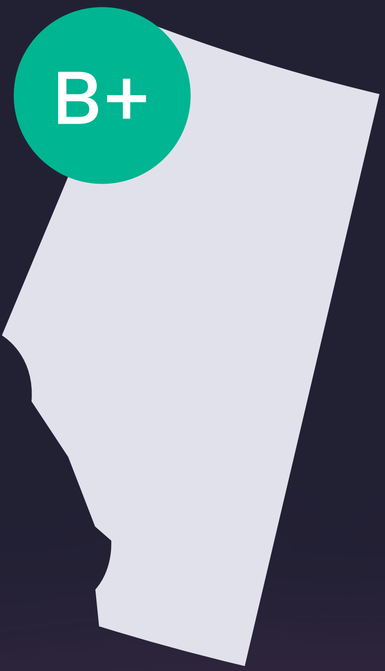

Table 2: Alberta's progress report - readiness to consider and adopt new testing.

Condition	Assessment	Description
Creating communities of practice and healthcare system networks	Some aspects established with others in need for improvement	• APL/AHS responsible for coordination<br>• No standardized process for broader stakeholder engagement
Resource planning	Established	• Systematic oversight for resource planning through the APL
Information management*	Established	• Laboratory and clinical data integrated
Entry/exit point for innovation	Some aspects established with others in need for improvement	• Single point of entry, open application process<br>• No explicit timelines
Evaluative function*	Some aspects established with others in need for improvement	• Transparent process of evaluation<br>• No broad stakeholder engagement
Service models	Established	• APL/AHS responsible for coordination
Awareness and care navigation	Partially established	• Test directory<br>• Not all tests (e.g., oncology) listed
Integration of Innovation and healthcare delivery	Established	• Investigational testing funded and translational research program
Financing approach	Some aspects established with others in need for improvement	• Discretionary spending<br>• No funding for test development
Education and training	Partially established	• Some aspects of training laboratory human resources have been developed
Regulation	Established	• APL is externally accredited with councils for creating analytic standards

*Has improved since 2023

British Columbia

Canada's second largest province by size and third largest by population (approx. 5 million) has leveraged its single health authority dedicated to highly specialized services (the Provincial Health Services Authority, PHSA) to coordinate the delivery of genetic testing. Highly specialized testing is delegated to larger teaching hospitals (Vancouver General Hospital; St. Paul's Hospital; Royal Columbian Hospital; BC Children's Hospital) depending on type of test or therapeutic program. Testing is also referred to out-of-province providers for rarer conditions. The BC Provincial Laboratory Medicine Services (PLMS, formerly the BC Agency for Pathology and Laboratory Medicine, BCAPLM) is the Provincial Program under the PHSA which is responsible for the administration and provision of insured laboratory benefits to British Columbians.

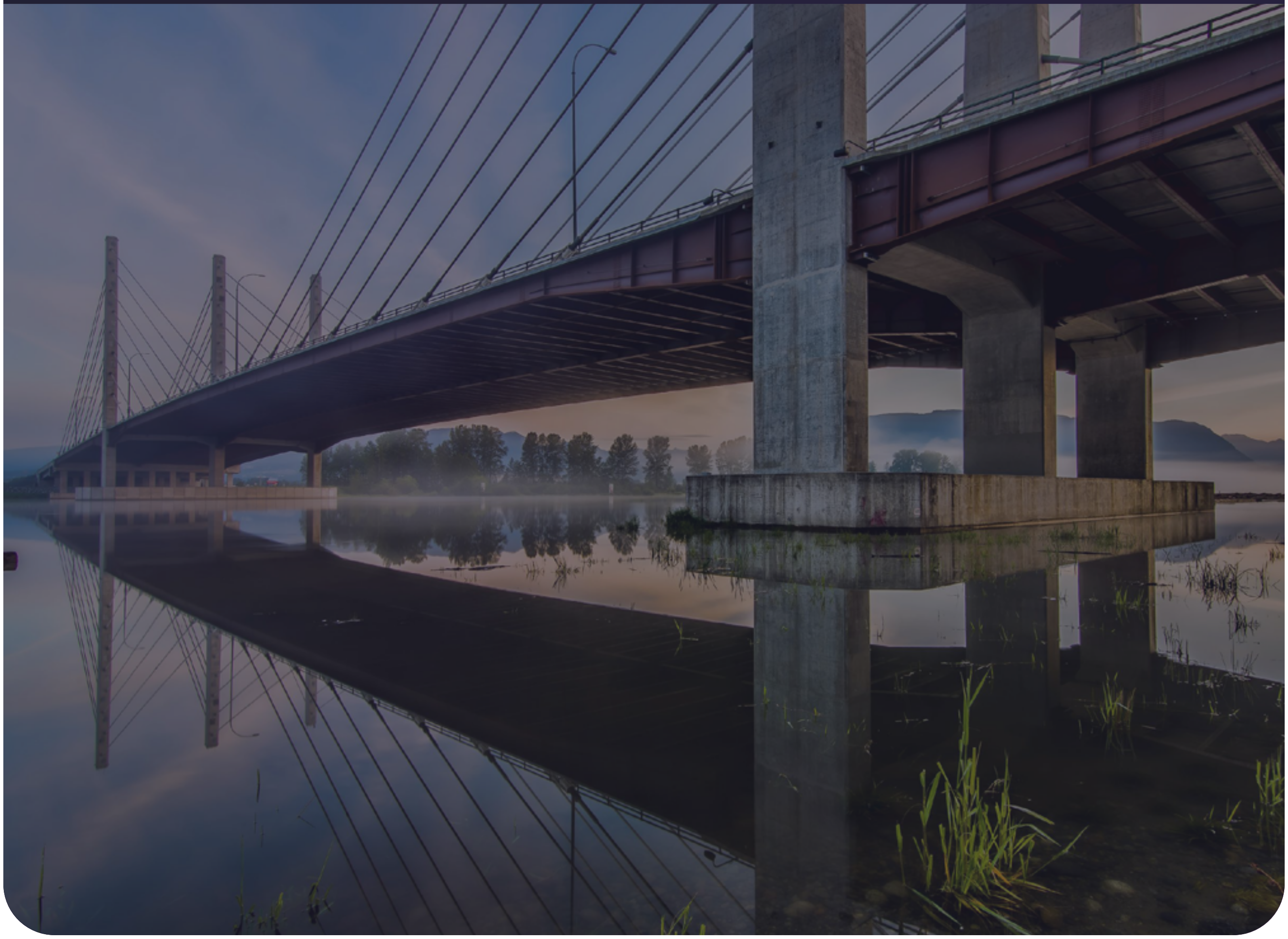

British Columbia's strengths

- ***Infrastructure for coordination and planning*** – British Columbia's single service organization, British Columbia (BC) Provincial Laboratory Medicine Services (PLMS), supports resource planning and has been able to create a community of practice to facilitate service coordination. The PLMS also acts as a single point of entry for proposals for new innovation.
- ***Forward looking*** – BC has embraced investigational testing and translational research through their Genome Science Centre. They have been working toward fundamental changes in information management as well as how tests are considered and evaluated since the 2023 Progress Report.

British Columbia's weaknesses

- ***Creating opportunities for innovation*** – BC lacks any formal mechanism for broader stakeholder engagement with patients and industry in regards to testing.
- ***Finance approach*** – BC's reliance on research funding to develop new tests means testing health system priorities are dictated by who is paying, rather than societal need, equity, or efficiency.
- ***Support for care providers*** – Care providers in BC would benefit from better transparency regarding the availability of test as well as educational resources to optimize their use.

Its state of readiness to consider and adopt new tests has earned **British Columbia a grade of C**

Takeaway: British Columbia continues to take necessary steps to advance its system readiness for genomic medicine. Some challenges relate to its regionalized health system structure and lack of broader engagement. Improving transparency regarding what tests are available and how new tests are introduced coupled with educational standards would improve care delivery.

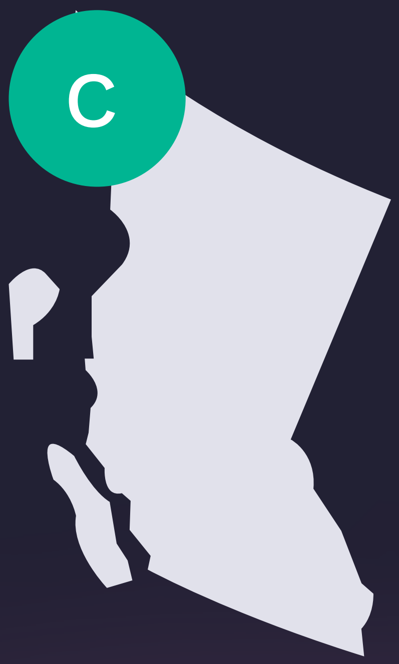

Table 3: British Columbia's progress report - readiness to consider and adopt new testing.

Condition	Assessment	Description
Creating communities of practice and healthcare system networks	Some aspects established with others in need for improvement	• PHSA/PLMS responsible for intraregional coordination<br>• No standardized process for broader stakeholder engagement
Resource planning	Established	• Systematic oversight for resource planning through the PLMS
Information management*	Some aspects established with others partially established	• Integration of genetic laboratory information systems underway
Entry/exit point for innovation	Some aspects established with others in need for improvement	• Single point of entry, explicit timelines<br>• New evaluation framework under development<br>• Closed application process with no opportunity for reassessment
Evaluative function	Some aspects established with others in need for improvement, and others in need for improvement	• Process of evaluation currently being revisited<br>• No broad stakeholder engagement
Service models	Some aspects established with others partially established	• PHSA/PLMS facilitates some service coordination<br>• Some informal coordination regionally
Awareness and care navigation*	Partially established	• Navigation facilitated by nurse care coordinators<br>• No test directory
Integration of Innovation and healthcare delivery	Established	• Investigational testing funded<br>• Translational research program through Genome Science Centre
Financing approach	Some aspects established with others in need for improvement	• Some discretionary spending through life support fund<br>• Non-flexible funding formula / No funding for test development
Education and training	Need for improvement	• No province-wide standards for education and training
Regulation	Some aspects established with others partially established	• Province-wide accreditation standards<br>• No province-wide analytic standards

*Has improved since 2023

Manitoba

Canada's sixth largest province by size and fifth largest by population (approx. 1.5 million) uses its program dedicated to coordinating laboratory and diagnostic imaging services, Shared Health, to coordinate the delivery of genetic testing. Testing is conducted at the Health Sciences Centre Winnipeg. Shared Health Manitoba has developed a testing menu although not all genetic biomarkers are listed. Testing is also referred by Shared Health to out-of-province providers for rarer conditions.

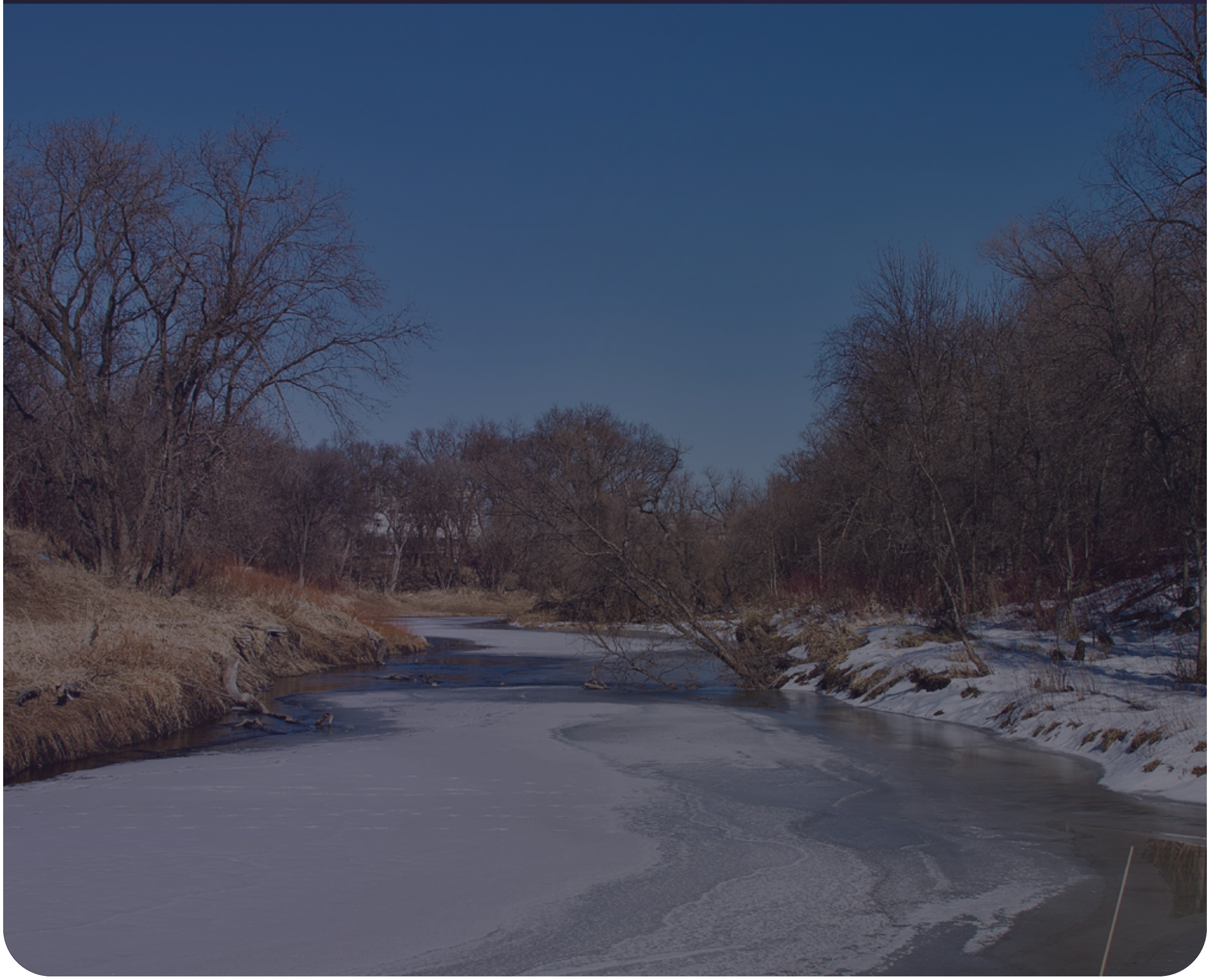

Manitoba’s strengths

- ***Infrastructure for coordination and planning*** – Manitoba’s smaller size and delivery of testing through a single service organization, Shared Health allows it to be nimble and more quickly responsive to clinical need.
- ***Innovative testing*** – Manitoba is able to anticipate future testing needs and plan for their adoption.

Manitoba’s weaknesses

- ***Finance approach*** – Manitoba has a reactive financing approach with no discretionary spending or dedicated funding for test development. There is no systemic or periodic approach to resource planning.
- ***Creating opportunities for innovation*** – Manitoba lacks a transparent evaluation function and broader engagement, which may limit innovative proposals that can benefit patients. It may also benefit from more structure and transparency outside of cancer care.

Its state of readiness to consider and adopt new tests has earned **Manitoba a grade of B-**

Takeaway: Manitoba is somewhat ready to consider and adopt new tests but would most benefit from improving its finance approach. Anticipatory planning and providing discretionary funding for the development and delivery of new tests would avoid unnecessary delays for patients.

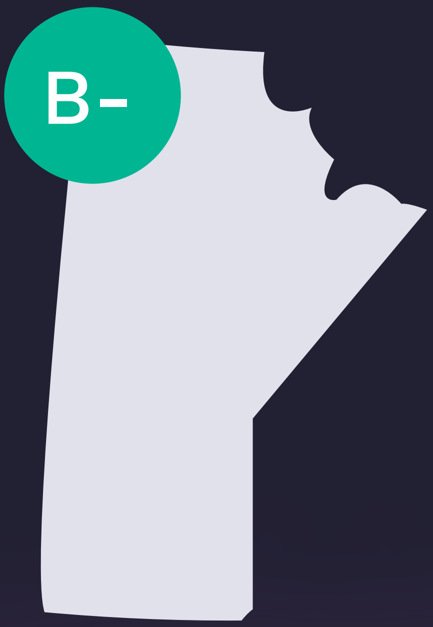

Table 4: Manitoba's progress report - readiness to consider and adopt new testing.

Condition	Assessment	Description
Creating communities of practice and healthcare system networks	Some aspects established with others in need for improvement	• Formal networks for oncology<br>• Less formal networks for non-oncology testing<br>• Limited engagement with broader stakeholders.
Resource planning	Need for improvement	• No systemic or periodic approach to resource planning
Information management	Partially established	• Some linkage of laboratory information systems but reliance on manual checking for clinical delivery
Entry/exit point for innovation	Some aspects established, and others in need for improvement	• Multiple entry points<br>• Established for oncology but needs improvement outside of oncology
Evaluative function	Some aspects established, and others in need for improvement	• Different processes for evaluation established<br>• Need for transparency
Service models	Established	• Centralized service model through Shared Health allows for coordination
Awareness and care navigation	Partially established	• Test list for oncology developed although only available internally to specialists.<br>• No list for non-oncology
Integration of Innovation and healthcare delivery	Established	• Investigational testing funded<br>• Horizon scanning and anticipatory planning for new tests
Financing approach	Some aspects established with others in need for improvement	• Adequate funding for test delivery<br>• No funding for test development or validation and no dedicated budget for discretionary spending
Education and training	Partially established	• Ongoing lab-motivated training reliant on private or grant funding<br>• No overarching educational strategy
Regulation	Established	• Province-wide accreditation standards<br>• Single lab for validation standards and proficiency through CAP or ad-hoc partnerships

New Brunswick

Canada’s eighth largest province by size and population (approx. 0.8 million), New Brunswick delivers service through two main health authorities: Horizon Health Network and Vitalité Health Network. Horizon is the largest regional health authority in the province, operating 12 hospitals and delivers service to central and southern New Brunswick. Vitalité serves the northern and southeastern regions of the province. Each health region conducts molecular testing for pathology related to cancer, while hereditary testing is primarily performed through the Maritime Medical Genetics Service (MMGS), which provides genetic services to residents of Nova Scotia, New Brunswick, and Prince Edward Island and performs testing at the Clinical Genomics Laboratory located at the IWK Health Centre in Halifax, Nova Scotia.

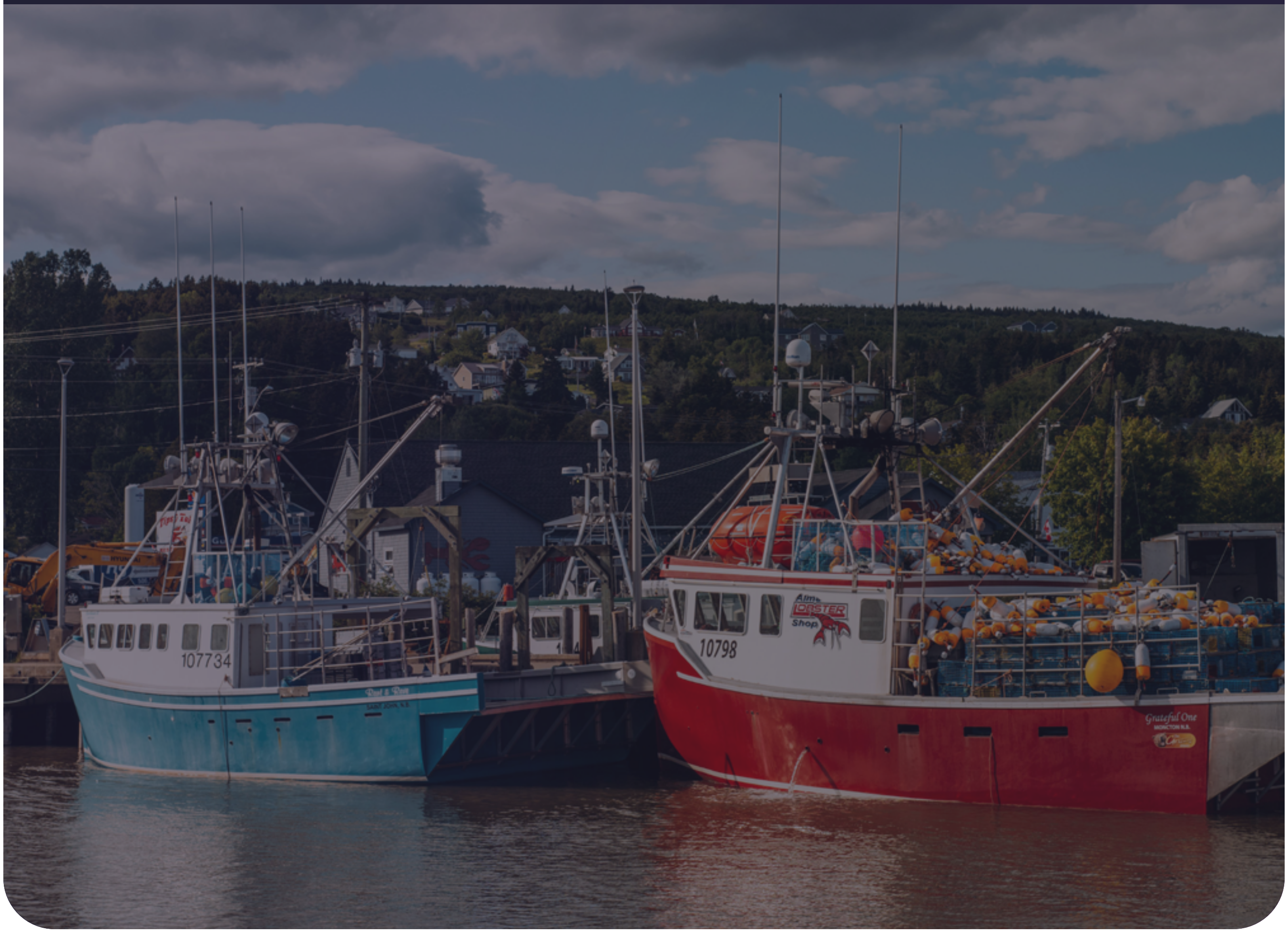

New Brunswick’s strengths

- ***Pan-provincial testing*** – Outside of cancer, New Brunswick benefits from participation in the Maritime Medical Genetics Service (MMGS), which can create efficiencies of scale and provides equitable access to care.
- ***Innovative testing*** – New Brunswick is also able to anticipate future testing needs and plan for their adoption by integrating tests ahead of mainstream adoption.

New Brunswick’s weaknesses

- ***Finance approach*** – New Brunswick has a reactive financing approach with no discretionary spending. There is no systemic or periodic approach to provincial resource planning – instead decisions are made in response to regional demands.
- ***Creating opportunities for innovation*** – New Brunswick lacks a transparent evaluation function or test list and has limited engagement with innovators, which may limit proposals that can benefit patients.

Its state of readiness to consider and adopt new tests has earned **New Brunswick a grade of B-**

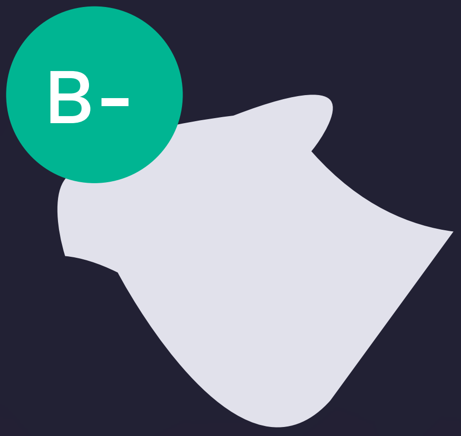

Takeaway: New Brunswick is somewhat ready to consider and adopt new tests but would most benefit from improving its finance approach. Anticipatory planning and providing discretionary funding for the development and delivery of new tests would avoid unnecessary delays for patients.

Table 5: New Brunswick's progress report - readiness to consider and adopt new testing.

Condition	Assessment	Description
Creating communities of practice and healthcare system networks	Partially established	• Informal networks for laboratories performing testing in NB<br>• Forum with innovators being discussed
Resource planning	Partially established	• Informal planning through Department of Health responses to regional requests
Information management	Some aspects established, and others in need for improvement	• Laboratory results linked to MyHealthNB portal<br>• No linkage of laboratory information systems
Entry/exit point for innovation	Established	• NB Dept of Health has an application process
Evaluative function	Partially established	• No standardized framework<br>• Need for transparency
Service models	Partially established	• Informal coordination between regions and NB Department of Health
Awareness and care navigation	Some aspects partially established, some in need of improvement	• Regular, internal communication to physicians.<br>• No province-wide test list.
Integration of Innovation and healthcare delivery	Some aspects established, some partially established	• Advanced planning and integration of investigational testing for cancer<br>• Only actionable biomarkers typically reported
Financing approach	Partially established	• Funding for test development relies on operational budget and grants<br>• No dedicated budget for discretionary spending
Education and training	Some aspects established, and others in need for improvement	• Ongoing lab-motivated training reliant on private or grant funding<br>• No overarching educational strategy
Regulation	Established	• Province-wide accreditation standards<br>• Single lab for validation standards and proficiency through CAP or ad-hoc partnerships

Newfoundland and Labrador

Canada's seventh largest province by size and ninth largest by population (approx. 0.5 million), Newfoundland and Labrador deliver health services through five health zones organized under one provincial health authority, Newfoundland and Labrador Health Services (NLHS). Genetic testing is conducted by NLHS Laboratory Medicine Program at Memorial University of Newfoundland's (MUN) Craig L. Dobbin Genetics Research Centre through the MUN Centre for Translational Genomics. Somatic testing is largely referred to out-of-country providers.

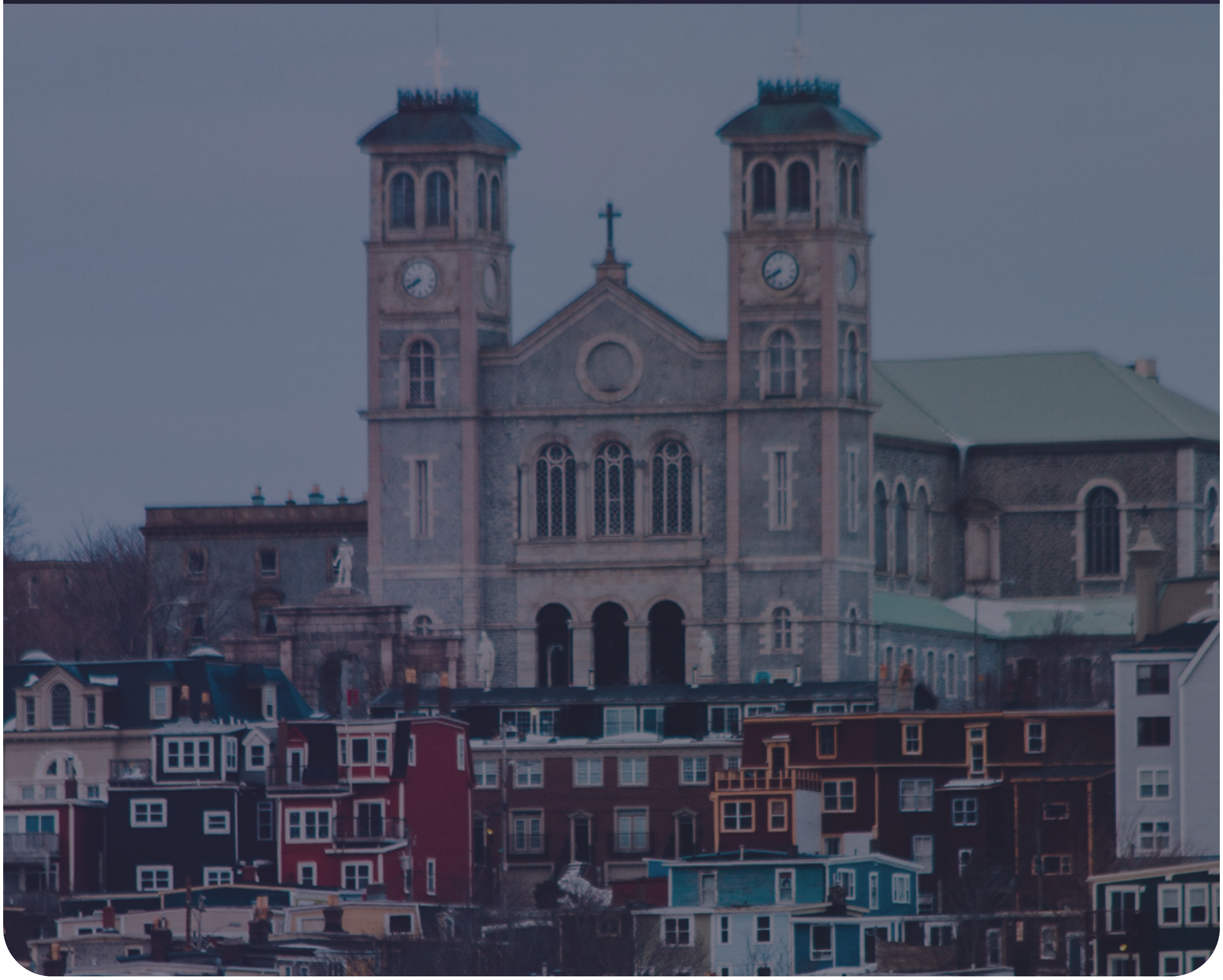

Newfoundland and Labrador's strengths

- ***Creating opportunities for innovation*** – Newfoundland has one of the most transparent processes in Canada for considering and adopting new tests across all Canadian provinces. This means it is clear to innovators, patients, providers, and the public what is considered valuable, and what is funded.
- ***Infrastructure for coordination and planning*** – Newfoundland and Labrador's smaller size and delivery of testing through a single service organization facilitates coordination and resource planning.

Newfoundland and Labrador's weaknesses

- ***Finance approach*** – Newfoundland and Labrador has a reactive financing approach with no discretionary spending. There is no anticipatory planning as decisions are made in response to lab proposals. This can slow time to adoption.
- ***Service delivery model*** – Newfoundland and Labrador's reliance on out-of-country providers ensures high quality testing but may also create challenges for patients and decision-making through longer turnaround times. This approach may also be less efficient. There are currently efforts to repatriate testing.

Its state of readiness to consider and adopt new tests has earned **Newfoundland and Labrador a grade of B+**

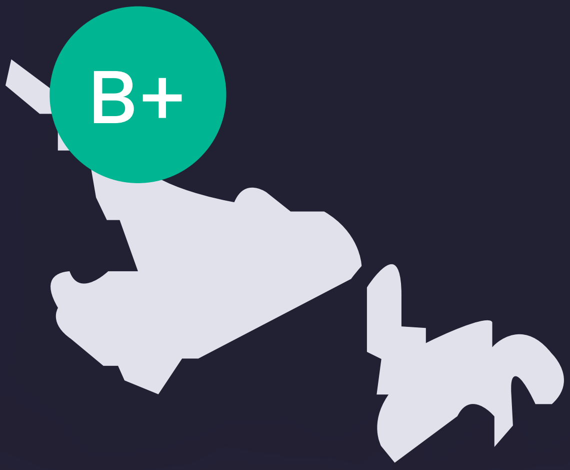

Takeaway: Newfoundland and Labrador has created many of the necessary conditions for readiness to consider and adopt new tests. Repatriation of tests as well as anticipatory planning and discretionary funding for the development and delivery of new tests would avoid unnecessary delays for patients.

Table 6: Newfoundland and Labrador's progress report - readiness to consider and adopt new testing.

Condition	Assessment	Description
Creating communities of practice and healthcare system networks	Some aspects established, and others partially established or need improving	• Formal network for germline testing established and operational.<br>• Structure for somatic testing approved and in process of operationalizing.<br>• Limited broader stakeholder engagement
Resource planning	Established	• Systemic oversight through Laboratory Medicine Program
Information management	Established	• Laboratory information system linked to hospital information system, provincial HER systems, and laboratory and point-of-care analytical instruments
Entry/exit point for innovation	Established	• Single open point of entry
Evaluative function	Some aspects established, and others in need for improvement	• Provincial Laboratory Formulary Advisory Council (PLFAC) and published criteria<br>• No broader stakeholder engagement
Service models	Established	• Coordination through a single centre<br>• Reliance on out-of-country providers
Awareness and care navigation	Established	• Provincial Laboratory Formulary publicly available.
Integration of Innovation and healthcare delivery	Established	• Investigational testing funded<br>• Translational research through Center for Translational Genomics
Financing approach	Some aspects established with others in need for improvement	• Funding for test development<br>• No dedicated budget for discretionary spending
Education and training	Some aspects partially established with others in need for improvement	• Ongoing lab-motivated training reliant on private or grant funding<br>• No overarching educational strategy
Regulation	Established	• Province-wide accreditation standards<br>• Single lab for validation standards and proficiency through CAP or ad-hoc partnerships

Nova Scotia

Canada’s ninth largest province by size and seventh largest by population (approx. 1.0 million), Nova Scotia deliver health services through one provincial health authority, Nova Scotia Health (NSH). Testing occurs within two major hospitals (Queen Elizabeth II Health Sciences Centre and IWK Health Centre) that deliver specialized care programs. Nova Scotia also uses out-of-province providers and is a host for the Maritime Medical Genetics Service (MMGS), which provides genetic services to residents of Nova Scotia, New Brunswick, and Prince Edward Island. Oversight for these programs is provided through the NSH Pathology and Laboratory Medicine Program (PLMP).

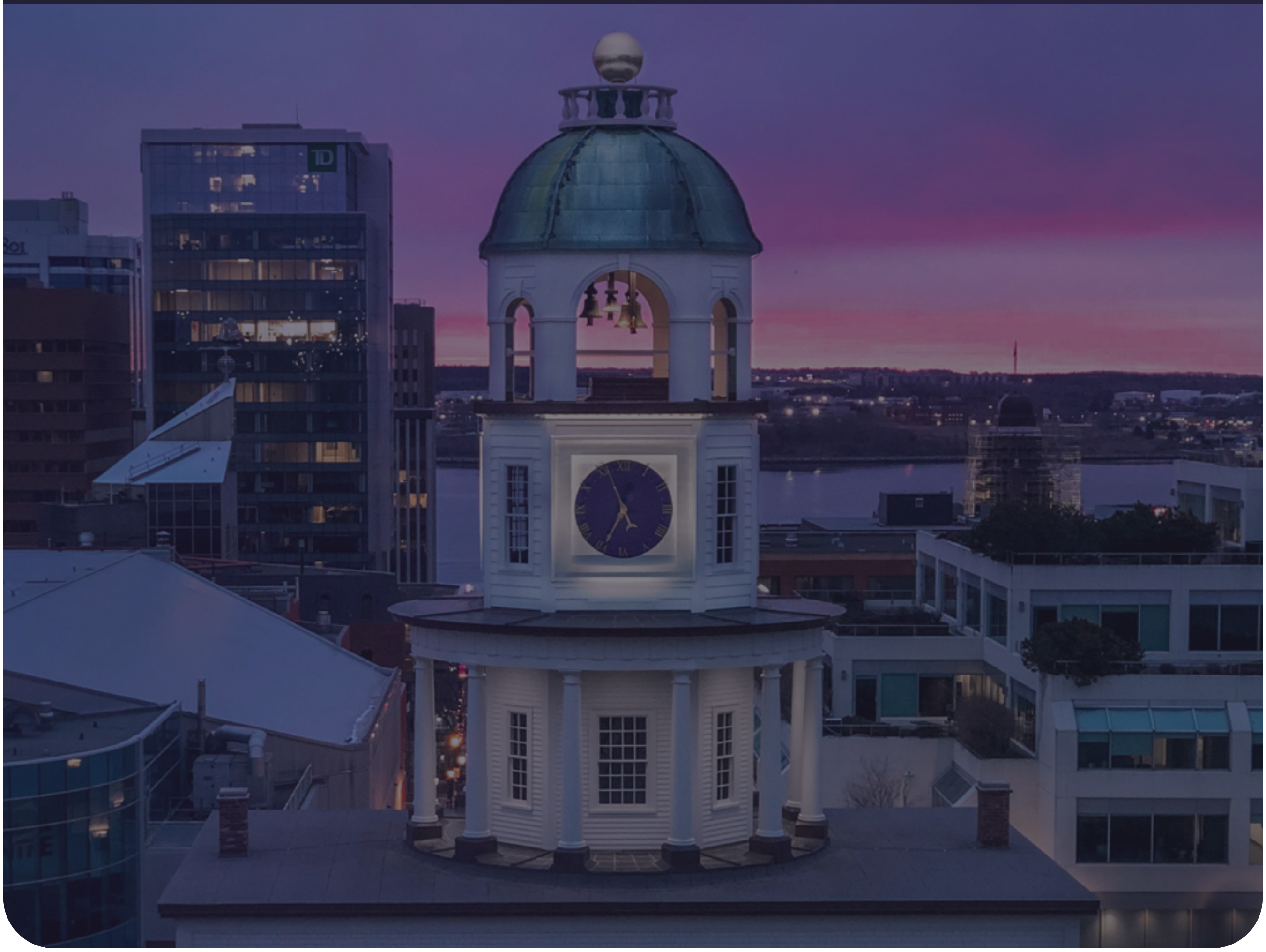

Nova Scotia's strengths

- ***Infrastructure for coordination and planning*** – Nova Scotia's smaller size and delivery of testing through a single service organization facilitates coordination and resource planning.
- ***Financing approach*** – Nova Scotia's finance approach allows it to get new testing to patients more quickly.

Nova Scotia's weaknesses

- ***Creating opportunities for innovation*** – The pathway to consideration and adoption is not transparent and has multiple routes of entry. There are no explicit timelines for consideration and broader stakeholder engagement is limited. Not all available tests are published.
- ***Information management*** – Nova Scotia still lacks linked information systems but is working toward addressing this challenge.

Its state of readiness to consider and adopt new tests has earned **Nova Scotia a grade of B**

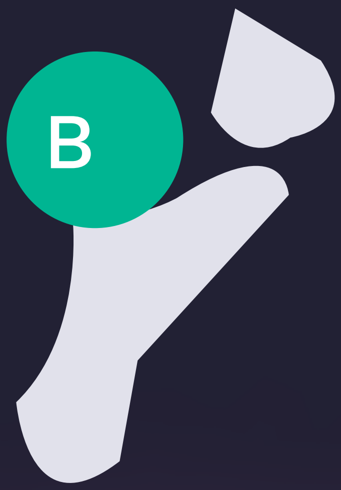

Takeaway: Nova Scotia has many of the necessary conditions for readiness to consider and adopt new tests. Creating a more transparent system for consideration and evaluation of testing coupled with broader stakeholder engagement would help patients, providers and innovators better understand what is needed and what is valuable.

Table 7: Nova Scotia’s progress report - readiness to consider and adopt new testing.

Condition	Assessment	Description
Creating communities of practice and healthcare system networks	Some aspects established, and others partially established	• Formal network within Pathology and Laboratory medicine Program (PLMP) and IWK Maritime Centre for Precision Medicine<br>• Plan for broad stakeholder engagement including patients and industry
Resource planning	Established	• Systemic oversight through PLMP
Information management*	Partially established	• Lack of integration of laboratory information for inherited diseases<br>• Provincial launch of patient portal planned – will incorporate somatic testing
Entry/exit point for innovation*	Some aspects established with others in need for improvement	• Established point of entry but other routes of entry possible<br>• No explicit timelines or reassessment process
Evaluative function*	Some aspects established, and others in need for improvement	• Impact committee established to evaluate new tests and published criteria<br>• No broader stakeholder engagement
Service models	Established	• Centralized service model facilitates coordination
Awareness and care navigation	Partially established	• Test directory<br>• Not all tests (e.g., oncology) listed
Integration of Innovation and healthcare delivery	Established	• Investigational testing funded<br>• Translational research through grant funding
Financing approach*	Established	• Funding for test development and budget for discretionary spending
Education and training*	Some aspects established, and others in need for improvement	• Ongoing lab-motivated training reliant on private or grant funding<br>• No overarching educational strategy
Regulation*	Established	• Province-wide accreditation standards<br>• Single lab for validation standards and proficiency through CAP or ad-hoc partnerships

*Has improved since 2023

Ontario

Ontario is the third largest of Canada's 10 provinces by size and largest by population (approx. 16.2 million), with the vast majority of the province's inhabitants located in its southernmost regions. Capacity for genetic testing largely resides in its hospitals. Testing for hereditary disease occurs in Ontario's two children's hospitals, London Health Sciences Centre, Hamilton Health Sciences/ St. Joseph's Healthcare Hamilton, Kingston Health Sciences Centre, Mount Sinai Hospital, North York General Hospital, Trillium Health Partners Credit Valley Site, the University Health Network, and two specialty testing centres (The National Inherited Bleeding Disorder Genotyping Laboratory and Newborn Screening Ontario). Somatic testing is conducted by an additional 7 service providers (Health Sciences North, Sunnybrook Health Sciences Centre, The Ottawa Hospital, Unity Health - St. Michael's Hospital, William Osler Health System, as well as specialized Dynacare and LifeLabs facilities). Some testing is commissioned to out-of-province providers as well. Oversight and coordination of testing is provided by the provincial genetics program (PGP) which works through the Pathology & Laboratory Medicine Program (PLMP) of Ontario Health.

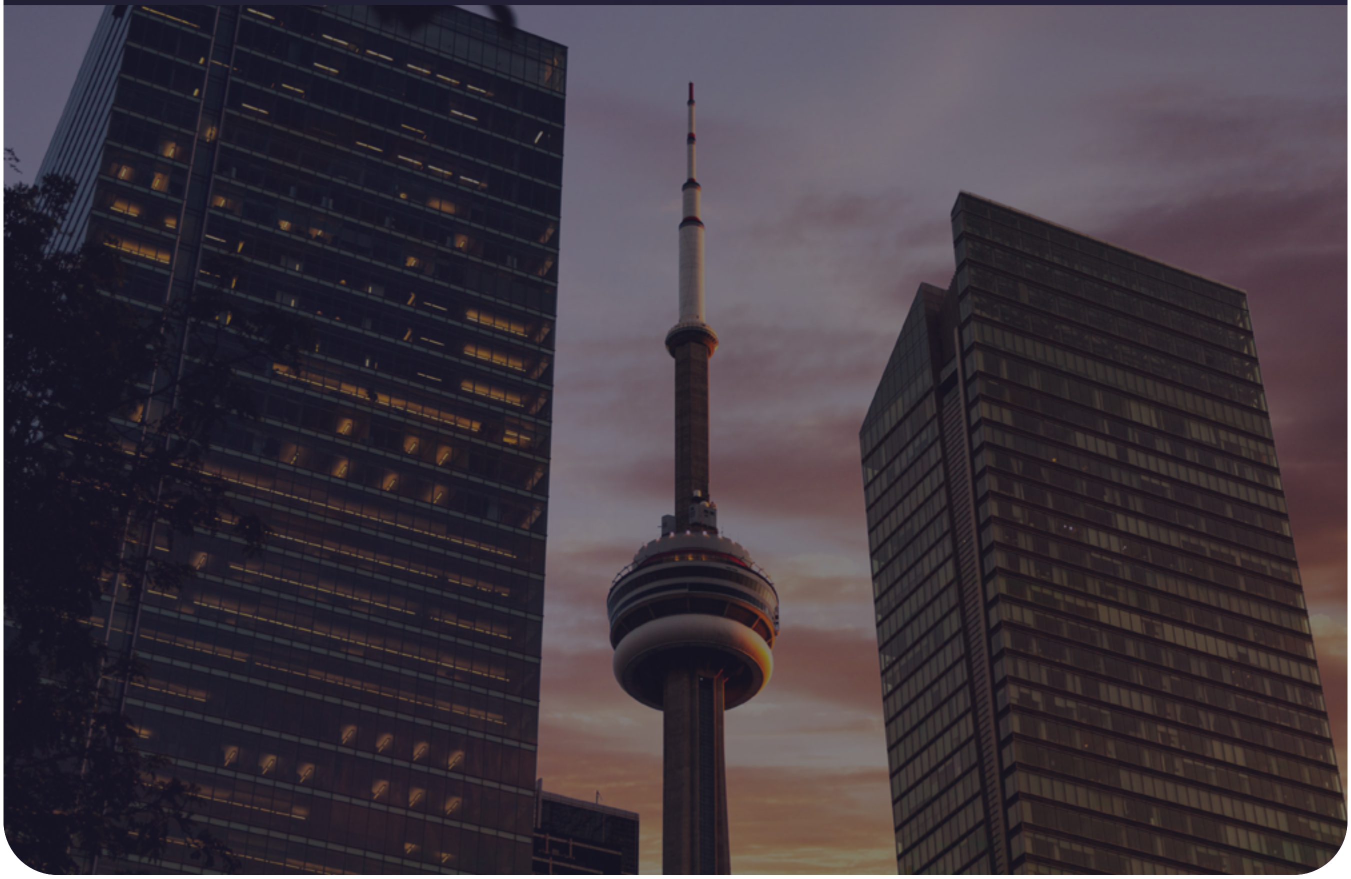

Ontario's strengths

- ***Infrastructure for coordination and planning*** – Ontario continued to leverage the PLMP program as a basis for resource planning, service coordination, care navigation, and creating province-wide standards for education.
- ***Forward looking*** – Ontario has embraced investigational testing. They have also been working toward fundamental changes in how translational research will be integrated into mainstream care.

Ontario's weaknesses

- ***Creating opportunities for innovation*** – The pathway to consideration and adoption is not transparent and has multiple routes of entry. There are no explicit timelines for consideration and broader stakeholder engagement in either evaluation or planning is limited.
- ***Infrastructure*** – Ontario still lacks linked information systems but is working toward addressing this challenge.
- ***Financing approach*** – Ontario still does not provide financing for test development.

Its state of readiness to consider and adopt new tests has earned **Ontario a grade of B**

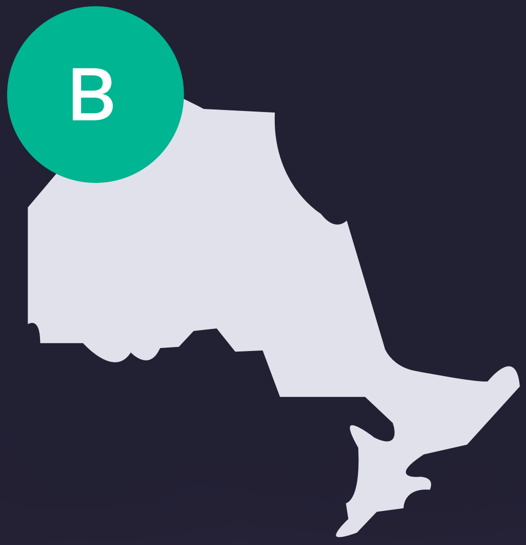

Takeaway: Ontario has many of the necessary conditions for readiness to consider and adopt new tests. Creating a more transparent system for consideration and evaluation of testing coupled with broader stakeholder engagement would help patients, providers and innovators better understand what is needed and what is valuable.

Table 8: Ontario's progress report - readiness to consider and adopt new testing.

Condition	Assessment	Description
Creating communities of practice and healthcare system networks*	Some aspects established, and others partially established	• PGP/PLMP responsible for provincial coordination<br>• No standardized process for broader stakeholder engagement
Resource planning*	Established	• Systemic oversight through PLMP
Information management*	Partially established	• Plans to bring genetic laboratory information into the Ontario Laboratories Information System (OLIS)
Entry/exit point for innovation	Some aspects established with others in need for improvement	• Coordination with Ontario Genetics Advisory Committee<br>• Process not transparent
Evaluative function	Some aspects established with others in need for improvement	• Ongoing evaluation through PGP-led expert and advisory groups (not transparent)<br>• Multiple evaluation frameworks (PGP, OGAC) with different timelines
Service models*	Established	• Coordinated through PLMP
Awareness and care navigation*	Established	• Test directories published
Integration of Innovation and healthcare delivery	Established	• Investigational testing funded<br>• Translational research through grant funding
Financing approach	Some aspects established with others in need for improvement	• No funding for test development<br>• Discretionary spending through Ontario Health
Education and training*	Established	• Strategy developed and implemented through PGP<br>• Province-wide standards available
Regulation	Some aspects established with others partially established	• Province-wide accreditation standards<br>• Analytic validity standards in development

*Has improved since 2023

Quebec

Canada’s largest province by size and second largest by population (approx. 9.1 million) began reform on its system of laboratory governance in 2011. Molecular diagnostics including low- to medium-throughput sequencing is delivered across five “clusters” operating seven supra-regional laboratories (Capitale-Nationale [CHU de Québec – Université Laval]; Estrie [CHUS – Hôpital Fleurimont]; Montréal – CHUM [CHUM and Hôpital Maisonneuve-Rosemont]; Montréal – CUSM [CUSM and Hôpital général Juif]; Montréal – CHU Sainte-Justine [CHU Sainte-Justine]) as well as the Montreal Heart Institute (MHI). The Centre québécois de génomique clinique (CQGC) in 2018, physically situated at the Centre hospitalier universitaire Sainte-Justine (CHU Sainte-Justine), was established to conduct high-throughput (exome, transcriptome or whole-genome) sequencing. Testing is also referred to out-of-province providers for rarer conditions. The Direction de Laboratoires et Imagerie Médicale (DLIM) is the Ministry Program that has been tasked with coordinating the implementation of molecular diagnostic testing across all of these centres/clusters.

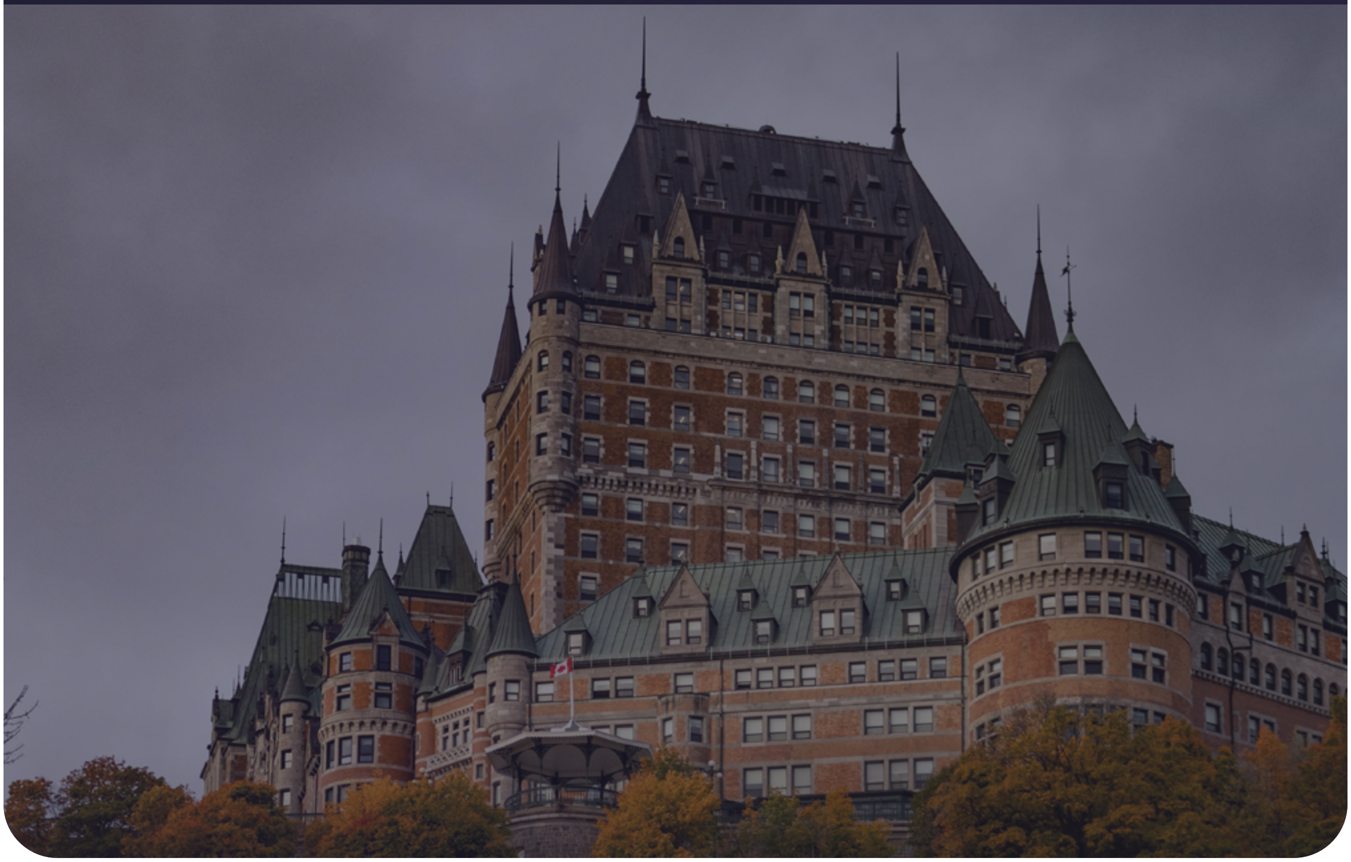

Quebec's strengths

- ***Infrastructure for coordination and planning*** – Systematic oversight and resource planning for testing is provided through the Direction de Laboratoires et Imagerie Médicale (DLIM), which in turn has created a single superregional network (Réseau Québécois de Diagnostic Moléculaire (RQDM). It additionally acts as a single point of entry for proposals for new innovation from within the health system which makes it ready to consider new testing.
- ***Financing approach*** – A clear funding formula that considers test development and additional resource costs coupled with discretionary spending for new tests means Quebec is ready to adopt testing without unnecessary delay.

Quebec's weaknesses

- ***Service models*** – Quebec's top down approach to service coordination has the potential to provide equitable access but has also resulted in documented service delays due to reliance on single centres and operational capacity.
- ***Tools to support timely and appropriate use*** – While Quebec does publish a test list ('repertoire'), it still lacks information and other supportive tools regarding available tests or access to tests. Province-wide standards for education and training have been in development since 2023.

Its state of readiness to consider and adopt new tests has earned **Quebec a grade of B-**

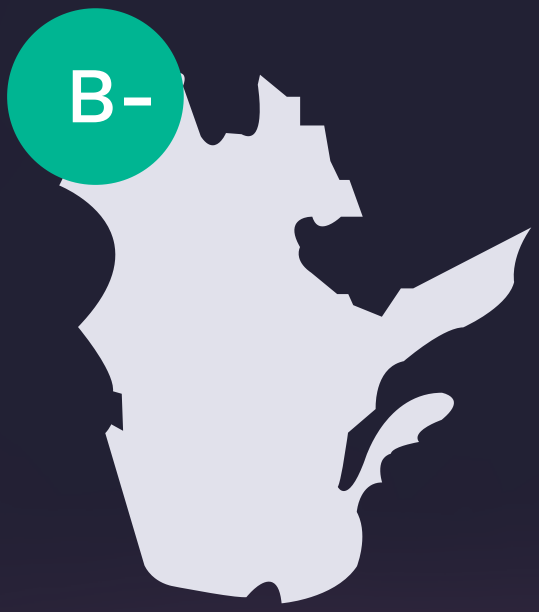

Takeaway: Quebec has created many of the necessary conditions to be ready to plan for, consider and adopt new tests. There is a need for Quebec to improve service delivery component through providing better support for navigation and capacity to test patients.

Table 9: Quebec's progress report - readiness to consider and adopt new testing.

Condition	Assessment	Description
Creating communities of practice and healthcare system networks	Some aspects established with others in need for improvement	• The RQDM acts as the supra-regional network and coordinating function<br>• Processes for broader stakeholder engagement lacking
Resource planning	Established	• Systemic oversight through DLIM
Information management	Some aspects established with others partially established	• Linked laboratory information for genetic testing planned
Entry/exit point for innovation*	Some aspects established with others partially established*	• Transparent process linked to INESSS<br>• Open application process is being developed / informal reassessment
Evaluative function	Partially established	• Well defined process with INESS<br>• Informal broader stakeholder engagement
Service models	Some aspects established with others in need of improvement	• Service coordination across providers<br>• Need for further coordination in oncology
Awareness and care navigation	Some aspects partially established with others in need of improvement	• Test directory published but lacks some information<br>• Navigation for care providers and patients lacking
Integration of Innovation and healthcare delivery	Partially established	• Translational research through Genome QC and CQGC<br>• Investigational testing not funded
Financing approach	Established	• Clear funding formula with additional funding for test development, additional and human resource costs<br>• Discretionary spending for new tests
Education and training	Partially established	• Province-wide standards for education and training in development
Regulation*	Established*	• Province-wide accreditation standards<br>• Analytic validity and other standards published

*Has improved since 2023

Saskatchewan

Canada's fifth largest province by size and sixth largest by population (approx. 1.3 million) governs testing through the Saskatchewan Health Authority's Laboratory Medicine Program and the Saskatchewan Cancer Agency. Testing is conducted at several sites including the Genetic Resource Centre at the Roy Romanow Provincial Lab (RRPL) in Regina, Genomics Laboratory, Royal University Hospital (RUH) Saskatoon as well as the Advanced Diagnostic and Research Laboratory at the University of Saskatchewan.

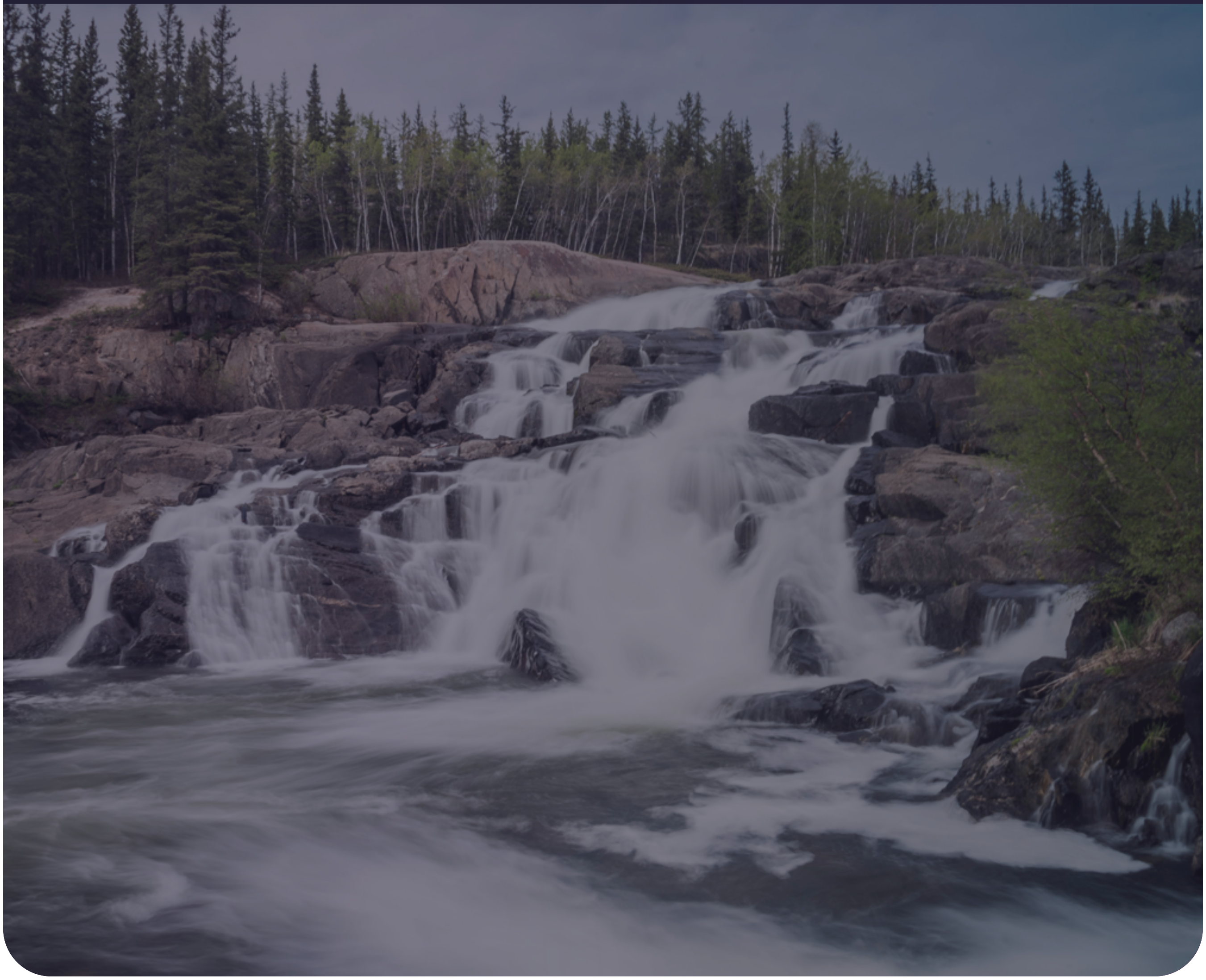

Saskatchewan's strengths

- ***Infrastructure for coordination and planning*** – Systematic oversight and resource planning for hereditary testing is provided through the Saskatchewan Health Authority; oversight for molecular testing is provided through the Saskatchewan Cancer Agency.

Saskatchewan's weaknesses

- ***Awareness and care navigation*** – Saskatchewan no longer publishes its public test formulary for genetic testing.
- ***Less readiness for hereditary testing*** – There are no formal processes for planning, or considering and evaluating new tests outside of cancer.

Its state of readiness to consider and adopt new tests has earned **Saskatchewan a grade of C**

Takeaway: Saskatchewan has created some of the necessary conditions to be ready for testing. There is a need for Saskatchewan to improve linkage of laboratory information systems and provide better support for care navigation and develop more formal processes for testing outside of cancer.

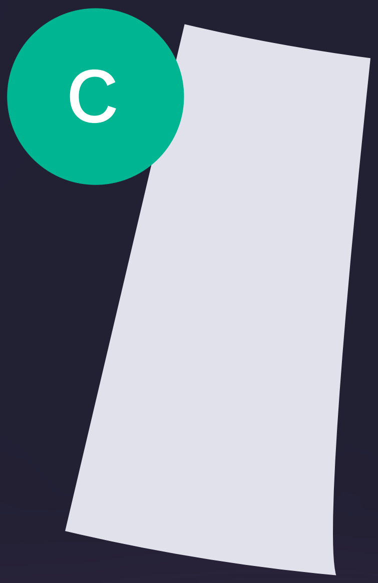

Table 10: Saskatchewan's progress report - readiness to consider and adopt new testing.

Condition	Assessment	Description
Creating communities of practice and healthcare system networks	Some aspects established with others in need for improvement	• The SHA Laboratory Medicine Program Genomics specific discipline group and SCA act as networks for care<br>• Processes for broader stakeholder engagement lacking.
Resource planning	Established	• Systemic oversight through SHA and SCA
Information management	Need for improvement	• Laboratory information systems not well linked.<br>• No link to EHR
Entry/exit point for innovation	Some aspects partially established with others in need for improvement	• Established for cancer but not transparent<br>• No similar process for non-cancer
Evaluative function	Some aspects partially established with others in need for improvement	• Molecular Biomarker Prioritization Committee (MBPC) established for cancer but not transparent<br>• Informal broader stakeholder engagement
Service models	Established	• Service coordination for cancer through Biomarker Development and Quality Assurance Committee for cancer<br>• Coordination through SHA for non-cancer
Awareness and care navigation	Need for improvement	• Test directory was previously published but now removed<br>• Navigation for care providers and patients lacking
Integration of Innovation and healthcare delivery	Partially established	• Translational research through Advanced Diagnostic and Research Laboratory<br>• Integration of innovative biomarkers but not reported
Financing approach	Some aspects established with others in need for improvement	• Discretionary spending for cancer companion biomarkers through formulary and for non-cancer biomarkers through operational funds<br>• No funding for test development
Education and training	Some aspects partially established with others in need for improvement	• Ongoing lab-motivated training reliant on private or grant funding<br>• No overarching educational strategy
Regulation	Established	• Province-wide accreditation standards<br>• Single lab for validation standards and proficiency through CAP or ad-hoc partnerships

Provinces and Territories that use referrals

Referring jurisdictions in Canada are those with no in-house capacity to provide testing. Health services are provided to their residents through cross-billing arrangements with other provinces. Hence, these provinces and territories do not necessarily need to have readiness conditions for considering and adopting new testing as it relies on their out-of-jurisdiction providers/partners.

Prince Edward Island

Prince Edward Island (PEI) is the smallest Canada's 13 provinces by size and population (approx. 0.18 million). Genetic testing is performed on a referral basis to centres in Nova Scotia.

Northwest Territories

Northwest Territories (NWT) is the second largest territory in size and second largest in population (approx. 45,000). Genetic testing is performed on a referral basis to centres in Alberta.

Nunavut

Nunavut (NU) is the largest territory in size and third largest in population (approx. 41,000). Genetic testing is performed on a referral basis to centres in Ontario, Manitoba, and Alberta.

Yukon

Yukon is the smallest territory in size and largest in population (approx. 47,000). Genetic testing is performed on a referral basis to centres in British Columbia and Alberta.

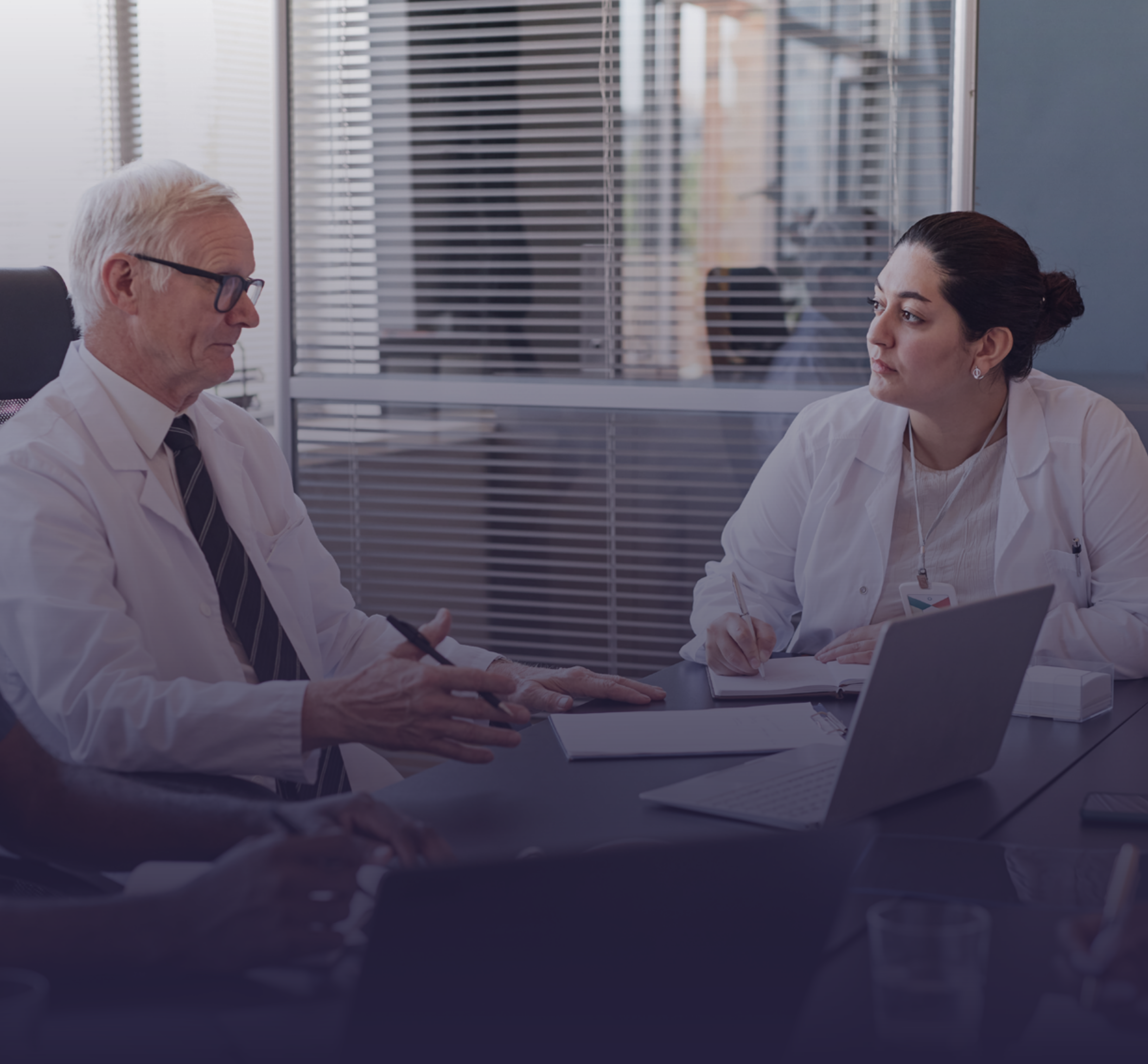

Chapter 6.

Summary of the potential impact of change and policy implications and concluding remarks

Overall, Canada appears to be making progress and is partially ready for a future of genetic and genomic testing in medicine. There are still gaps in how new testing proposals are considered and evaluated (i.e., HTA approaches), the finance approaches used, linked information systems required, and the need for more systemic educational approaches and resources to support its use, informed by overarching education strategies.

Improving HTA

Just as in 2023, more work to be done in creating world-class HTA processes (i.e., timely, responsive, transparent) for the consideration and evaluation of testing. Work toward this has culminated in the development of a pan-Canadian, consensus-based framework intended to inform adoption or funding decisions on molecular, genetic, and genomic biomarker testing in cancer care.

This framework includes a single-entry system for new proposals, clear criteria for evaluation, and some aspects of a deliberative process. The workplan calls for the establishment of "a structured process to involve and engage patients, caregivers, clinicians, laboratory professionals, jurisdictional representatives, and other key interested and impacted parties in the design and implementation of a coordinated model." A necessary first step in the development of a sound HTA process.

Strengthening finance approaches

Finance approaches continue to evolve for genomic medicine in Canada. A potential challenge in this area is that tests are not entirely funded by a single finance entity. Any given genetic or genomic test may be funded through a series of budgets including hospital operations, private and public research grants, funding from charities, funding from private service delivery, and funding from special programs, such as cancer care. Only the four largest provinces (AB, BC, ON, and QC), Saskatchewan and Nova Scotia reported having discretionary funds for the delivery of new testing. They are aided by delivering testing through single service organizations that are provided a year-to-year budget based on current volumes and future projections. Funding for test development is still largely reliant on research grants, often from private life science companies who require testing to identify patients who will go on to use a new targeted therapy. While this can be an expedient approach it can also pose challenges insofar as priority setting for new tests will rely on who is paying, rather than systemic need.

Only New Brunswick, Newfoundland and Labrador, Nova Scotia, and Quebec reported being provided funds for test development although only Nova Scotia and Quebec can access funds in anticipation of a future funding decision to use a test. In New Brunswick and Newfoundland and Labrador, test funding is provided in response to a directed proposal to deliver a new test – a potentially slower process.

Information and information management

Information management is a key focus of any laboratory system, as the regulatory requirement (ISO 15189 Standard) for the accreditation regarding the quality of delivery of lab services requires attention to laboratory information management.[46] The need for linkage of systems together or through electronic health record systems is slowly evolving. In some provinces, linkage is not required as a single lab may be responsible for all hereditary testing and another for somatic testing (as in NS or NL, for example).

Some provinces, such as BC and NB, with regional health systems, face structural and governance barriers to linkage. While ON and QC, might appear to have the advantage of linkage through a single health system, the size of their populations and service delivery function makes widescale cross-provincial information and communication technology (IT) solutions more of a challenge to implement.

Nonetheless, plans are in place in both provinces to better link laboratory systems through large scale, ongoing IT projects. Alberta is the only province delivering laboratory services from multiple providers that has fully linked laboratory information systems.

Educational standards and strategies more often required

Introducing new genome-based tests significantly changes clinical workflows, requiring targeted training that merges continuing professional development, knowledge transfer, and quality improvement frameworks.[47,48] To effectively address these needs, novel teaching approaches such as workplace-based assessments and in situ simulations are necessary.

These methods help meet the diverse contextual demands of genomic testing, which affect test accuracy and performance. Key operational factors include coordinated patient care, efficient tissue procurement and handover, carefully designed test requisitions and reports, streamlined workflows within and between services, seamless integration of electronic health records to enable automatic information exchange, and rapid, clear communication with timely feedback loops among healthcare practitioners.[49]

Frontline clinicians including family physicians and nurse practitioners will increasingly need to understand the implications of testing and be “ready” for the coming era of genomic medicine. A 2023 survey of physicians, nurse practitioners, physician assistants, and pharmacists working within the US Veteran’s Affairs health system revealed “only 20.8% of the respondents reported feeling prepared to use genetic tests and 13.0% of the respondents were currently ordering genetic tests”.[50]

Similarly, when faced with a hypothetical questions regarding the interpretation and management of genetic test results , genetic counsellors produced more correct responses than Canadian family physicians.[51] In addition to multifaceted, evidence-based educational strategies, the optimal use of testing in primary care will require further clarification regarding roles and referral pathways, along with collaboration with genetics services.

Province-specific priority actions

Conditions most established by provinces were service coordination and integration of innovation. Provinces wishing to implement future changes to improving their readiness to consider and adopt advanced testing required for genomic medicine, can consider a number of good practices from other jurisdictions.

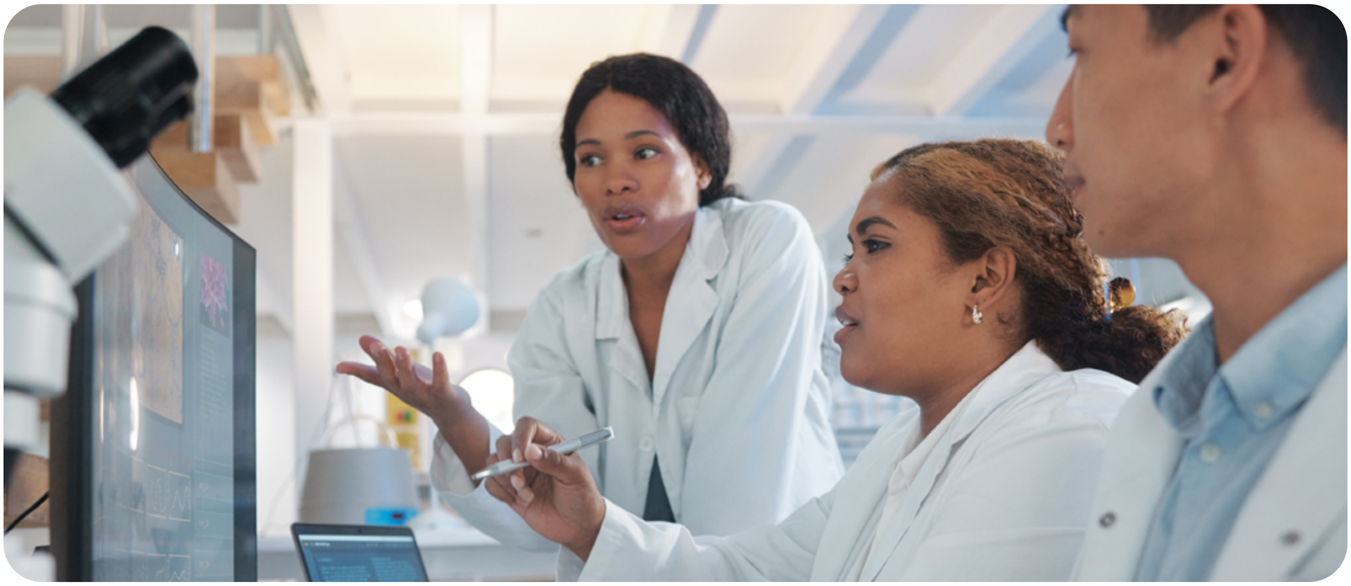

Table 11: Province-specific recommendations for the implementation of readiness conditions.

Need for improvement	Provinces needing improvement	Examples	Potential Actions
Broader stakeholder engagement in planning	All provinces	The UK NHS Accelerated Access Collaborative[1]	Collaborate with provincial life science organizations and patient organizations to create ongoing advice and a forum for commercial partnership.
Resource planning	MB, NB	US Government and Accountability Office workforce analysis[2]	Conduct and publish periodic (1-3 yr) assessments of overall or specific resource implications of growth in testing.
Information management	BC, MB, NB, NS, ON, QC, SK	Alberta Precision Laboratories integration through Connect Care	Standardize laboratory equipment or find Electronic Health Record (EHR) solutions to integrate laboratory information systems.
Entry/exit point for innovation	AB, BC, MB, NB, NS, ON, QC, SK	Newfoundland and Labrador open application process	Publish a transparent process for adding new tests.
Evaluation function	All provinces	Similar to NL open application process but involving stakeholders like ON OGAC process	Publish clear criteria, timelines and process for evaluation as in NL. Establish separate innovator advisory function or appoint industry representatives to evaluative committees as in Ontario.
Awareness and care navigation	AB, BC, MB, NB, NS, QC, SK	As in ON, NL	Publish comprehensive test formularies with instructions for obtaining tests.
Financing approach	AB, BC, MB, NB, NS, NL, ON, SK	As in QC through DLIM	Anticipate, plan and allocate future budgets to laboratory functions. Provide additional funding for test development and provide a clear funding formula that covers all resources.
Education and training	AB, BC, MB, NB, NS, NL, QC, SK	As in ON through Provincial Genetics Program	Create an overarching provincial strategy for education of care providers and patients. Create resources and programs to support education.

Looking to the future – implications for Canadian research

While many provinces appear more ready than ever to consider and adopt new testing, some still appear to be less ready to execute (i.e., deliver and monitor) testing in a consistent, high-quality manner due to a wide variety of factors. These factors range from a lack of clarity regarding care pathways and optimized workflows, to poor communication across providers (e.g., from a lack of integrated information systems), to inconsistent knowledge across providers (in part due to a lack of education, awareness and navigational resources), to a lack of specific human resources (e.g., such as laboratory technologists).

While the relative effectiveness and efficiency of delivering new tests is outside of the scope of this report, local health systems and researchers are encouraged to explore local and regional barriers to high quality implementation. In doing so, a full picture of the state of readiness for genomic medicine can be captured.

Implications for Canadian patients

Timely and equitable access to genetic and genomic testing, and the results from testing, are critical to ensuring that Canadian cancer patients have access to biomarker-informed decisions and treatments that may extend longevity and improve quality of life.

While Canada has made some degree of progress over the last few years towards readiness for genome-based testing, for individual patients living with a life-threatening illness, a slow rate of progress will not be enough. To the patient, waiting for provinces to plan for resources, education and training, or evaluation is untenable. A patient with a low expected rate of survival at three years cannot afford to wait three years.

Patients will feel they do not have the luxury of time as clinicians and researchers navigate service coordination and integration of innovation. Logistical concerns, such as insufficient information management or limited funding allocation, are seen as system failures when viewed through the lens of the individual fighting for their life. For the sake of Canadian cancer patients, all efforts to minimize the devastation caused by their cancer must be undertaken with urgency.

Further to this, disparities across healthcare jurisdictions are apparent. Patients will expect, and justifiably feel they deserve consistent access to genome-based testing across the country, in alignment with The Canada Health Act principles of universality and accessibility. Though implementation of genomic testing varies according to jurisdiction, pan-Canadian standardization could reduce the inequities associated with the current "postal code lottery" and could move toward ensuring that a patient residing in Pincher Creek will receive comparable diagnostic services to one living in Saguenay.

Precision oncology is a rapidly advancing field which is predicated on biomarker information, and it is paramount that Canada builds a future-proof process.[52] A recent comprehensive genomic testing (CGP) cost and benefit analysis commissioned by CCRAN and its patient group partners showed the implementation of universal CGP for de-novo metastatic diagnoses of stage 4 lung, colorectal, pancreatic, breast, and prostate cancers would result in 3,440 life-years gained, in addition to generating $180 million in economic contributions and $87-$134 million in healthcare savings, from 2025 - 2030 (The Conference Board of Canada, 2025).[53]

For patients, these life-years translate into tangible human needs—they may allow a metastatic cancer patient to witness a graduation or wedding of a loved one, the birth of a grandchild, or take a "bucket-list" vacation. Genomic medicine for patients, therefore, is a powerful promise of health that Canada needs to be ready for.

Patients—the consumers of our healthcare system—are critical stakeholders in health policy. Yet broad stakeholder engagement across Canada is still lacking. A final implication of this report is that engagement with patients requires revisiting. Healthcare systems need to act--- individual patients and patient organizations must be meaningfully, comprehensively, and structurally engaged to ensure that "readiness" for genomic medicine, and its subsequent implementation, is rooted in patient values, perspectives, priorities, and preferences. This will ensure a patient-focused approach to care

General remarks

This progress report suggests that Canada's major healthcare regions are moving closer toward a state of readiness for the consideration and adoption of new testing required for genomic medicine, although using different approaches and at different rates.

It highlights the many challenges that health systems face when they are required to quickly respond to a disruptive technology. Even more so, this report highlights the differences in access to care that Canadian may face when they are served by individual health regions with different priorities and health care structures. Simply knowing a technology will promote the health and welfare of Canadians is not enough; we need to have responsive and resilient healthcare systems that are able to quickly shift priorities and be able to recognize and enable value innovation.

References

1. Collaborative, N. A. A. NHS Accelerated Access Collaborative » About us. https://www.england.nhs.uk/aac/about-us/.
2. Office, U. S. G. A. Genetic Services: Information on Genetic Counselor and Medical Geneticist Workforces. https://www.gao.gov/products/gao-20-593.
3. Greenhalgh, T., Robert, G., Macfarlane, F., Bate, P. & Kyriakidou, O. Diffusion of Innovations in Service Organizations: Systematic Review and Recommendations. *Milbank Q.* **82**, 581–629 (2004).
4. Skivington, K. et al. A new framework for developing and evaluating complex interventions: update of Medical Research Council guidance. *BMJ* **374**, n2061 (2021).
5. Scott, R. H., Fowler, T. A. & Caulfield, M. Genomic medicine: time for health-care transformation. *The Lancet* **394**, 454–456 (2019).
6. Husereau, D. *et al.* Progress toward Health System Readiness for Genome-Based Testing in Canada. *Curr. Oncol.* **30**, 5379–5394 (2023).
7. Great Britain & Department of Health & Social Care. *Government Response to the House of Commons Science and Technology Committee Third Report of Session 2017-19*: *'Genomics and Genome Editing in the NHS'*. (2018).
8. Home. https://allofus.nih.gov/.
9. Husereau, D. & Villalba, E. *Towards the Routine Use of Genome-Based Testing in Canada's Largest Regions: A State of Readiness Progress Report.* (2023).
10. Navarrete-Opazo, A. A., Singh, M., Tisdale, A., Cutillo, C. M. & Garrison, S. R. Can you hear us now? The impact of health-care utilization by rare disease patients in the United States. *Genet. Med.* **23**, 2194–2201 (2021).
11. Economic cost of living with a rare disease across Europe | Report | CRA. https://www.crai.com/insights-events/publications/the-economic-cost-of-living-with-a-rare-disease-across-europe/.
12. Smith, E. J., Hill, M., Chitty, L. S. & Morris, S. Costs and cost-effectiveness of returning secondary findings from genomic sequencing based on the return of additional findings in the 100,000 Genomes Project. *Genet. Med. Off. J. Am. Coll. Med. Genet.* **27**, 101479 (2025).
13. Degeling, K. *et al.* Exome Sequencing in the Diagnostic Pathway for Suspected Rare Genetic Diseases: Does the Order of Testing Affect its Cost-Effectiveness? *Appl. Health Econ. Health Policy.* **23**, 453–466 (2025).
14. Kingsmore, S. F., Nofsinger, R. & Ellsworth, K. Rapid genomic sequencing for genetic disease diagnosis and therapy in intensive care units: a review. *NPJ Genomic Med.* **9**, 17 (2024).
15. Carey, A. S. *et al.* Rapid exome sequencing in PICU patients with new-onset metabolic or neurological disorders. *Pediatr.* Res. **88**, 761–768 (2020).

16. Wells, C. F. *et al.* Rapid exome sequencing in critically ill infants: implementation in routine care from French regional hospital's perspective. *Eur. J. Hum. Genet. EJHG* **30**, 1076–1082 (2022).

17. Olde Keizer, R. A. C. M. *et al.* Rapid exome sequencing as a first-tier test in neonates with suspected genetic disorder: results of a prospective multicenter clinical utility study in the Netherlands. *Eur. J. Pediatr.* **182**, 2683–2692 (2023).

18. Stark, Z. & Scott, R. H. Genomic newborn screening for rare diseases. *Nat. Rev. Genet.* **24**, 755–766 (2023).

19. Friedman, J. M. *et al.* Genomic newborn screening: public health policy considerations and recommendations. *BMC Med. Genomics* **10**, 9 (2017).

20. Howard, H. C. *et al.* Whole-genome sequencing in newborn screening? A statement on the continued importance of targeted approaches in newborn screening programmes. *Eur. J. Hum. Genet. EJHG* **23**, 1593–1600 (2015).

21. White, S. *et al.* Expanding the Australian Newborn Blood Spot Screening Program using genomic sequencing: do we want it and are we ready? *Eur. J. Hum. Genet. EJHG* **31**, 703–711 (2023).

22. Milko, L. V. *et al.* Evaluating parents' decisions about next-generation sequencing for their child in the NC NEXUS (North Carolina Newborn Exome Sequencing for Universal Screening) study: a randomized controlled trial protocol. *Trials* **19**, 344 (2018).

23. Lewis, M. A. *et al.* Supporting Parental Decisions About Genomic Sequencing for Newborn Screening: The NC NEXUS Decision Aid. *Pediatrics* **137 Suppl 1**, S16-23 (2016).

24. Roman, T. S. *et al.* Genomic Sequencing for Newborn Screening: Results of the NC NEXUS Project. *Am. J. Hum. Genet.* **107**, 596–611 (2020).

25. Ceyhan-Birsoy, O. *et al.* Interpretation of Genomic Sequencing Results in Healthy and Ill Newborns: Results from the BabySeq Project. *Am. J. Hum. Genet.* **104**, 76–93 (2019).

26. Pavey, A. R. *et al.* Utilization of genomic sequencing for population screening of immunodeficiencies in the newborn. Genet. Med. Off. J. *Am. Coll. Med. Genet.* **19**, 1367–1375 (2017).

27. Guzauskas, G. F. *et al.* Population Genomic Screening for Three Common Hereditary Conditions : A Cost-Effectiveness Analysis. *Ann. Intern. Med.* **176**, 585–595 (2023).

28. Zhang, L. *et al.* Population genomic screening of all young adults in a health-care system: a cost-effectiveness analysis. *Genet. Med.* **21**, 1958–1968 (2019).

29. Ediae, G. U. *et al.* ThinkRare: A search algorithm to identify patients with undiagnosed rare genetic disease in an electronic medical record. *Genet. Med. Off. J. Am. Coll. Med. Genet.* **27**, 101570 (2025).

30. (9) Bringing AI-Powered Rare Disease Solutions to Canada | LinkedIn. https://www.linkedin.com/pulse/bringing-ai-powered-rare-disease-solutions-canada-saventic-xcnzf/.

31. Nicholas, C. *et al.* Point of Care Liquid Biopsy for Cancer Treatment-Early Experience from a Community Center. *Cancers* **16**, 2505 (2024).

32. Sheffield, B. S. *et al.* Toward Timely and Equitable Advanced Biomarker Testing for Patients with Metastatic Cancer in Canada. *Curr. Oncol. Tor. Ont* **32**, 141 (2025).

33. Johnston, K. M. *et al.* Comprehensive genomic profiling for non-small-cell lung cancer: health and budget impact. *Curr. Oncol. Tor. Ont* **27**, e569–e577 (2020).

34. Patel, Y. P., Husereau, D., Leighl, N. B., Melosky, B. & Nam, J. Health and Budget Impact of Liquid-Biopsy-Based Comprehensive Genomic Profile (CGP) Testing in Tissue-Limited Advanced Non-Small Cell Lung Cancer (aNSCLC) Patients. *Curr. Oncol. Tor. Ont* **28**, 5278–5294 (2021).

35. Bidard, F.-C. *et al.* First-Line Camizestrant for Emerging ESR1-Mutated Advanced Breast Cancer. *N. Engl. J. Med.* **393**, 569–580 (2025).

36. Tissue-free circulating tumor DNA assay and patient outcome in a phase III trial of FOLFOX-based adjuvant chemotherapy (Alliance N0147). - ASCO. https://www.asco.org/abstracts-presentations/ABSTRACT502594.

37. Wang, X., Beharry, A., Sheffield, B. S. & Cheema, P. K. Feasibility of Point-of-Care Genomic Profiling in the Diagnosis and Treatment of Cancer of Unknown Primary. *The Oncologist* **28**, 474–478 (2023).

38. Krämer, A. *et al.* Molecularly guided therapy versus chemotherapy after disease control in unfavourable cancer of unknown primary (CUPISCO): an open-label, randomised, phase 2 study. L*ancet Lond. Engl.* **404**, 527–539 (2024).

39. Tessier-Cloutier, B. *et al.* The impact of whole genome and transcriptome analysis (WGTA) on predictive biomarker discovery and diagnostic accuracy of advanced malignancies.*J. Pathol. Clin. Res.* **8**, 395–407 (2022).

40. Nikanjam, M., Kato, S., Allen, T., Sicklick, J. K. & Kurzrock, R. Novel clinical trial designs emerging from the molecular reclassification of cancer. *CA. Cancer J. Clin.* **75**, 243–267 (2025).

41. Piedade, A. P., Butler, J., Eyre, S. & Orozco, G. The importance of functional genomics studies in precision rheumatology. *Best Pract. Res. Clin. Rheumatol.* **38**, 101988 (2024).

42. Kalliolias, G. D. & Papavassiliou, A. G. Advancing precision rheumatology through tissue and blood profiling. *Nat. Rev. Rheumatol.* **20**, 391–392 (2024).

43. Aypek, H., Aygormez, O. & Caliskan, Y. Genomics in Pancreas–Kidney Transplantation: From Risk Stratification to Personalized Medicine. *Genes* **16**, 884 (2025).

44. Liu, C., Zhang, C. & Glatt, S. J. Psychiatric Genomics 2025: State of the Art and the Path Forward. *Psychiatr. Clin. North Am.* **48**, 217–240 (2025).

45. Sanghvi, M. M. *et al.* Using Genomics to Develop Personalized Cardiovascular Treatments. Arterioscler. *Thromb. Vasc. Biol.* **45**, 866–881 (2025).

46. Yeste, M. L. L. *et al.* Management of post-analytical processes in the clinical laboratory according to ISO 15189:2012. Considerations about the management of clinical samples, ensuring quality of post-analytical processes, and laboratory information management. *Adv. Lab. Med. Av. En Med. Lab.* **2**, 373–380 (2021).

47. Chou, A. F., Duncan, A. R., Hallford, G., Kelley, D. M. & Dean, L. W. Barriers and strategies to integrate medical genetics and primary care in underserved populations: a scoping review. *J. Community Genet.* **12**, 291–309 (2021).

48. Dunlop, K. L. A. *et al.* Building capacity for genomics in primary care: a scoping review of practitioner attitudes, education needs, and enablers. *Front. Med.* **12**, 1577958 (2025).

49. Cheema, P. K. *et al.* Consensus recommendations for optimizing biomarker testing to identify and treat advanced EGFR-mutated non-small-cell lung cancer. *Curr. Oncol. Tor. Ont* **27**, 321–329 (2020).

50. Scheuner, M. T. *et al.* Genetics professionals are key to the integration of genetic testing within the practice of frontline clinicians. *Genet. Med.* **25**, 103–114 (2023).

51. Skinner, S. J., Clay, A. T., McCarron, M. C. E. & Liskowich, S. Interpretation and management of genetic test results by Canadian family physicians: a multiple choice survey of performance. *J. Community Genet.* **12**, 479–484 (2021).

52. Mateo, J. *et al*. Delivering precision oncology to patients with cancer. *Nat. Med*. **28**, 658–665 (2022).

53. Genomic Profiling for Stage 4 Cancer—November 2025. *The Conference Board of Canada* https://www.conferenceboard.ca/product/genomic-profiling-for-stage-4-cancer_nov2025/ (2025).
